# Stepwise Amplification of Circularly Polarized Luminescence in Chiral Metal Cluster Ensembles

**DOI:** 10.1002/advs.202207660

**Published:** 2023-02-25

**Authors:** Jia‐Yin Wang, Yubing Si, Xi‐Ming Luo, Zhao‐Yang Wang, Xi‐Yan Dong, Peng Luo, Chong Zhang, Chunying Duan, Shuang‐Quan Zang

**Affiliations:** ^1^ State Key Laboratory of Fine Chemicals Zhang Dayu College of Chemistry Dalian University of Technology Dalian 116024 China; ^2^ College of Chemistry Zhengzhou University Zhengzhou 450001 China; ^3^ College of Chemistry and Chemical Engineering Henan Polytechnic University Jiaozuo 454000 China

**Keywords:** chirality transfer, circularly polarized luminescence, hierarchical assembly, silver clusters

## Abstract

Chiral metal‐organic frameworks (MOFs) are usually endowed by chiral linkers and/or guests. The strategy using chiral secondary building units in MOFs for solving the trade‐off of circularly polarized luminescence (CPL)‐active materials, high photoluminescence quantum yields (PLQYs) and high dissymmetry factors (|*g*_lum_|) has not been demonstrated. This work directionally assembles predesigned chiral silver clusters with ACQ linkers through reticular chemistry. The nanoscale chirality of the cluster transmits through MOF's framework, where the linkers are arranged in a quasi‐parallel manner and are efficiently isolated and rigidified. Consequently, this backbone of chiral cluster‐based MOFs demonstrates superb CPL, high PLQYs of 50.3%, and |*g*_lum_| of 1.2 × 10^−2^. Crystallographic analyses and DFT calculations show the quasi‐parallel arrangement manners of emitting linkers leading to a large angle between the electric and magnetic transition dipole moments, boosting CPL response. As compared, an ion‐pair‐direct assembly without interactions between linkers induces one‐ninth |*g*_lum_| and one‐sixth PLQY values, further highlighting the merits of directional arrangement in reticular nets. In addition, a prototype CPL switching fabricated by a chiral framework is controlled through alternating ultraviolet and visible light. This work is expected to inspire the development of reticular chemistry for high‐performance chiroptical materials.

## Graphical abstract

Chiral transfer and circularly polarized luminescence activity are successfully achieved by assembling ACQ molecules with chiral Ag clusters in chiral silver cluster‐based assembled materials. The chiral reticular self‐assembly exhibits markedly enhanced luminescence efficiency and *g*_lum_ values compared to those of the ion‐pair‐direct assembly through a well‐defined spatial arrangement and highly efficient synergy.

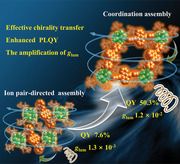

## 1 Introduction

Chirality, a ubiquitous characteristic of nature, is observed at various hierarchical levels, including the subatomic, molecular, nanoscopic, and galactic scales.^[^ ^1^ ^]^ Circularly polarized luminescence (CPL),^[^ ^2^ ^]^ which stems from the excited states of chiral fluorophore, is important in 3D display, information storage, and chiroptical switching.^[^ ^3^ ^]^ However, most chiral luminescent organic molecules always suffer from the burdensome asymmetric synthesis,^[^ ^4^ ^]^ small |*g*_lum_| values generally in the 10^−5^ to 10^−3^ ranges,^[^ ^5^ ^]^ and the undesirable ACQ effect.^[^ ^6^ ^]^ To satisfy the two key requirements of CPL, scientists developed some strategies to improve PLQY and |*g*_lum_| of small molecules.^[^ ^7^ ^]^ Structurally, with the aid of soft template, including polymer,^[^ ^8^ ^]^ liquid crystals,^[^ ^9^ ^]^ and *π*‐conjugated molecules,^[^ ^10^ ^]^ supramolecular helical assembly could efficiently amplify PLQY and |*g*_lum_| of small molecule and nanoemitters.^[^ ^11^ ^]^ Hard porous chiral templates, such as metal‐organic frameworks (MOFs),^[^ ^12^ ^]^ which assembles metal‐based secondary building units (SBUs) by organic linkers with geometrical direction into a crystalline porous extended structure through reticular chemistry,^[^ ^13^ ^]^ have also improved CPL by their chiral confinement of diverse luminescent chromophores in their pores.^[^ ^14^ ^]^ In addition, in these chiral ensembles, Förster resonance energy transfer and triplet–triplet annihilation‐based photon upconversion have been shown to effectively enlarge *g*_lum_.^[^ ^15^ ^]^ However, for these composite systems consisting of multiple components, two intrinsically urgent issues emerged: lacking the homogeneity for complete reproducibility and well‐defined structure for understanding the origin of CPL amplification at the molecular level.

Polynuclear SBUs^[^ ^16^ ^]^ provide directional linkers for MOFs, leading to unique framework properties of chemistry and physics.^[^ ^17^ ^]^ Developing chiral polynuclear SBUs to control the ordered spatial arrangement of accessible luminescent linkers will provide a platform for excellent CPL‐active MOFs, where molecular precision^[^ ^18^ ^]^ in crystallography facilitates the exploration of the origin of enlarged CPL. Metal clusters^[^ ^19^ ^]^ have tens of metal atoms, which feature multiplicity and variation in protecting ligands,^[^ ^20^ ^]^ including S‐, N‐, O‐, and C‐based ligands.^[^ ^21^ ^]^ Chiral metal clusters represent a new type of nanoscale chiral inorganic materials.^[^ ^22^ ^]^ It will be a new strategy to obtain CPL‐active materials using chiral metal clusters serving as SBUs to direct the assembly of accessible luminescent linkers in highly ordered framework structure. Recently, Prof. Zheng group reported a CPL MOF incorporating chiral Ag_14_ and chiral N‐containing linkers, yet the CPL amplification in the host framework remains elusive.^[^ ^23^ ^]^

In this work, using chiral O‐containing ligand, enantiopure camphorsulfonic acid (CSA^−^), we first prepared chiral enantiomeric single crystals [Ag_12_(S*^i^* Pr)_6_(D/L‐CSA)_6_(MeOH)_4_]*_n_* (**1a** and **1b**), in which the Ag_12_‐clusters are connected by chiral CSA^−^. To cut the intercluster linkage, we used pyridine (py) molecules, generating another pair of isolated enantiomeric silver clusters [Ag_12_(S*^i^* Pr)_6_(D/L‐CSA)_6_(py)_7_(H_2_O)][Ag_12_(S*^i^* Pr)_6_(D/L‐CSA)_6_(py)_8_]∙(py)_3_(H_2_O)*_x_* (**2a** and **2b**). These two enantiomeric chiral cluster molecules demonstrate mirror‐image cotton effects, yet have not room‐temperature emission, and certainly no CPL response was detected. The isolated chiral cluster molecules in **2a** and **2b**, in which thiolate ligand aid Ag ions to clustering, D/L‐CSA^−^ endowing chirality, and py ligands could be replaced by other py‐linker,^[^ ^24^ ^]^ encouraged us to assemble such chiral cluster nodes with appropriate linkers to investigate the CPL generation and amplification. The chiral silver clusters in **1** and **2** were used as the basic SBUs for the following assembly by reticular chemistry.

**Figure 1:**
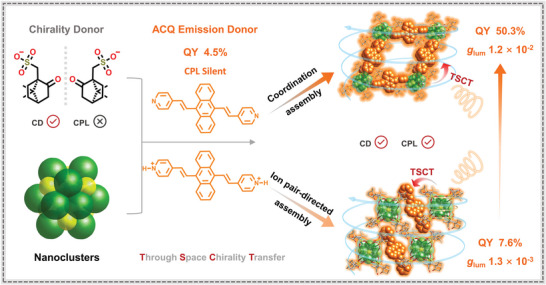
Schematically illustrating a chiral reticular self‐assembly that displayed effective chirality transfer, enhanced luminescent efficiency, and amplified *g*_lum_ values compared with the chiral‐ion‐pair‐directed assembly.

9,10‐bis(4‐Pyridylethenyl)anthracene (An2Py), which is a typical achiral ACQ molecule, was selected to assemble the above‐predesigned chiral silver cluster. As respected, we succeeded in fostering the third enantiomeric single crystals of 2D CMOFs, {[(Ag_12_(S*^i^* Pr)_6_(D/L‐CSA)_6_(An2Py)_3_)]∙(H_2_O)_2_}*_n_* (**3a** and **3b**). Excitingly, **3a** displayed high PLQY of 50.3% and |*g*_lum_| of 1.2 × 10^−2^. To examine the possible causes of the enlarged CPL in **3a**, we prepared the fourth group of chiral single crystals, [Ag_12_(S*^i^* Pr)_6_(D/L‐CSA)_8_]∙(H_2_An2Py)(solvent)*_x_* (**4a·solvent** and **4b·solvent**), as control materials through ion‐pair cocrystallization method. **4a** only displayed one‐sixth of PLQY and one‐ninth of |*g*_lum_| of chiral **3a** CMOFs (**Figure** **1**). Crystallographic analyses and density functional theory (DFT) calculations showed that the excellent CPL activity was ascribable to the quasi‐parallel assembly mode of the ACQ linker in the reticular nets, which led to a large angle between the electric and magnetic transition dipole moments. Moreover, using of excellent CPL performance of the chiral **3** MOFs, we developed a sandwich‐like mixed‐matrix‐membrane (MMM) strategy for designing chiroptical switches that operate under alternating ultraviolet and visible‐light, which involves integrating a spirooxazine, a chiral framework, and polydimethylsiloxane (PDMS).

## 2 Results and Discussion

### 2.1 Synthesis and Crystal Structures

**Figure 2:**
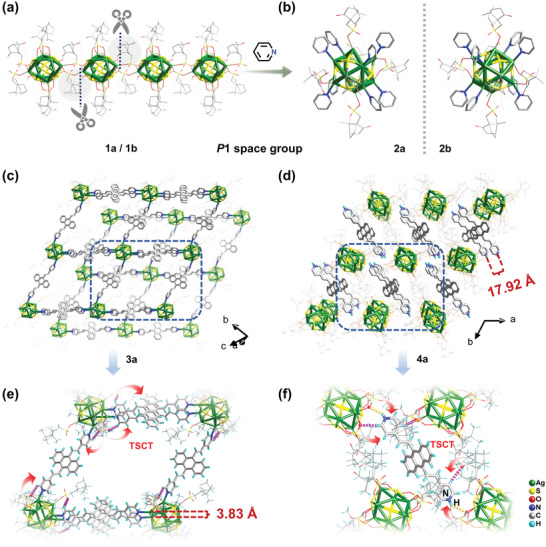
Structural representations of a) the chiral 1D chain of **1a**, b) chiral silver clusters of **2**, and silver‐cluster‐assembled materials of c) **3a** through coordination bonding and d) **4a** through supramolecular interactions. e,f) weak C–H···O An2Py/CSA^−^ interactions in **3a** and **4a** (pink dashed lines) and N‒H⋅⋅⋅Ο An2Py/CSA^−^ interactions in **4a** (orange dashed lines).

Single‐crystal X‐ray diffraction analyses revealed that all complexes crystallized in the chiral triclinic *P*1 space group and possessed Ag_12_ cores (**Figure** **2**; Tables S3–S6, Supporting Information). Colorless block crystals of **1** were formed by the reaction of (S*^i^* PrAg)*_n_* and CSA‐Ag in DCM/MeOH mixture solvents at ambient temperature. The silver atoms were rapidly gathered by CSA^‒^ and S*^i^* Pr^‒^ to form Ag_12_ clusters via coordination bonding and argentophilic interaction. These formed Ag_12_ clusters were further linked by CSA^‒^ ligands resulting in extensive 1D coordination polymer. The Ag_12_ cluster is an empty cuboctahedron, which is linked together with Ag^I^···Ag^I^ argentophilic interactions. Each S*^i^* Pr^‒^ ligand is linked to four adjacent Ag(I) ions in *µ*_4_–*η* ^1^, *η* ^1^, *η* ^1^, *η* ^1^ coordination modes, with CSA^−^ adopting two different coordination modes (*µ*_2_–*η* ^1^, *η* ^1^, *µ*_3_–*η* ^1^, *η* ^1^, *η* ^1^) (Figure S1, Supporting Information). The cuboctahedral silver cluster, with four Ag(I) vertices each bearing a terminal MeOH molecule, is linked to adjacent two silver clusters via CSA^−^ forming a 1D chain along the *a*‐axis in **1**. Compared with coordinated solvent molecules, pyridine ligands have greater steric hindrance, which may prevent camphorsulfonic acid bonding to adjacent Ag clusters and thus break the intercluster linkage. Subsequent addition of pyridine to the solution system yielded block crystals of **2** that possessed the same characteristic Ag_12_ core structure as **1**, in which the Ag_12_ units were coprotected with CSA^−^ and pyridine ligands (Figure 2b). The extension of the monodentate pyridine ligand to the bidentate ligand An2Py is a judicious choice because of its versatility in assembling cluster‐based MOFs. Enantiomeric **3a** and **3b** were obtained in a 2D layered architecture, in which the Ag_12_ cluster is connected to four neighbors through a total of six bidentate linkers (An2Py), with rhombic grids in an *ABAB* packing pattern (Figure 2c; Figure S2, Supporting Information). The four connected nodes (Ag_12_ cluster) were modified with six homochiral auxiliary ligands and extended with bidentate linkers to form a chiral 2D framework structure with perfect mirror symmetry, which can be simplified to a (2,4)‐connected structure with an *sql*‐type topology (Figure S3, Supporting Information). Fortunately, **4a·solvent** and **4b·solvent** were obtained by the coassembly of Ag_12_ cluster nodes and ACQ molecules using a pair of enantiomerically pure chiral CSA^−^. In contrast, the silver cluster in **4a·solvent** is protected by sulfonic acids and thiol ligand, while the *N*‐containing ligand An2Py is dispersed into the interstitial spaces of the Ag_12_ lattice following concomitant protonation of its pyridine moieties (Figure 2d). It indicated that the isolated clusters can also be obtained by using protonated An2Py with large steric hindrance. The two pyridyl groups in the An2Py ligand dimer in **3a** are twisted from the anthracene ring; however, the neighboring anthracene rings are quasi‐parallel due to crystallographic symmetry and separated by 3.81 Å (Figure S4, Supporting Information). On the other hand, the closest distance between adjacent An2Py anthracene rings in **4a·solvent** is 17.92 Å, which is much larger than that in **3a**, consistent with the existence of barely any interaction. In addition, many C–H···O and N–H···O (An2Py/CSA^−^) interactions exist in the assemblies (Figures 2e,f) that ensure high chiral‐transmission fidelity.^[^ ^25^ ^]^ These molecules of solvent have escaped from **4·solvent** by exposing to the atmosphere, producing **4**. It showed no visible change in the characteristic UV–vis absorption and photoluminescence properties compared with **4·solvent**. It has been confirmed that **3** and **4** retained their constant framework structure by the consistency between the simulated and experimental PXRD patterns (Figure S5, Supporting Information). The chemical formulae of **3** and **4** were further verified by elemental analysis and TG (Figure S6, Supporting Information). All the tests of the discussed optical properties were carried out on **3** and solvent‐free **4**.

### 2.2 Optical Properties

**Figure 3:**
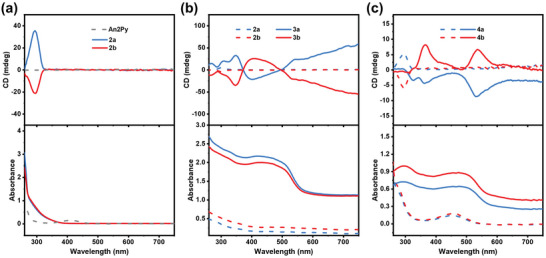
a) CD and corresponding UV absorption spectra of **2** in methanol and the An2Py luminophores in THF. b) CD and corresponding UV absorption spectra of **2** (short dashed‐dotted traces) and **3** (solid traces) in the solid state. c) CD and corresponding UV absorption spectra of **4** in methanol (short dashed‐dotted traces) and the solid state (solid traces).

The photophysical properties of the prepared chiral clusters and assembly materials were investigated at room temperature. UV‐vis absorption spectroscopy reveals that colorless **2a** absorbs in the UV region at wavelengths below 370 nm. The CD spectra of enantiomers **2a** and **2b** (**Figure** **3**a) display intense Cotton effects in the 260–370 nm range in MeOH. The UV‐vis absorption spectra of **3a** and **4a** absorb both UV and visible light in the solid state (Figure S7, Supporting Information). They show slight bathochromic shifts in the optical absorption band edges compared to that of An2Py. CD spectra of the enantiomers of **3** displayed intense Cotton effects and an excellent mirror‐image relationship in the 240–530 nm range (Figure 3b; Figure S8, Supporting Information). Poly(methyl methacrylate) (PMMA) films of **3** were prepared and subjected to CD in the same range to effectively eliminate the effect of scattering. Notably, the polymer‐embedded state exhibited PXRD, IR, and Raman spectra, as well as optical properties that are almost identical to those of the powder crystal state (Figures S9–S11, Supporting Information), which indicates that the CD spectra of the polymer‐embedded state of **3** originated from electronic interactions in a nonfluid solid solution (i.e., PMMA), demonstrating that the structure successfully induces chirality. Notably, the CD signals are mainly attributable to the absorption of the framework, while the CD signals of **3** in the 530–750 nm range are mainly generated by the absorption of the chromophore in the assembled material (Figure S12, Supporting Information).^[^ ^26^ ^]^ CD spectra of **4a** and **4b** were acquired both in methanol solution and in the solid state. In methanol, **4a** and **4b** present CD signals that are similarly to those of **2a** and **2b**. In contrast, **4a** and **4b** displayed Cotton effects in the 250–580 nm range in the solid state (Figure 3c), whereas the An2Py luminophores exhibited no CD signals (Figure 3a), indicative of effective chirality transfer under crystalline conditions. The CD signals observed for **3a**, **3b**, **4a**, and **4b** in the 350–530 nm regions are ascribable to through‐space chirality transfer from the chiral silver(I) cluster to the originally achiral luminescent ligand (An2Py) when the excitation spectrum of An2Py is considered. In addition, the CD responses of the An2Py molecules are related to core‐to‐ligand and ligand‐to‐ligand chirality transfer due to the existence of multiple intermolecular CSA^−^/An2Py interactions (Figures 2e,f).^[^ ^27^ ^]^ It can be observed in the CD spectra of **3** that there is a couple of extended exciton‐type Cotton effects, which consists of two bands of opposite sign and similar amplitude whose crossover point occurs near 370 nm, unlike the totally negative band for **4a**. Such behavior corresponds to their structure. The formed chiral An2Py exists in a quasi‐parallel assembly chromophores in **3** (Figure 2e) while is isolated in **4**.^[^ ^28^ ^]^

**Figure 4:**
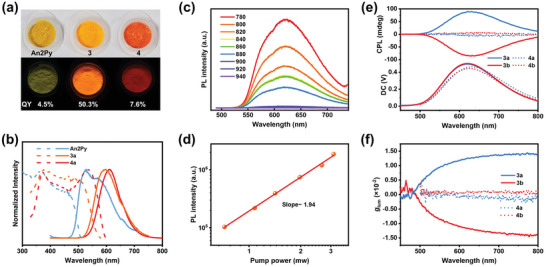
a) Photographic images of An2Py, **3a**, and **4a** under ambient and UV light. b) Normalized excitation (dotted traces) and emission (solid traces) spectra of An2Py, **3a**, and **4a** at room temperature when irradiated with 365, 460, and 370 nm light. c) Two‐photon emission spectra of **3a** at various excitation wavelengths with femtosecond pulsed laser excitation. d) Two photoexcitation photoluminescence of **3a** following excitation at 800 nm as a function of power. e) CPL and fluorescence spectra and f) *g*_lum_ spectra of **3a, 3b, 4a**, and **4b**.

The photoluminescence spectra in **Figure** **4** show fluorescence (FL) emission bands that range from 460 to 710 nm (*λ*_Em_ = 590 nm, *τ* = 4.3 ns) for the An2Py ligand in the solid state. **3a** exhibit an orange FL emission (*λ*_Em_ = 612 nm, *τ* = 21.2 ns) in the crystalline state at room temperature when irradiated with 460 nm light, which is tentatively assigned to ligand‐centered emission states according to DFT calculations (Figure 4b; Figure S13, Supporting Information). The bathochromic shift in the emission of **3a** compared to that of free ligand is possibly due to increases in exciton coupling and orbital overlap between neighboring molecules in the framework.^[^ ^29^ ^]^ In addition, *π*···*π* stacking interactions need to be considered for the redshifted fluorescence and the slightly lower fluorescence quantum yield (An2Py in CHCl_3_: 53.2%; **3a**: 50.3%), in which the anthracene planes in two adjacent An2Py molecules overlap in almost a face‐to‐face stacking arrangement with a centroid‐to‐centroid distance of 3.81 Å (Figures S6 and S14, Supporting Information). The emission from **4a** shows a bathochromic shift to 620 nm and a shorter lifetime (*τ* = 0.85 ns, Figure S15, Supporting Information), which is ascribable to the protonation of An2Py.^[^ ^30^ ^]^ As shown in Figure S16 (Supporting Information), the observed decrease in fluorescence intensity with increasing water fraction indicates that An2Py has ACQ properties. While ACQ molecules are only suitable for use in dilute solutions, large‐scale clusters greatly separate An2Py units among adjacent molecules to reduce the weak interaction region, thereby facilitating a higher QY (An2Py in solid: 4.5%; **4a**: 7.6%) and broadening the application scope. **3a** exhibit a higher QY and a longer nanosecond lifetime compared to powdered An2Py (QY: 4.5%) (Figures S14 and S15, Supporting Information), consistent with the higher rigidity sustained by the assembled materials that minimize the nonradiative decay rate of the ligand, in agreement with the smaller nonradiative deactivation rate constant (*k_n_*_r_) of Ag_12_An2Py (2.35 × 10^7^ s^−1^) compared to that of An2Py (2.22 × 10^8^ s^−1^; Table S1, Supporting Information). Notably, the QY of **3a** is six‐time that of **4a** after fixation with coordination bonds, which highlights that the cluster assembly strategy is an effective way for improving luminescence. In addition, **3a** exhibit almost identical fluorescence intensity at 620 nm and PXRD patterns before and after being exposed to UV light for 100 min, indicative of good photostability (Figures S17 and S18, Supporting Information).

Two‐photon excited fluorescence (TPEF) is a process in which two photons are absorbed simultaneously and a photon with a frequency greater than that of the absorbed photons is emitted after strong interactions with short laser pulses. We investigated the TPEF of **3a** (Figure 4c), which exhibited two‐photon‐excited fluorescence when illuminated with near‐infrared femtosecond laser pulses. The slight differences observed between the emission peaks are probably ascribable to reabsorption and scattering effects of the solid sample during the collection process. In addition, we examined the relationship between emission intensity and excitation power intensity, which yielded a slope close to two (Figure 4d), consistent with a two‐photon process.^[^ ^31^ ^]^

CPL is related to the excited‐state properties of the chiral emitter. Hence, the excited‐state chirality of **3a**, **3b**, **4a**, and **4b** were examined by investigating their CPL properties. CPL spectra were collected by rotating the sample to eliminate the contribution of linear polarity. Negative and positive CPL emission signals were observed for **3a** and **3b** in the same wavelength region. In contrast, **4a** and **4b** only showed one‐ninth mirror‐image CPL intensity compared to those of **3a** and **3b** (Figure 4e). In addition, both **4a** and **4b** are CPL silent in methanol solution, although they exhibit CD signals and fluorescence, indicative of a lack of efficient chirality transfer between the chiral groups and luminescent moieties (Figure S19, Supporting Information). Furthermore, *g*_lum_, as an important indicator of CPL activity, is measured to be approximately ±1.3 × 10^−3^ for **4a** and **4b** at 630 nm, which are comparable to those of previously reported clusters. It is worth mentioning that **3a** exhibited a |*g*_lum_| up to 0.012 (Figure 4f), which is attributable to confinement by the coordination assembly and the close packing of the frame.^[^ ^26^ ^]^

### 2.3 Theoretical Calculations

**Figure 5:**
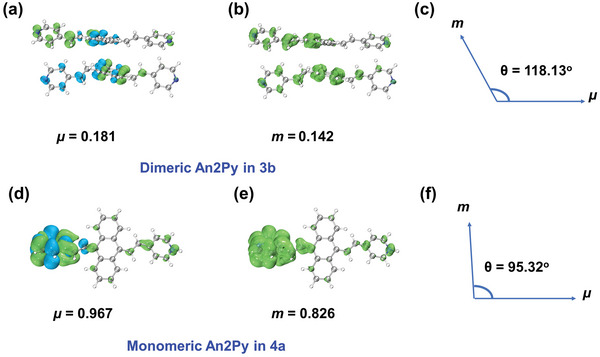
Calculated transition electric dipole moments (*µ*), magnetic dipole moments (*m*), and the angles (*θ*) between them in dimeric An2Py in **3b** (a‐c) and monomeric An2Py in **4a** (d‐f).

To uncover the reason for the dramatically higher *g*_lum_ exhibited by the coordination assembly **3b** relative to that observed for the cocrystalline system **4a**, we calculated the transition electric dipole moment (*µ*), the transition magnetic dipole moment (*m*), and the angle (*θ*) between them, which together determined *g*_lum_.^[^ ^32^ ^]^ Considering the emissions from the An2Py moieties in **3b** and **4a** and their assembly characteristics, An2Py exists in a quasi‐parallel assembly in **3b** (Figure 2e) but is isolated in **4a** (Figures 2d,f). We selected two calculational models based on the single‐crystal structure: an An2Py dimer (**Figure** **5**a,b) and an An2Py monomer (Figure 5d,e). Significantly lower *µ* and *m* values were determined for dimeric An2Py in **3b** compared to monomeric An2Py in **4a**, while *θ* was calculated to be 118.13° in the former (Figure 5c) and 95.32° in the latter (Figure 5f). Consequently, **3b** has a *g*_lum_ value that is ≈4 times greater than **4a**. The optimized structure for each ground state (S_0_) and first singlet excited state (S_1_), and the components of *µ* and *m* in the *x*, *y*, and *z* directions are shown in Figures S20–S22 and Table S2 (Supporting Information). The data reveal that assembling highly emissive organic molecules in a favorable stacking arrangement in a chiral crystal field may be an efficient method for obtaining large *g*_lum_ values. The details of the mechanism responsible for this observation will be investigated in future studies.

### 2.4 Optical Switch

**Figure 6:**
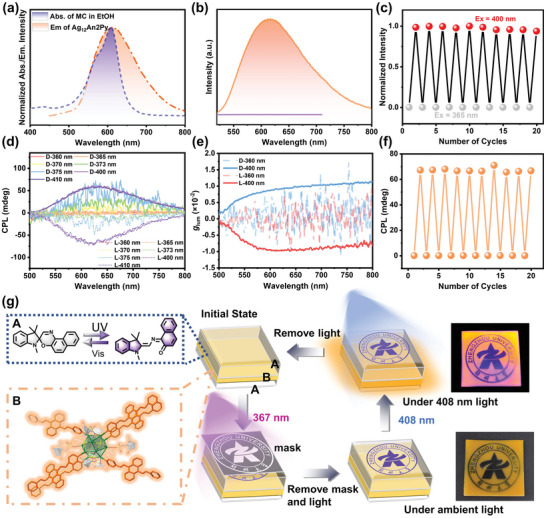
a) The absorption spectrum of MC (blue) in EtOH overlaps largely with the emission spectrum of **3a** (red). b) Emission spectra of **3a** in MMM under UV or visible light (purple and red traces, respectively). c) Visualizing the switching cycle. The emission intensity of excited **3a** is affected greatly by excitation wavelength. d) CPL and e) *g*_lum_ spectra of **3** when excited at different wavelengths. f) CPL photoswitching of **3a** monitored at 625 nm as the light is alternated between UV (360 nm) and visible (400 nm). g) Luminescence is switched by the action of UV and visible light.

This type of luminescent material, which is very stable and exhibits a CPL signal with a high *g*_lum_ value, prompts us to explore its use in smart stimuli‐responsive optical materials, which is vital for addressing emerging demands. Spirooxazine (SO) and merocyanine (MC) are reversibly interconverted through ring isomerization triggered by switching between visible light and UV light (Figure S23, Supporting Information). Overlap between the absorption bands of the MC and a CMOF results in fluorescence emission or quenching under alternating visible and UV‐light conditions (**Figure** **6**a,b). A strategy for designing optical switches was adopted by encapsulating photochromic molecules and a chiral luminescent MOF into a PDMS carrier unit to construct an MMM (Figure 6g). Based on the photochromatic SO‐switching process, a toluene solution of SO was mixed with liquid PDMS and a curing agent, the mixture was drop‐coated, and the first and third layers were solidified. Crystals of **3a**, which were regarded as the emission units, located in the second layer of the MMM and coated completely with SO‐doped PDMS. Photoswitching cycles involving alternating UV and visible light further demonstrated the effectiveness of the MMM‐based optical switch (Figure 6c). The photoresponsive CPL performance of the MMM when irradiated by the two light sources was further explored. Figure 6d,e shows that the MMM exhibits mirror‐image CPL signals that are switched between active and silent states with obvious spectral variations when excited at 400 and 360 nm, along with isomerization from the closed‐ring to the open‐ring form of the photochromic molecule. The symmetrical CPL signals remain unchanged when the excitation wavelength changed from 370 to 410 nm, except for a slight difference in CPL‐response intensity (Figure 6d). The *g*_lum_ value of the CPL emission did not significantly depend on the excitation wavelength (Figure S24, Supporting Information). Similarly, such cycling was repeated at least ten times (Figure 6f). Furthermore, reversible photoswitching with photopatterning was explored using the MMM. The MMM was locally irradiated through a photomask with 367 nm light (Figure 6g). The light‐filtered part of the mask generated the school emblem, and the MMM did not luminesce during the entire process. The light source was quickly switched to 408 nm after removing the mask, and all parts except for the school emblem emitted orange luminescence. This technique provides opportunities for designing chiroptical switches.

## 3 Conclusion

In conclusion, chiral metal cluster‐based reticular materials with strong CPL emissions were constructed for the first time through hierarchical coassembly. The chirality of cluster with no luminescence was transferred to the anthracene‐containing ACQ emitter by two approaches, ion‐pair‐direct assembly and coordination assembly, triggering bright CPL. More importantly, the anthracene‐type linker packed in pairs and quasi‐parallel in the chiral reticular nets achieves a superb *g*_lum_ of 1.2 × 10^−2^ and PLQY of 50.3%, further highlighting the merits of directional arrangement in reticular nets. We also fabricated a UV–vis‐controlling CPL switches using this cluster‐based reticular framework. The current study extends a new approach to assemble high‐performance CPL material, and provides an atomic‐level understanding of the relationship between chirality transfers and amplification and CPL activity and the structure of chiral assembly.

[CCDC 2216751–2216755 and 2216757–2216759 contain the supplementary crystallographic data for this paper. These data can be obtained free of charge from The Cambridge Crystallographic Data Centre via www.ccdc.cam.ac.uk/data_request/cif.]

## Conflict of Interest

The authors declare no conflict of interest.

## Supporting information

Supporting Information

Supporting Information

## Data Availability

The data that support the findings of this study are available in the supplementary material of this article.
