# Supplementary material for: Stepwise Amplification of Circularly Polarized Luminescence in Chiral Metal Cluster Ensembles
Source: Adv Sci (Weinh). 2023 Feb 25;10(13):2207660. doi: 10.1002/advs.202207660 (PMC10161016; doi:10.1002/advs.202207660)
Supplement: Supplementary file 1 — Supporting Information [file ADVS-10-2207660-s001.pdf]

ADVANCED SCIENCE
Open Access

Supporting Information

for *Adv. Sci.*, DOI 10.1002/advs.202207660

Stepwise Amplification of Circularly Polarized Luminescence in Chiral Metal Cluster Ensembles

Jia-Yin Wang, Yubing Si, Xi-Ming Luo, Zhao-Yang Wang, Xi-Yan Dong, Peng Luo, Chong Zhang, Chunying Duan* and Shuang-Quan Zang**

Supporting Information

Stepwise Amplification of Circularly Polarized Luminescence in Chiral Metal Cluster Ensembles

Jia-Yin Wang,[‡] Yubing Si,[‡] Xi-Ming Luo, Zhao-Yang Wang, Xi-Yan Dong, Peng Luo, Chong Zhang, Chunying Duan,* and Shuang-Quan Zang**

Experimental Section

Materials and Reagents.

All reagents and solvents were commercially available and used without any additional purification, $S^{i}PrAg$ was prepared from the reaction of molar equivalents of Ag_2O and 2-Propanethiol in Et_3N. CSA-Ag, prepared from the reaction between silver carbonate and D/L-camphorsulfonic acid, was obtained after stirring for several hours in the dark. After the reaction, the mixture was filtered and the filtrate was evaporated to dryness. Other reagents and solvents for synthesis were obtained from commercial sources and used without further purification. All solvents were analytical grade reagent.

Characterization.

Powder X-ray diffraction (PXRD) patterns of the samples were recorded at room temperature in air using a Rigaku MiniFlex diffractometer (Cu-Kα; λ = 1.54178 Å; 2θ range of 3-50°). Elemental analysis of C, H, and N were performed on a Perkin-Elmer 2400 elemental analyzer. The thermogravimetric analyses of the as-synthesized nanoclusters was performed on a SDT 2960 thermal analyzer from room temperature to 600 °C at a heating rate of 10 °C/min under N_2 atmosphere. Fourier transform infrared (FT-IR) spectra were recorded in the 400-4000 cm^{-1} range on a Bruker TENSOR 27 FT-IR spectrometer with pressed KBr pellets.

Crystallographic data collection and structural refinement.

SCXRD measurements were performed on a Rigaku XtaLAB Pro diffractometer with Cu-Kα radiation (λ =1.54184 Å) at 150 K for **2b** and at 200 K for **1a**, **1b**, **2a**, **3a**, **3b**, **4a**, and **4b**. Data collection and reduction were performed using the program CrysAlisPro.[1] The structures were solved with intrinsic phasing methods (*SHELXT-2015*)[2] and refined by full-matrix least squares on F^2 using *OLEX2*, which utilizes the *SHELXL-2015* module.[3] All the atoms were refined anisotropically. Hydrogen atoms were placed in calculated positions refined using idealized geometries and assigned fixed isotropic displacement parameters. Intrinsic disorder occurred in structures of **2a**, **2b**, **3a**, **3b**, **4a**, and **4b**, although all X-ray intensity data displayed reasonably good quality as reflected by their low R_{int} (0.0381-0.1167) and R_{sigma} (0.0453-0.088) values. In addition, the Flack parameters of 0.190 (9) for **3b** are higher than those of other complexes and partly D-H bond can't find acceptor, which is mainly due to the structural disorder of CSA−. Structure refinement was handled with different strategies according to the electron density distribution and the complexity of the disorder. The imposed

the restraints and constraints (ISOR, SIMU, DELU, SADI, DANG, etc.) in least-squares refinement of each structure were commented in the corresponding crystallographic CIF files. Thus, only a general description of the structural refinement strategy is presented here. The crystal structures are visualized by DIAMOND 3.2.[4] The detailed information of the crystal data, data collection and refinement results for all compounds are summarized in Table S3-S6.

Photophysical measurements.
UV-Vis diffuse reflectance spectra were measured using a Hitachi UH4150 UV-Vis-NIR spectrophotometer, with $BaSO_4$ as a reference, and steady-state photoluminescence (PL) spectra of samples were recorded on a HORIBA FluoroLog-3 fluorescence spectrometer using a xenon lamp as the excitation source. The fluorescence lifetime was determined using a HORIBA FluoroLog-3 fluorescence spectrometer equipped with 370 nm and 420 nm laser operating in time-correlated single photon counting mode (TCSPC). Circular dichroism (CD) spectra were obtained by a JASCO J-1500 spectropolarimeter in solid state or membrane. Each CD spectrum was the average of at least three scans. Circularly polarized luminescence (CPL) spectra were strictly obtained on a JASCO CPL-300. In process, the ground solid-state samples (a small amount) were uniformly sandwiched by two quartz slides to test. Certainly, the instrument was carefully calibrated to eliminate influence of other factors and the reproducibility of CPL spectrum for each sample was confirmed by measuring at least three times. CD and CPL spectra were collect by rotating the sample to eliminate the contribution of linear polarity. The value of g_{lum} was defied as $g_{\text{lum}} = 2 \times$ [ellipticity/(32980/ln10)]/total florescence intensity at the CPL extremum. The samples were fixed on the instrument directly and perpendicular to the light beam during the CD and CPL measurement.

Calculation methods.
The calculations were performed using the semiempirical quantum mechanical methods GFN1-xTB packages.[5-7] The single crystal structure was chosen as initial guess for ground state optimization at tight level. The optimized structure preserved the basic characteristics of the input structure, only with slightly change in the bond length, bond angles and dihedral angles, which confirming the feasibility of GFN1-xTB methods for cluster calculations. The Hirshfeld charge was conducted by Multiwfn 3.8.[8] To simulate the dissymmetry factor of ligand molecules, the ground states of An2Py in **3b** and **4a** were first optimized at the level of PBE0/6-31G(d,p), the harmonic frequency analysis were further performed to make the structures in the local minima. Then the excited states were optimized at the same theoretical level based on ground states. All the density functional theory (DFT) and time-dependent density functional theory (TDDFT) calculations were performed by Gaussian 16 program.[9] The transition electric dipole moments and transition magnetic dipole moments, as well as the molecular orbitals were generated and visualized by VMD and Multiwfn programs.[10, 11]

Experimental Section
*Preparation of **1a/1b***: $S^{i}PrAg$ (0.015 g, 0.08 mmol) and D/L-CSA-Ag (0.025 g, 0.08 mmol) were dissolved in a mixed solvent of DCM and MeOH (1:2, 6 mL) under stirring for 3 min at RT. The resultant solution was allowed to evaporate slowly in darkness at room temperature for three days to give colorless block crystals. The colorless block crystals were filtered off, washed with MeOH and dried at room temperature. Yields: 41% for **1a** and 47% for **1b** based on Ag. Elemental analysis calcd. (%) for **1** ($C_{82}H_{148}Ag_{12}O_{28}S_{12}$) C, 30.20; H, 4.57; S, 11.80. Found: C, 30.01; H, 4.47; S,11.54.

*Preparation of **2a/2b***: $S^{i}PrAg$ (0.015 g, 0.08 mmol) and D/L-CSA-Ag (0.025 g, 0.08 mmol) were dissolved in a mixed solvent of DCM and MeOH (1:2, 6 mL) under stirring for 3 min at

RT. Then, pyridine (0.5 mL, 6.3 mmol) was added to the colorless clear solution. The resultant solution was allowed to evaporate slowly in darkness at room temperature for one week to give colorless block crystals. The colorless block crystals were filtered off, washed with diethyl ether and dried at room temperature. Yields: 26% for **2a** and 31% for **2b** based on Ag. Calcd for **2a**, $[Ag_{12}(S^{i}Pr)_6(D/L\text{-}CSA)_6(py)_7(H_2O)][Ag_{12}(S^{i}Pr)_6(D/L\text{-}CSA)_6(py)_8]\cdot(py)_3(H_2O)_5$, C, 37.89; H, 4.73; N, 3.23; S, 9.87. Found: C, 37.65; H, 4.48; N, 3.14; S, 9.58. Calcd for **2b**, $[Ag_{12}(S^{i}Pr)_6(D/L\text{-}CSA)_6(py)_7(H_2O)][Ag_{12}(S^{i}Pr)_6(D/L\text{-}CSA)_6(py)_8]\cdot(py)_3(H_2O)_6$, C, 37.80; H, 4.75; N, 3.23; S, 9.84. Found: C, 37.71; H, 4.49; N, 3.14; S, 9.55.

*Preparation of **3a/3b***: $S^{i}PrAg$ (0.015 g, 0.08 mmol) and D/L-CSA-Ag (0.025 g, 0.08 mmol) were dissolved in a mixed solvent of DCM and MeOH (1:2, 6 mL) under stirring for 3 min at RT. Then, An2Py (5 mg, 0.01 mmol) was added to the colorless clear solution. The resultant solution was allowed to evaporate slowly in darkness at room temperature for three days to give orange block crystals. The orange block crystals were filtered off, washed with MeOH and dried at room temperature. Yields: 23% for **3a** and 25% for **3b** based on An2Py. Elemental analysis calcd. (%) for **3** ($C_{162}H_{196}Ag_{12}N_6O_{26}S_{12}$): C 45.02; H 4.57; N 1.94; S 8.90; found: C 44.58; H 4.36; N 1.65; S 8.70.

*Preparation of **4a/4b***: $S^{i}PrAg$ (0.009 g, 0.05 mmol) and D/L-CSA-Ag (0.025 g, 0.08 mmol) were dissolved in a mixed solvent of chloroform and acetone (1:1, 6 mL) under stirring for 3 min at RT. Then, An2Py (2 mg, 0.005 mmol) was added to the colorless clear solution. The resultant solution was allowed to evaporate slowly in darkness at room temperature for few hours to give orange plate crystals. The orange plate crystals were filtered off, washed with diethyl ether and dried at room temperature. Yields: 20% for **4a** and 23% for **4b** based on An2Py. Calcd for evacuated **4a** ($C_{126}H_{184}Ag_{12}N_2O_{32}S_{14}$): C, 38.00; H, 4.66; N, 0.70; S, 11.27. Found: C, 37.55; H, 4.53; N, 0.63; S, 10.97. Calcd for evacuated **4b** ($C_{126}H_{186}Ag_{12}N_2O_{33}S_{14}$): C, 37.83; H, 4.69; N, 0.70; S, 11.22 Found: C, 37.51; H, 4.48; N, 0.64; S, 11.03.

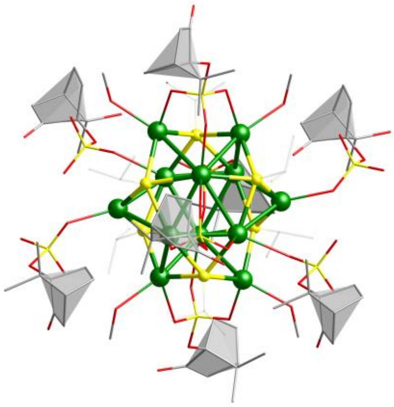

Figure S1. Perspective view of the coordination environment of Ag_{12} cluster in **1a**.

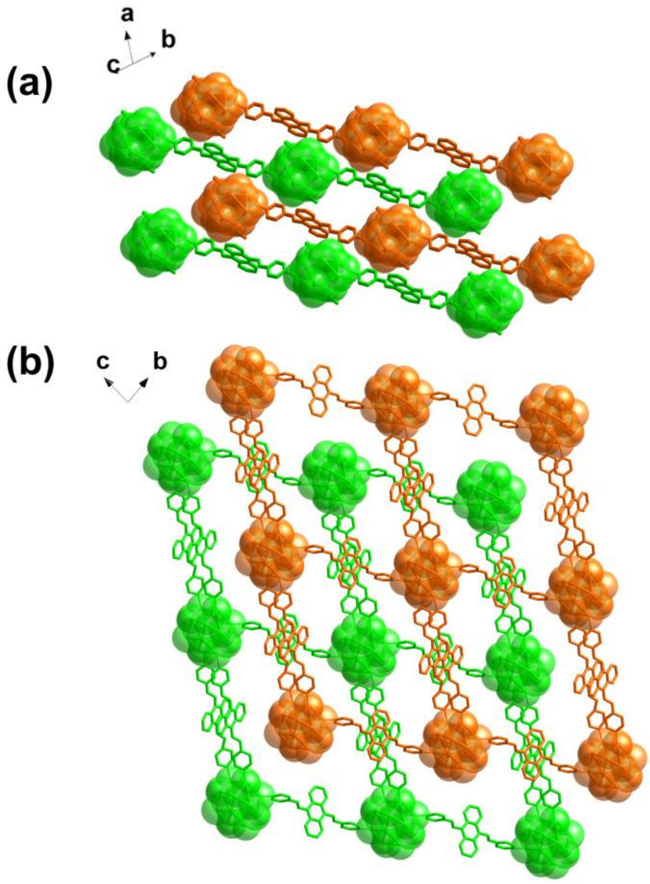

Figure S2. Stacking of the 2D network structure of **3a** viewed along different directions.

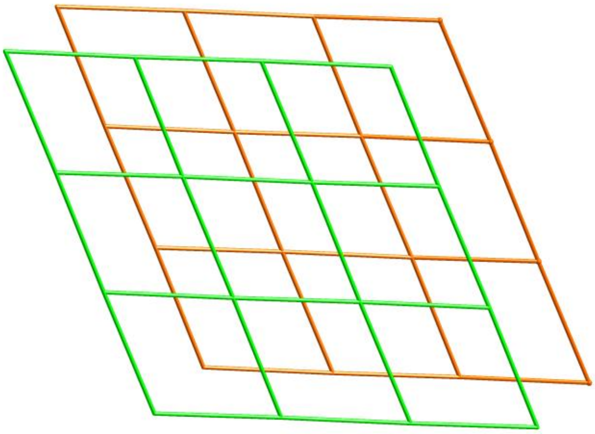

Figure S3. Schematic representation of the topology of **3a** along the *a*-axis. The square windows in one layer are blocked by Ag–S cluster nodes of adjacent layers.

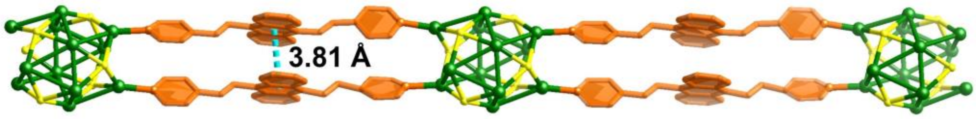

Figure S4. The π···π distance between the anthryl moieties in the **3a** crystal structure.

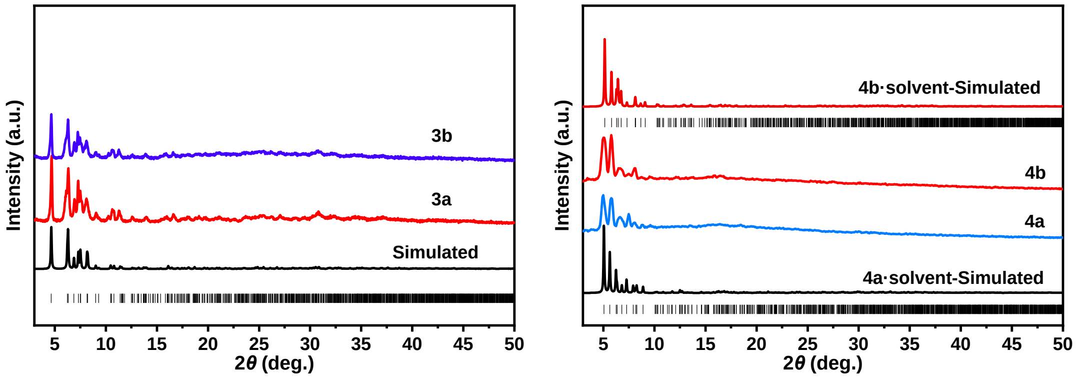

Figure S5. X-Ray powder diffraction (PXRD) patterns of **3** and **4·solvent** simulated from single-crystal data (black line) and the experimental PXRD patterns for complexes **3a**, **3b**, **4a** and **4b** after air-drying.

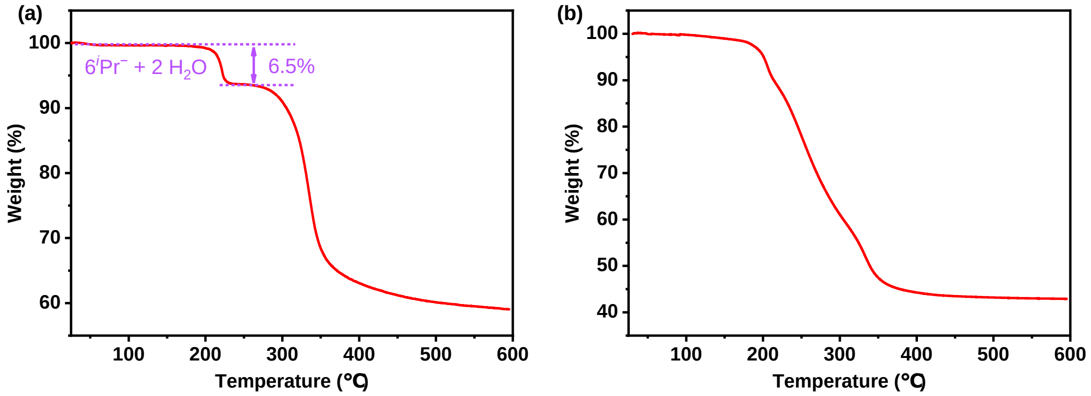

Figure S6. Thermogravimetric analysis of (a) **3a** and (b) **4a** under N_2 atmosphere (10 °C /min). The weight lost (6.5%) between 180–250 °C may be corresponding to the dissociation of $-^iPr$ and H_2O (the calculated 6.8%).

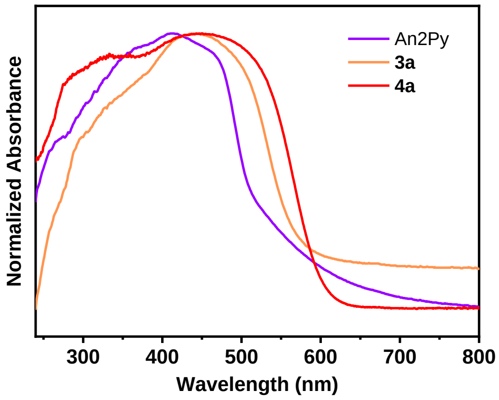

Figure S7. Normalized UV-vis absorption spectra of An2Py, **3a** and **4a**.

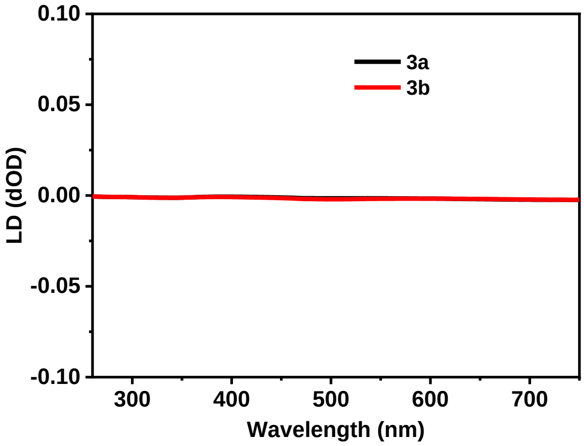

Figure S8. LD spectra of **3a** and **3b** in the film state.

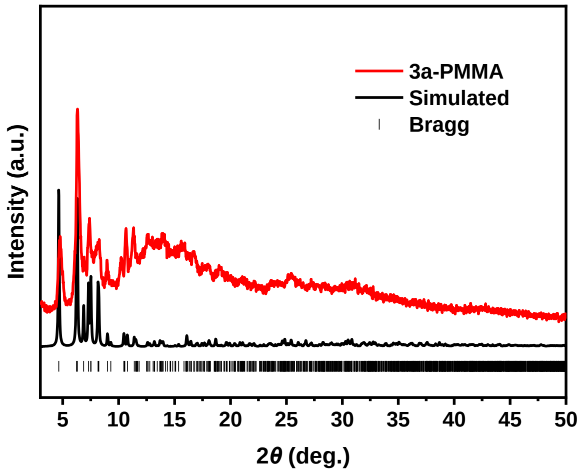

Figure S9. The simulated and polymer-embedded-state PXRD patterns for complexes **3a**.

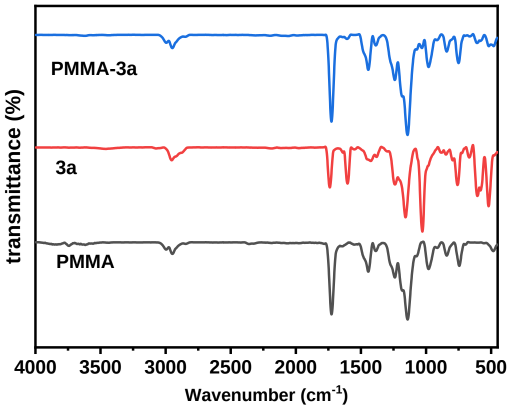

Figure S10. FT-IR spectra of PMMA, **3a** and polymer-embedded-state **3a**.

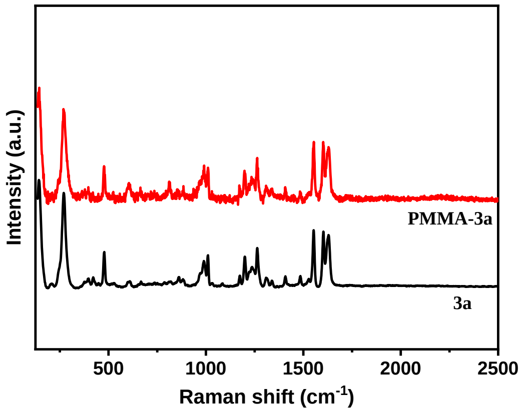

Figure S11. Raman spectra of **3a** and polymer-embedded-state **3a**.

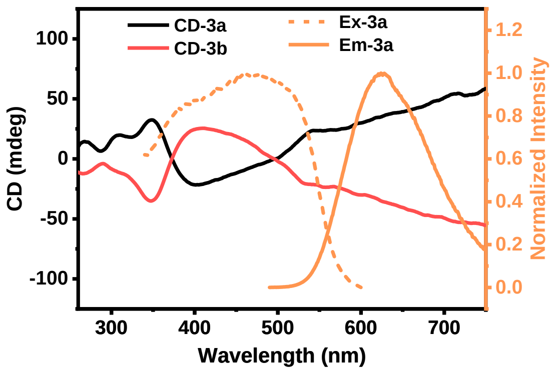

Figure S12. The CD spectra of **3** and the normalized excitation (dotted trace) and emission spectra of **3a** at room temperature.

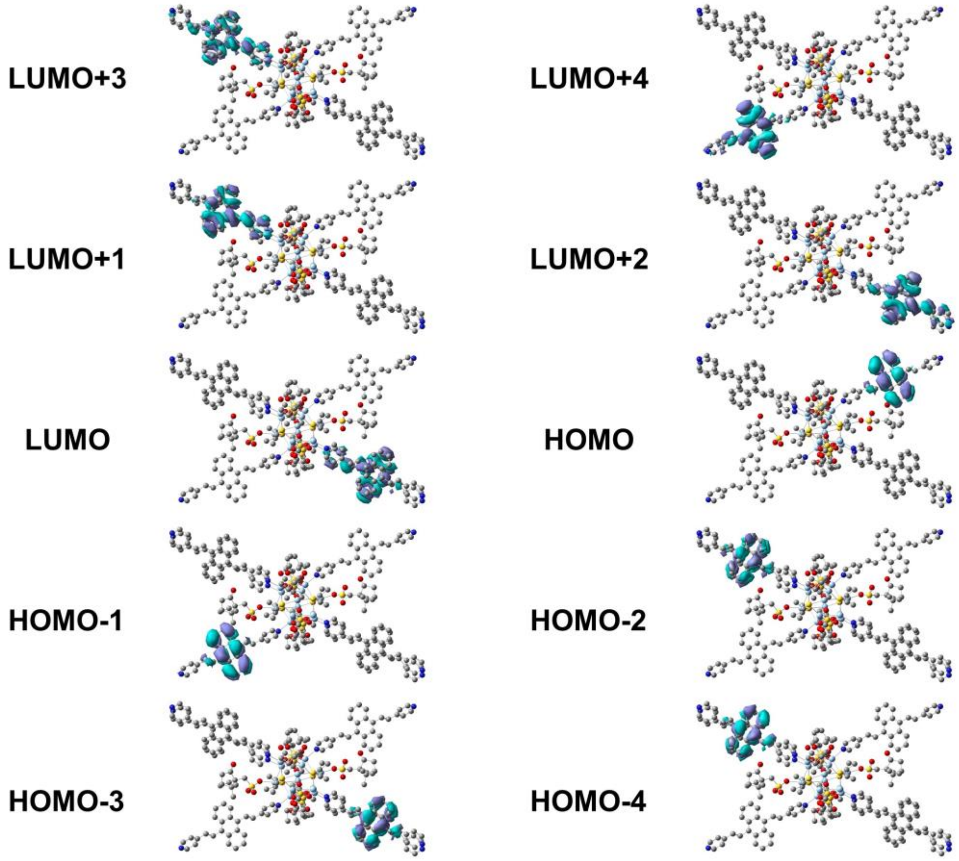

Figure S13. Selected frontier molecular orbitals representations for **3a**.

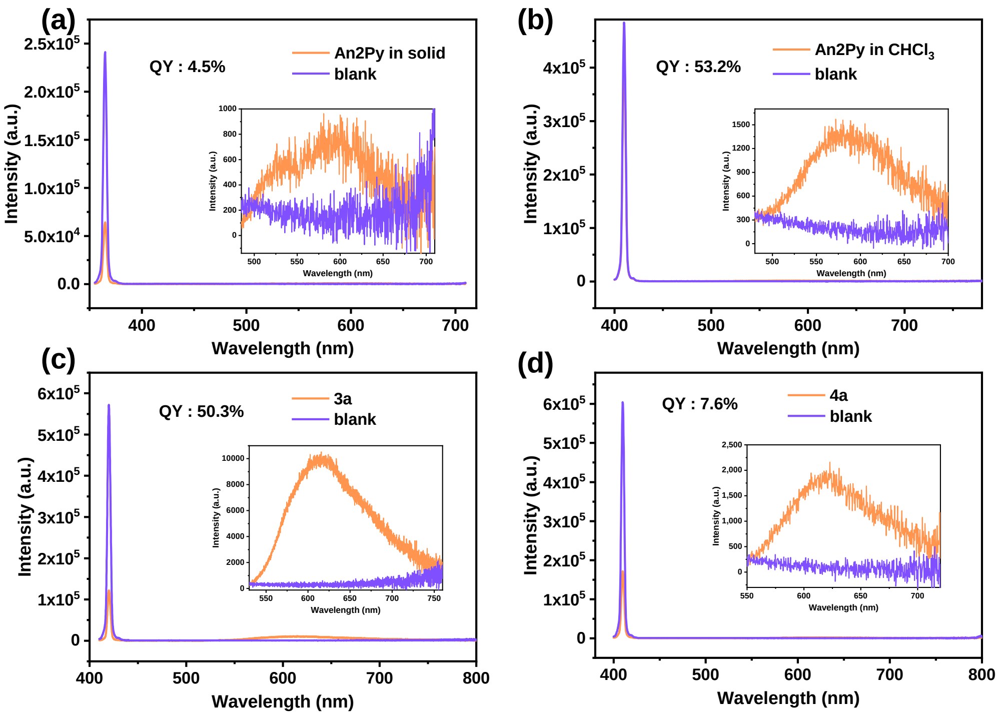

Figure S14. Quantum yield of (a) An2Py in solid, (b) An2Py in $CHCl_3$, (c) **3a** and (d) **4a**.

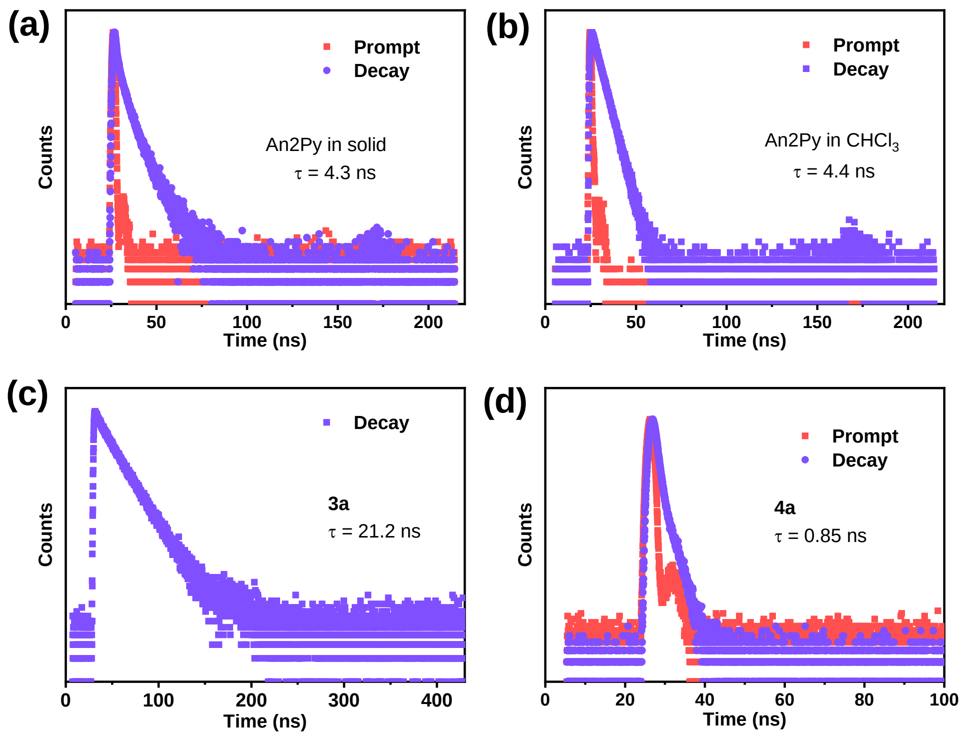

Figure S15. Luminescence decays of (a) An2Py at 590 nm in solid after excitation at 370 nm; (b) An2Py at 580 nm in the chloroform solution ($c = 10^{-5}$ M) after excitation at 420 nm; (c) **3a** at 612 nm in the solid state after excitation at 370 nm (d) **4a** at 620 nm after excitation at 370 nm.

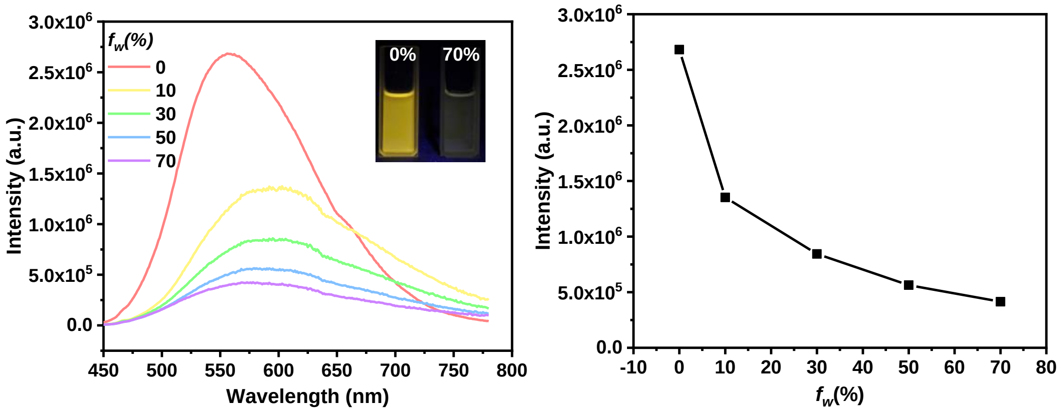

Figure S16. Fluorescence emission spectra of 10^{-5} mol L^{-1} An2Py in THF/H_2O with different water fractions. Inset: Images of An2Py in tetrahydrofuran (THF, 1×10^{-5} M) with 0% and 70% of H_2O under UV light.

Table S1. Photophysical properties of crystals.

Sample	□em (nm)	□Φ	τ (ns)	K_r (ns^{-1})	K_{nr} (ns^{-1})
**An2Py in solid**	590	0.045	4.3	0.011	0.222
**An2Py in $CHCl_3$**	580	0.532	4.4	0.121	0.106
**3a**	612	0.503	21.2	0.024	0.024
**4a**	620	0.076	0.85	0.089	1.087

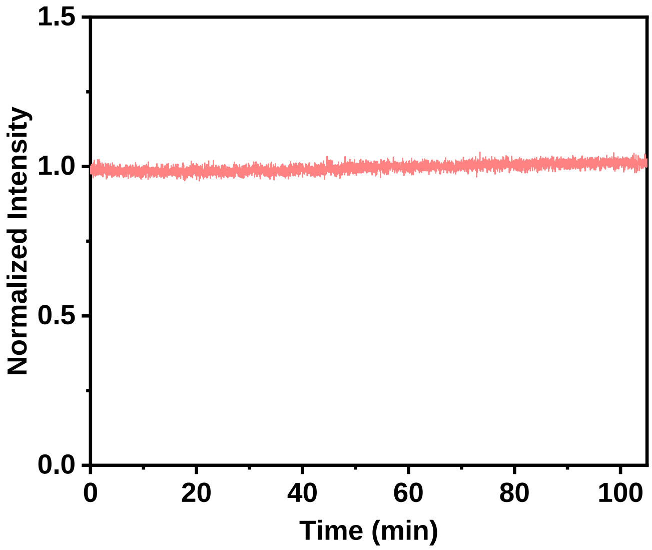

Figure S17. Time-dependent fluorescence spectra of **3a** at room temperature. Excitation and emission wavelengths were 365 nm and 612 nm, respectively.

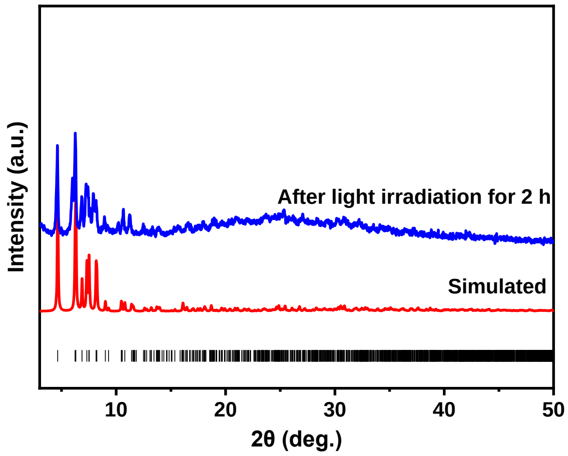

Figure S18. The simulated and experimental PXRD patterns for complexes **3a** after 2 h of visible-light irradiation under a Xe lamp equipped with 420 nm cutoff filter.

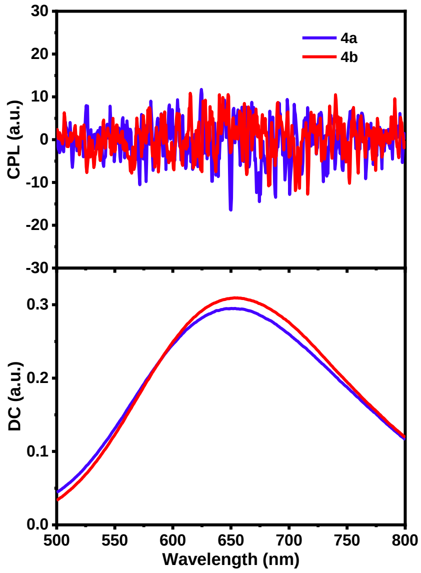

Figure S19. CPL and DC spectra of **4** in MeOH.

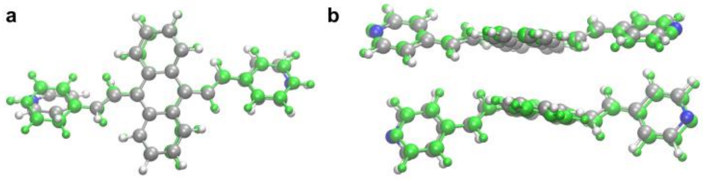

Figure S20. Coordinative alignment of the ligand molecules (a) **4a** (b) **3b**. The ground (S0) and excited states (S1) are colored by the gray and green respectively.

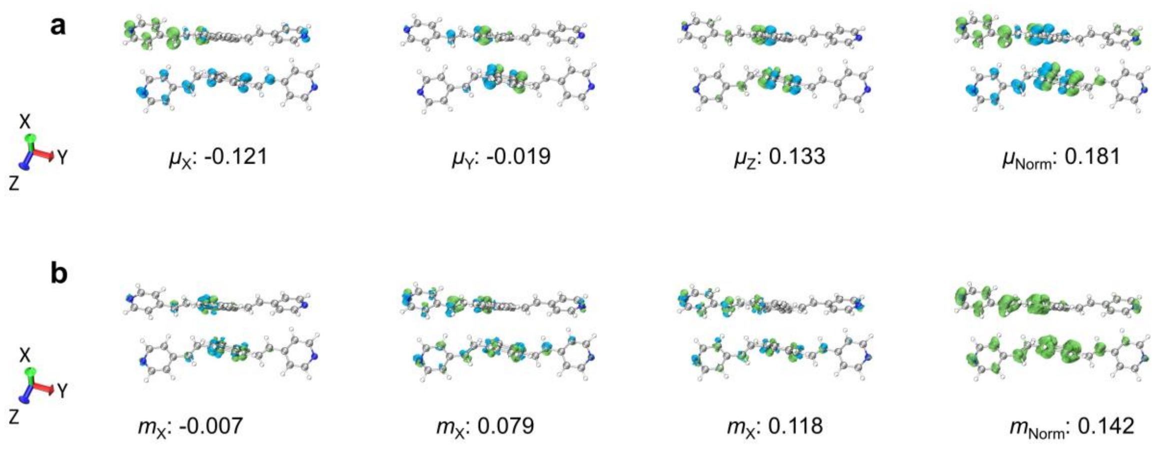

Figure S21. (a) The electric transition dipole moments analysis of An2Py in **3b**, the attachment and detachment density are shown in green and blue parts respectively (isovalue=0.002); (b) The magnetic transition dipole moments analysis of An2Py in **3b**, the attachment and detachment density are shown in green and blue parts respectively (isovalue=0.002).

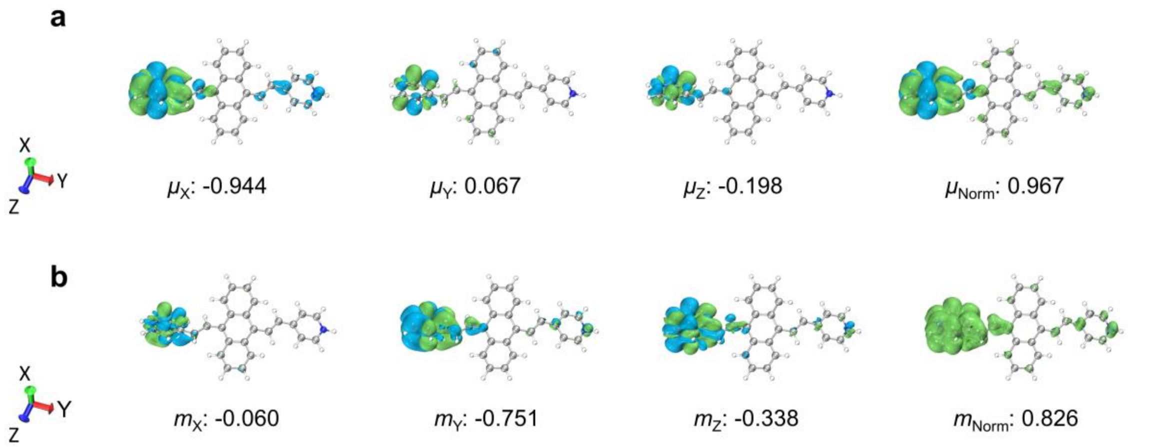

Figure S22. (a) The electric transition dipole moments analysis of An2Py in **4a**, the attachment and detachment density are shown in green and blue parts respectively (isovalue=0.002); (b) The magnetic transition dipole moments analysis of An2Py in **4a**, the attachment and detachment density are shown in green and blue parts respectively (isovalue=0.002).

Table S2. The length gauge of transition electric dipole moments (μ), transition magnetic dipole moments (m), dipole strength (D), rotatory strength (R), angle between μ and m (θ), dissymmetry factor of circularly polarized luminescence (g_{lum}) for An2Py in **3b** and**4a**.

**Sample**	$\lvert\mu\rvert$ (10^{-20} esu cm)	$\lvert m\rvert$ (10^{-20} erg G^{-1})	D (10^{-40} esu^2 cm^2)	R (10^{-40} esu cm erg G^{-1})	$\theta_{\mu,m}$ (deg)	cos $\theta_{\mu,m}$	Theoretical g_{lum}
**3b**	29.17953608	0.139834446	851.445326	-1.92E+00	118.1348685	-0.472	-9.04E-03
**4a**	145.6555621	0.917699655	21215.54278	-12.39	95.31896725	-0.093	-2.34E-03

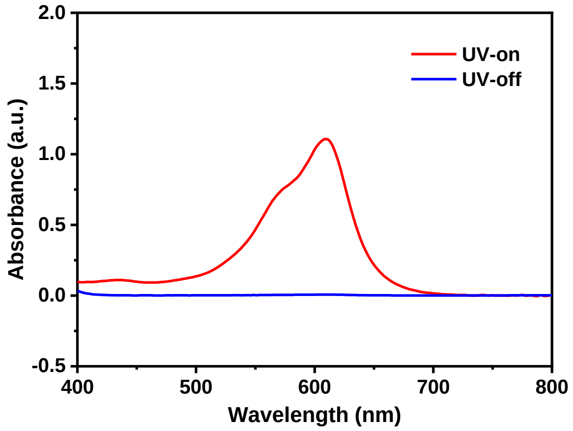

Figure S23. The absorption spectra of SO/MC under the switchable UV light irradiation.

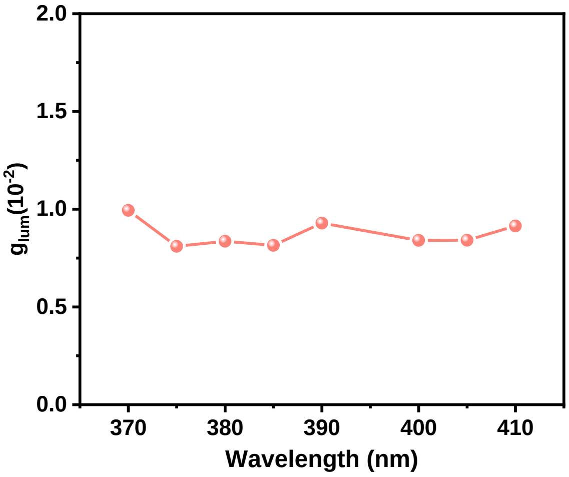

Figure S24. Corresponding g_{lum} values of **3a** at different excitation wavelength.

Table S3. Crystal data and structure refinement for **1a** and **1b**.

	**1a**	**1b**
**CCDC number**	2216751	2216752
**Empirical formula**	$C_{82}H_{148}Ag_{12}O_{28}S_{12}$	$C_{82}H_{148}Ag_{12}O_{28}S_{12}$
**Formula weight**	3261.16	3261.16
**Temperature / K**	200	200
**Crystal system**	triclinic	triclinic
**Space group**	*P*1	*P*1
***a* / Å**	11.7133(1)	11.7185(1)
***b* / Å**	13.8082(1)	13.8017(2)
***c* / Å**	18.2807(2)	18.2811(3)
**α / °**	84.853(1)	84.850(1)
**β / °**	71.513(1)	71.478(1)
**γ / °**	84.766(1)	84.784(1)
**Volume / $Å^3$**	2786.65(5)	2786.13(7)
**Z**	1	1
**ρ_{calc} g / cm^3**	1.943	1.944
**μ / mm^{-1}**	19.164	19.167
**F(000)**	1620.0	1620.0
**Crystal size / mm^3**	0.5 × 0.2 × 0.2	0.5 × 0.2 × 0.18
**Radiation**	Cu $K\alpha$ (λ = 1.54184)	Cu $K\alpha$ (λ = 1.54184)
**2θ range for data collection / °**	6.442 to 147.698	6.444 to 147.698
**Index ranges**	$-8 \le h \le 14$, $-16 \le k \le 17$, $-20 \le l \le 22$	$-14 \le h \le 14$, $-16 \le k \le 12$, $-22 \le l \le 22$
**Reflections collected**	22669	30108
**Independent reflections**	13947 [R_{int} = 0.0355, R_{sigma} = 0.0514]	14669 [R_{int} = 0.0574, R_{sigma} = 0.0663]
**Data / restraints / parameters**	13947 / 70 / 1247	14669 / 46 / 1247
**Goodness-of-fit on F^2**	1.011	1.016
**Final *R* indexes [I >= 2σ (I)]**	R_1 = 0.0362, wR_2 = 0.0924	R_1 = 0.0441, wR_2 = 0.1216
**Final *R* indexes [all data]**	R_1 = 0.0384, wR_2 = 0.0941	R_1 = 0.0475, wR_2 = 0.1245
**Largest diff. peak/hole / e $Å^{-3}$**	0.98 / -0.81	1.19 / -1.09
**Flack parameters**	0.010(9)	0.035(10)

Table S4. Crystal data and structure refinement for **2a** and **2b**.

	**2a**	**2b**
**CCDC number**	2216753	2216754
**Empirical formula**	$C_{246}H_{366}Ag_{24}N_{18}O_{54}S_{24}$	$C_{246}H_{368}Ag_{24}N_{18}O_{55}S_{24}$
**Formula weight**	7797.87	7815.88
**Temperature / K**	200	150
**Crystal system**	triclinic	triclinic
**Space group**	*P*1	*P*1
a **/ Å**	17.3281(2)	17.30140(11)
b **/ Å**	20.2709(2)	20.20659(12)
c **/ Å**	24.4284(2)	24.36782(13)
α **/ °**	100.583(1)	100.5975(5)
β **/ °**	107.534(1)	107.8152(5)
γ **/ °**	109.862(1)	109.6734(6)
**Volume / Å^3**	7293.38(14)	7235.41(8)
**Z**	1	1
ρ_{calc} **g / cm^3**	1.775	1.794
μ **/ mm^{-1}**	14.780	14.903
**F(000)**	3912.0	3922.0
**Crystal size / mm^3**	0.12 × 0.12 × 0.11	0.12 × 0.11 × 0.1
**Radiation**	Cu $K\alpha$ (λ = 1.54184)	Cu $K\alpha$ (λ = 1.54184)
**2θ range for data collection / °**	4.002 to 148.73	4.902 to 147.952
**Index ranges**	$-21 \le h \le 21$, $-25 \le k \le 25$, $-30 \le l \le 30$	$-21 \le h \le 21$, $-25 \le k \le 25$, $-30 \le l \le 30$
**Reflections collected**	179261	164177
**Independent reflections**	52972 [R_{int} = 0.0974, R_{sigma} = 0.0737]	52339 [R_{int} = 0.0946, R_{sigma} = 0.0707]
**Data / restraints / parameters**	52972 / 543 / 3379	52339 / 254 / 3388
**Goodness-of-fit on F^2**	1.059	1.019
**Final *R* indexes [I >= 2σ (I)]**	R_1 = 0.0582, wR_2 = 0.1594	R_1 = 0.0525, wR_2 = 0.1343
**Final *R* indexes [all data]**	R_1 = 0.0695, wR_2 = 0.1720	R_1 = 0.0561, wR_2 = 0.1363
**Largest diff. peak/hole / e Å^{-3}**	1.88 / -1.32	1.67 / -2.01
**Flack parameters**	0.020(5)	0.023(4)

Table S5. Crystal data and structure refinement for **3a** and **3b**.

	**3a**	**3b**
**CCDC number**	2216758	2216759
**Empirical formula**	$C_{162}H_{196}Ag_{12}N_6O_{26}S_{12}$	$C_{162}H_{196}Ag_{12}N_6O_{26}S_{12}$
**Formula weight**	4322.40	4322.40
**Temperature / K**	200	200
**Crystal system**	triclinic	triclinic
**Space group**	*P*1	*P*1
***a* / Å**	15.4455(1)	15.3536(3)
***b* / Å**	15.6831(1)	15.6214(3)
***c* / Å**	19.3575(2)	19.4032(3)
**α / °**	80.103(1)	80.377(1)
**β / °**	81.439(1)	81.929(1)
**γ / °**	65.512(1)	66.006(2)
**Volume / Å^3**	4187.96(7)	4178.22(15)
**Z**	1	1
**ρ_{calc} g / cm^3**	1.714	1.718
**μ / mm^{-1}**	12.934	12.964
**F(000)**	2174.0	2174.0
**Crystal size / mm^3**	0.28 × 0.15 × 0.05	0.3 × 0.15 × 0.05
**Radiation**	Cu $K\alpha$ (λ = 1.54184)	Cu $K\alpha$ (λ = 1.54184)
**2θ range for data collection / °**	6.312 to 147.508	4.634 to 150.51
**Index ranges**	$-17 \leq h \leq 19$, $-11 \leq k \leq 19$, $-24 \leq l \leq 23$	$-19 \leq h \leq 19$, $-19 \leq k \leq 19$, $-24 \leq l \leq 24$
**Reflections collected**	44670	102113
**Independent reflections**	20620 [R_{int} = 0.0381, R_{sigma} = 0.0453]	30451 [R_{int} = 0.0857, R_{sigma} = 0.0665]
**Data / restraints / parameters**	20620 / 262 / 2065	30451 / 1906 / 2385
**Goodness-of-fit on F^2**	1.047	1.086
**Final *R* indexes [I >= 2σ (I)]**	R_1 = 0.0496, wR_2 = 0.1260	R_1 = 0.0830, wR_2 = 0.2299
**Final *R* indexes [all data]**	R_1 = 0.0544, wR_2 = 0.1282	R_1 = 0.0966, wR_2 = 0.2416
**Largest diff. peak/hole / e Å^{-3}**	2.21 / -1.20	2.67 / -2.02
**Flack parameters**	0.088(8)	0.190(9)

Table S6. Crystal data and structure refinement for **4a** and **4b**.

	**4a**	**4b**
**CCDC number**	2216755	2216757
**Empirical formula**	$C_{132}H_{193}Ag_{12}Cl_9N_2O_{33}S_{14}$	$C_{127}H_{187}Ag_{12}Cl_3N_2O_{33}S_{14}$
**Formula weight**	4398.20	4119.40
**Temperature / K**	200	200
**Crystal system**	triclinic	triclinic
**Space group**	$P1$	$P1$
***a* / Å**	15.8156(2)	15.6766(2)
***b* / Å**	17.8033(3)	17.5528(2)
***c* / Å**	17.9216(2)	17.8448(2)
***α* / °**	96.569(1)	99.658(1)
***β* / °**	97.437(1)	96.759(1)
***γ* / °**	116.084(1)	116.466(1)
**Volume / Å^3**	4410.17(11)	4229.10(9)
**Z**	1	1
**ρ_{calc} g / cm^3**	1.656	1.617
**μ / mm^{-1}**	13.753	13.445
**F(000)**	2204.0	2066.0
**Crystal size / mm^3**	0.3 × 0.2 × 0.05	0.3 × 0.22 × 0.06
**Radiation**	Cu $K\alpha$ (λ = 1.54184)	Cu $K\alpha$ (λ = 1.54184)
**2θ range for data collection / °**	5.068 to 147.782	5.148 to 147.884
**Index ranges**	$-18 \le h \le 19$, $-21 \le k \le 13$, $-22 \le l \le 20$	$-19 \le h \le 19$, $-21 \le k \le 21$, $-22 \le l \le 22$
**Reflections collected**	35280	102935
**Independent reflections**	21234 [R_{int} = 0.0734, R_{sigma} = 0.0880]	31025 [R_{int} = 0.1167, R_{sigma} = 0.0807]
**Data / restraints / parameters**	21234 / 587 / 1894	31025 / 481 / 1787
**Goodness-of-fit on F^2**	0.917	1.047
**Final *R* indexes [I >= 2σ (I)]**	R_1 = 0.0842, wR_2 = 0.2257	R_1 = 0.0748, wR_2 = 0.2079
**Final *R* indexes [all data]**	R_1 = 0.0941, wR_2 = 0.2389	R_1 = 0.0828, wR_2 = 0.2188
**Largest diff. peak/hole / e Å^{-3}**	1.92 / -2.99	2.19 / -1.32
**Flack parameters**	0.089(14)	0.080(9)

References

[1] L. J. Bourhis, O. V. Dolomanov, R. J. Gildea, J. A. Howard, H. Puschmann, Acta Crystallogr. Sect. A 2015, **71**, 59–75.
[2] G. M. Sheldrick, Acta Cryst. A 2015, ***71***, 3-8.
[3] O. V. Dolomanov, L. J. Bourhis, R. J. Gildea, J. A. K. Howard, H. Puschmann, J. Appl. Cryst. 2009, ***42***, 339-341
[4] Brandenburg, K. *Diamond*, 2010.
[5] Bannwarth, C.; Caldeweyher, E.; Ehlert, S.; Hansen, A.; Pracht, P.; Seibert, J.; Spicher, S.; Grimme, S. *Comput. Mol. Sci.* **2020**, in press, DOI: 10.1002/wcms.1493.
[6] Bannwarth, C.; Ehlert, S.; Grimme, S. *J. Chem. Theory Comput.* **2019**, *15*, 1652−1671.
[7] Grimme, S.; Bannwarth, C.; Shushkov, P. *J. Chem. Theory Comput.* **2017**, *13*, 1989−2009.
[8] Lu, T.; Chen, F. *J. Comput. Chem.* **2012**, *33*, 580-592.
[9] M. J. Frisch, G. W. Trucks, H. B. Schlegel, G. E. Scuseria, M. A. Robb, J. R. Cheeseman, G. Scalmani, V. Barone, B. Mennucci, G. A. Petersson, et al., Gaussian 16 (Revision A.03). (2016).
[10] W. Humphrey, A. Dalke, K. Schulten, VMD: visual molecular dynamics. *J. Mol. Graphics.* **14**, 33-38 (1996).
[11] Tian Lu, Feiwu Chen, Multiwfn: A multifunctional wavefunction analyzer, J. Comput. Chem., 33, 580-592 (2012).
