# Supplementary material for: Stepwise Amplification of Circularly Polarized Luminescence in Chiral Metal Cluster Ensembles
Source: Adv Sci (Weinh). 2023 Feb 25;10(13):2207660. doi: 10.1002/advs.202207660 (PMC10161016; doi:10.1002/advs.202207660)

checkCIF/PLATON report

You have not supplied any structure factors. As a result the full set of tests cannot be run.

THIS REPORT IS FOR GUIDANCE ONLY. IF USED AS PART OF A REVIEW PROCEDURE FOR PUBLICATION, IT SHOULD NOT REPLACE THE EXPERTISE OF AN EXPERIENCED CRYSTALLOGRAPHIC REFEREE.

No syntax errors found. CIF dictionary Interpreting this report

Datablock: 1a

```
Bond precision:   C-C = 0.0193 A          Wavelength=1.54184

Cell:            a=11.7133(1)      b=13.8082(1)      c=18.2807(2)
                 alpha=84.853(1)   beta=71.513(1)    gamma=84.766(1)
Temperature:     200 K

                 Calculated                   Reported
Volume           2786.65(5)                   2786.65(5)
Space group      P 1                          P 1
Hall group       P 1                          P 1
Moiety formula   C82 H148 Ag12 O28 S12        C82 H148 Ag12 O28 S12
Sum formula      C82 H148 Ag12 O28 S12        C82 H148 Ag12 O28 S12
Mr               3261.17                      3261.16
Dx,g cm-3        1.943                        1.943
Z                1                            1
Mu (mm-1)        19.164                       19.164
F000             1620.0                       1620.0
F000'            1629.42
h,k,lmax         14,17,22                     14,17,22
Nref             22610[ 11305]                13947
Tmin,Tmax        0.025,0.022                  0.228,1.000
Tmin'            0.000

Correction method= # Reported T Limits: Tmin=0.228 Tmax=1.000
AbsCorr = MULTI-SCAN

Data completeness= 1.23/0.62       Theta(max)= 73.849

R(reflections)= 0.0362( 13028)                 wR2(reflections)=
                                               0.0941( 13947)
S = 1.011                 Npar= 1247
```

The following ALERTS were generated. Each ALERT has the format
test-name_ALERT_alert-type_alert-level.
Click on the hyperlinks for more details of the test.

Alert level C

```
SHFSU01_ALERT_2_C  The absolute value of parameter shift to su ratio > 0.05
            Absolute value of the parameter shift to su ratio given   0.060
            Additional refinement cycles may be required.
PLAT080_ALERT_2_C Maximum Shift/Error ............................       0.06 Why ?
PLAT220_ALERT_2_C NonSolvent   Resd 1  C   Ueq(max)/Ueq(min) Range        3.7 Ratio
PLAT220_ALERT_2_C NonSolvent   Resd 1  O   Ueq(max)/Ueq(min) Range        3.8 Ratio
PLAT222_ALERT_3_C NonSolvent Resd 1  H   Uiso(max)/Uiso(min) Range        4.7 Ratio
PLAT241_ALERT_2_C High   'MainMol' Ueq as Compared to Neighbors of        O14 Check
PLAT241_ALERT_2_C High   'MainMol' Ueq as Compared to Neighbors of        O15 Check
PLAT241_ALERT_2_C High   'MainMol' Ueq as Compared to Neighbors of        O19 Check
PLAT241_ALERT_2_C High   'MainMol' Ueq as Compared to Neighbors of        C37 Check
PLAT241_ALERT_2_C High   'MainMol' Ueq as Compared to Neighbors of        C42 Check
PLAT241_ALERT_2_C High   'MainMol' Ueq as Compared to Neighbors of        C47 Check
PLAT242_ALERT_2_C Low    'MainMol' Ueq as Compared to Neighbors of        S12 Check
PLAT242_ALERT_2_C Low    'MainMol' Ueq as Compared to Neighbors of        C36 Check
PLAT242_ALERT_2_C Low    'MainMol' Ueq as Compared to Neighbors of        C43 Check
PLAT242_ALERT_2_C Low    'MainMol' Ueq as Compared to Neighbors of        C49 Check
PLAT242_ALERT_2_C Low    'MainMol' Ueq as Compared to Neighbors of        C51 Check
PLAT242_ALERT_2_C Low    'MainMol' Ueq as Compared to Neighbors of        C80 Check
PLAT342_ALERT_3_C Low Bond Precision on  C-C Bonds ...............    0.01933 Ang.
PLAT601_ALERT_2_C Unit Cell Contains Solvent Accessible VOIDS of .         31 Ang**3
```

Alert level G

```
PLAT002_ALERT_2_G Number of Distance or Angle Restraints on AtSite         16 Note
PLAT003_ALERT_2_G Number of Uiso or Uij Restrained non-H Atoms ...          9 Report
PLAT004_ALERT_5_G Polymeric Structure Found with Maximum Dimension          1 Info
PLAT154_ALERT_1_G The s.u.'s on the Cell Angles are Equal ..(Note)      0.001 Degree
PLAT172_ALERT_4_G The CIF-Embedded .res File Contains DFIX Records          5 Report
PLAT173_ALERT_4_G The CIF-Embedded .res File Contains DANG Records          8 Report
PLAT186_ALERT_4_G The CIF-Embedded .res File Contains ISOR Records          3 Report
PLAT432_ALERT_2_G Short Inter X...Y Contact  O26      ..C69      .       2.96 Ang.
                                       -1+x,1+y,z  =      1_465 Check
PLAT794_ALERT_5_G Tentative Bond Valency for Ag8       (I)       .       1.09 Info
PLAT794_ALERT_5_G Tentative Bond Valency for Ag10      (I)       .       1.12 Info
PLAT860_ALERT_3_G Number of Least-Squares Restraints .............         70 Note
PLAT933_ALERT_2_G Number of HKL-OMIT Records in Embedded .res File          1 Note
PLAT941_ALERT_3_G Average HKL Measurement Multiplicity ...........        2.1 Low
```

0 **ALERT level A** = Most likely a serious problem - resolve or explain
0 **ALERT level B** = A potentially serious problem, consider carefully
19 **ALERT level C** = Check. Ensure it is not caused by an omission or oversight
13 **ALERT level G** = General information/check it is not something unexpected

1 ALERT type 1 CIF construction/syntax error, inconsistent or missing data
21 ALERT type 2 Indicator that the structure model may be wrong or deficient
4 ALERT type 3 Indicator that the structure quality may be low
3 ALERT type 4 Improvement, methodology, query or suggestion
3 ALERT type 5 Informative message, check

Validation response form

Please find below a validation response form (VRF) that can be filled in and pasted into your CIF.

```
# start Validation Reply Form
_vrf_SHFSU01_1a
;
PROBLEM: The absolute value of parameter shift to su ratio > 0.05
RESPONSE: ...
;
_vrf_PLAT080_1a
;
PROBLEM: Maximum Shift/Error ............................       0.06 Why ?
RESPONSE: ...
;
_vrf_PLAT220_1a
;
PROBLEM: NonSolvent   Resd 1  C   Ueq(max)/Ueq(min) Range        3.7 Ratio
RESPONSE: ...
;
_vrf_PLAT222_1a
;
PROBLEM: NonSolvent Resd 1  H   Uiso(max)/Uiso(min) Range        4.7 Ratio
RESPONSE: ...
;
_vrf_PLAT241_1a
;
PROBLEM: High   'MainMol' Ueq as Compared to Neighbors of        O14 Check
RESPONSE: ...
;
_vrf_PLAT242_1a
;
PROBLEM: Low    'MainMol' Ueq as Compared to Neighbors of        S12 Check
RESPONSE: ...
;
_vrf_PLAT342_1a
;
PROBLEM: Low Bond Precision on  C-C Bonds ...............    0.01933 Ang.
RESPONSE: ...
;
_vrf_PLAT601_1a
;
PROBLEM: Unit Cell Contains Solvent Accessible VOIDS of .         31 Ang**3
RESPONSE: ...
;
# end Validation Reply Form
```

It is advisable to attempt to resolve as many as possible of the alerts in all categories. Often the minor alerts point to easily fixed oversights, errors and omissions in your CIF or refinement strategy, so attention to these fine details can be worthwhile. In order to resolve some of the more serious problems it may be necessary to carry out additional measurements or structure refinements. However, the purpose of your study may justify the reported deviations and the more serious of these should normally be commented upon in the discussion or experimental section of a paper or in the "special_details" fields of the CIF. checkCIF was carefully designed to identify outliers and unusual parameters, but every test has its limitations and alerts that are not important in a particular case may appear. Conversely, the absence of alerts does not guarantee there are no aspects of the results needing attention. It is up to the individual to critically assess their own results and, if necessary, seek expert advice.

Publication of your CIF in IUCr journals

A basic structural check has been run on your CIF. These basic checks will be run on all CIFs submitted for publication in IUCr journals (*Acta Crystallographica*, *Journal of Applied Crystallography*, *Journal of Synchrotron Radiation*); however, if you intend to submit to *Acta Crystallographica Section C* or *E* or *IUCrData*, you should make sure that full publication checks are run on the final version of your CIF prior to submission.

Publication of your CIF in other journals

Please refer to the *Notes for Authors* of the relevant journal for any special instructions relating to CIF submission.

PLATON version of 12/09/2022; check.def file version of 09/08/2022

Datablock 1a - ellipsoid plot

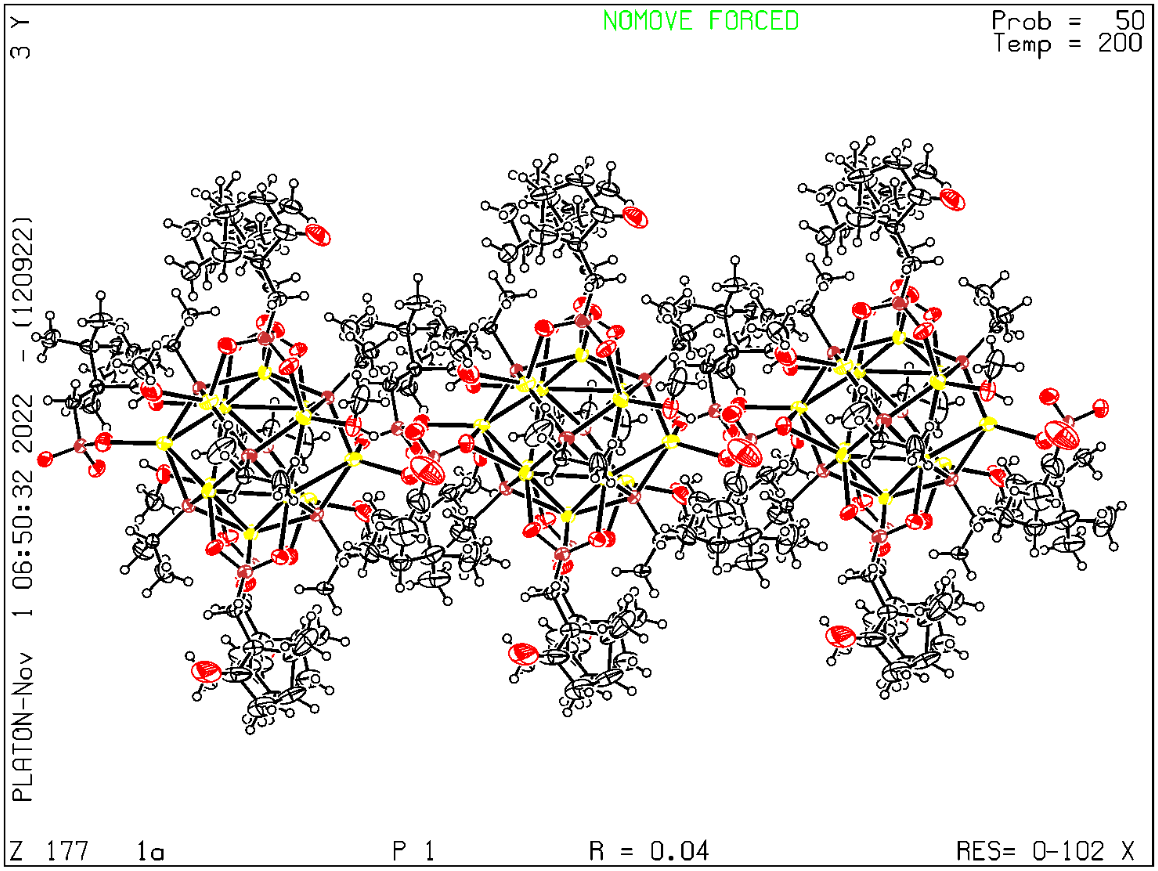

Supplement: Supplementary file 2 — Supporting Information [file ADVS-10-2207660-s002.zip › 1a-checkcif.pdf]
