# Supplementary material for: Stepwise Amplification of Circularly Polarized Luminescence in Chiral Metal Cluster Ensembles
Source: Adv Sci (Weinh). 2023 Feb 25;10(13):2207660. doi: 10.1002/advs.202207660 (PMC10161016; doi:10.1002/advs.202207660)

checkCIF/PLATON report

You have not supplied any structure factors. As a result the full set of tests cannot be run.

THIS REPORT IS FOR GUIDANCE ONLY. IF USED AS PART OF A REVIEW PROCEDURE FOR PUBLICATION, IT SHOULD NOT REPLACE THE EXPERTISE OF AN EXPERIENCED CRYSTALLOGRAPHIC REFEREE.

No syntax errors found. CIF dictionary Interpreting this report

Datablock: 2a

```
Bond precision:   C-C = 0.0339 A          Wavelength=1.54184

Cell:           a=17.3281(2)       b=20.2709(2)      c=24.4284(2)
                alpha=100.583(1)   beta=107.534(1)   gamma=109.862(1)
Temperature:    200 K

                 Calculated                  Reported
Volume           7293.39(16)                 7293.38(14)
Space group      P 1                         P 1
Hall group       P 1                         P 1
                 C118 H172 Ag12 N8 O24 S12,  C103 H154 Ag12 N7 O21 S11,
Moiety formula   C113 H169 Ag12 N7 O25 S12,  C118 H172 Ag12 N8 O24 S12,
                 3(C5 H5 N                   C10 H15 O
Sum formula      C246 H366 Ag24 N18 O54 S24  C246 H366 Ag24 N18 O54 S24
Mr               7797.91                     7797.87
Dx,g cm-3        1.775                       1.775
Z                1                           1
Mu (mm-1)        14.780                      14.780
F000             3912.0                      3912.0
F000'            3932.27
h,k,lmax         21,25,30                    21,25,30
Nref             59618[ 29809]               52972
Tmin,Tmax        0.781,0.863                 0.444,1.000
Tmin'            0.709

Correction method= # Reported T Limits: Tmin=0.444 Tmax=1.000
AbsCorr = MULTI-SCAN

Data completeness= 1.78/0.89       Theta(max)= 74.365
```

R(reflections)= 0.0582(45724)

wR2(reflections)= 0.1720(52972)

S = 1.059 Npar= 3379

The following ALERTS were generated. Each ALERT has the format
test-name_ALERT_alert-type_alert-level.
Click on the hyperlinks for more details of the test.

Alert level B

```
PLAT342_ALERT_3_B Low Bond Precision on  C-C Bonds ...............    0.03392 Ang.
PLAT987_ALERT_1_B The Flack x is >> 0 -  Do a BASF/TWIN Refinement     Please Check
```

Alert level C

```
PLAT220_ALERT_2_C NonSolvent   Resd 1   C   Ueq(max)/Ueq(min) Range        4.0 Ratio
PLAT220_ALERT_2_C NonSolvent   Resd 1   O   Ueq(max)/Ueq(min) Range        3.2 Ratio
PLAT221_ALERT_2_C Solv./Anion  Resd 2   C   Ueq(max)/Ueq(min) Range        5.5 Ratio
PLAT221_ALERT_2_C Solv./Anion  Resd 2   O   Ueq(max)/Ueq(min) Range        5.1 Ratio
PLAT222_ALERT_3_C NonSolvent Resd 1  H   Uiso(max)/Uiso(min) Range         4.9 Ratio
PLAT223_ALERT_4_C Solv./Anion  Resd 2   H   Ueq(max)/Ueq(min) Range        6.9 Ratio
PLAT241_ALERT_2_C High   'MainMol' Ueq as Compared to Neighbors of          O2 Check
PLAT241_ALERT_2_C High   'MainMol' Ueq as Compared to Neighbors of         O15 Check
PLAT241_ALERT_2_C High   'MainMol' Ueq as Compared to Neighbors of         C30 Check
PLAT241_ALERT_2_C High   'MainMol' Ueq as Compared to Neighbors of         C39 Check
PLAT241_ALERT_2_C High   'MainMol' Ueq as Compared to Neighbors of         C57 Check
PLAT241_ALERT_2_C High   'MainMol' Ueq as Compared to Neighbors of        C120 Check
PLAT241_ALERT_2_C High   'MainMol' Ueq as Compared to Neighbors of        C125 Check
PLAT241_ALERT_2_C High   'MainMol' Ueq as Compared to Neighbors of        C147 Check
PLAT241_ALERT_2_C High   'MainMol' Ueq as Compared to Neighbors of        C214 Check
PLAT241_ALERT_2_C High   'MainMol' Ueq as Compared to Neighbors of        C219 Check
PLAT241_ALERT_2_C High   'MainMol' Ueq as Compared to Neighbors of        C220 Check
PLAT241_ALERT_2_C High   'MainMol' Ueq as Compared to Neighbors of        C228 Check
PLAT241_ALERT_2_C High   'MainMol' Ueq as Compared to Neighbors of        Ag24 Check
PLAT241_ALERT_2_C High   'MainMol' Ueq as Compared to Neighbors of         O13 Check
PLAT241_ALERT_2_C High   'MainMol' Ueq as Compared to Neighbors of         O29 Check
PLAT241_ALERT_2_C High   'MainMol' Ueq as Compared to Neighbors of         O30 Check
PLAT241_ALERT_2_C High   'MainMol' Ueq as Compared to Neighbors of         O37 Check
PLAT241_ALERT_2_C High   'MainMol' Ueq as Compared to Neighbors of         O49 Check
PLAT241_ALERT_2_C High   'MainMol' Ueq as Compared to Neighbors of        C175 Check
PLAT241_ALERT_2_C High   'MainMol' Ueq as Compared to Neighbors of        C180 Check
PLAT241_ALERT_2_C High   'MainMol' Ueq as Compared to Neighbors of        C206 Check
PLAT241_ALERT_2_C High   'MainMol' Ueq as Compared to Neighbors of        C207 Check
PLAT241_ALERT_2_C High   'MainMol' Ueq as Compared to Neighbors of        C208 Check
PLAT241_ALERT_2_C High   'MainMol' Ueq as Compared to Neighbors of        C223 Check
PLAT241_ALERT_2_C High   'MainMol' Ueq as Compared to Neighbors of        C236 Check
PLAT241_ALERT_2_C High   'MainMol' Ueq as Compared to Neighbors of        C246 Check
PLAT242_ALERT_2_C Low    'MainMol' Ueq as Compared to Neighbors of          S1 Check
PLAT242_ALERT_2_C Low    'MainMol' Ueq as Compared to Neighbors of          S2 Check
PLAT242_ALERT_2_C Low    'MainMol' Ueq as Compared to Neighbors of         S10 Check
PLAT242_ALERT_2_C Low    'MainMol' Ueq as Compared to Neighbors of         S17 Check
PLAT242_ALERT_2_C Low    'MainMol' Ueq as Compared to Neighbors of         S18 Check
PLAT242_ALERT_2_C Low    'MainMol' Ueq as Compared to Neighbors of         S20 Check
PLAT242_ALERT_2_C Low    'MainMol' Ueq as Compared to Neighbors of        C101 Check
PLAT242_ALERT_2_C Low    'MainMol' Ueq as Compared to Neighbors of        C157 Check
```

```
PLAT242_ALERT_2_C Low    'MainMol' Ueq as Compared to Neighbors of       C164 Check
PLAT242_ALERT_2_C Low    'MainMol' Ueq as Compared to Neighbors of       C184 Check
PLAT242_ALERT_2_C Low    'MainMol' Ueq as Compared to Neighbors of         S6 Check
PLAT242_ALERT_2_C Low    'MainMol' Ueq as Compared to Neighbors of        S11 Check
PLAT242_ALERT_2_C Low    'MainMol' Ueq as Compared to Neighbors of        S23 Check
PLAT242_ALERT_2_C Low    'MainMol' Ueq as Compared to Neighbors of       C182 Check
PLAT242_ALERT_2_C Low    'MainMol' Ueq as Compared to Neighbors of       C186 Check
PLAT242_ALERT_2_C Low    'MainMol' Ueq as Compared to Neighbors of       C195 Check
PLAT242_ALERT_2_C Low    'MainMol' Ueq as Compared to Neighbors of       C229 Check
PLAT242_ALERT_2_C Low    'MainMol' Ueq as Compared to Neighbors of       C237 Check
PLAT242_ALERT_2_C Low    'MainMol' Ueq as Compared to Neighbors of       C239 Check
PLAT242_ALERT_2_C Low    'MainMol' Ueq as Compared to Neighbors of       C242 Check
PLAT243_ALERT_4_C High   'Solvent' Ueq as Compared to Neighbors of        N15 Check
PLAT243_ALERT_4_C High   'Solvent' Ueq as Compared to Neighbors of       C170 Check
PLAT243_ALERT_4_C High   'Solvent' Ueq as Compared to Neighbors of       C202 Check
PLAT244_ALERT_4_C Low    'Solvent' Ueq as Compared to Neighbors of        C99 Check
PLAT260_ALERT_2_C Large Average Ueq of Residue Including       N15      0.113 Check
PLAT260_ALERT_2_C Large Average Ueq of Residue Including       N17      0.109 Check
PLAT260_ALERT_2_C Large Average Ueq of Residue Including       N18      0.125 Check
PLAT260_ALERT_2_C Large Average Ueq of Residue Including       O43      0.101 Check
PLAT260_ALERT_2_C Large Average Ueq of Residue Including       O46      0.153 Check
PLAT329_ALERT_4_C Carbon Atom Hybridisation Unclear for ..........       C181 Check
PLAT360_ALERT_2_C Short  C(sp3)-C(sp3) Bond  C98      - C131     .       1.43 Ang.
PLAT360_ALERT_2_C Short  C(sp3)-C(sp3) Bond  C184     - C194     .       1.43 Ang.
PLAT360_ALERT_2_C Short  C(sp3)-C(sp3) Bond  C79      - C189     .       1.40 Ang.
PLAT361_ALERT_2_C Long   C(sp3)-C(sp3) Bond  C184     - C234     .       1.70 Ang.
PLAT361_ALERT_2_C Long   C(sp3)-C(sp3) Bond  C194     - C220     .       1.70 Ang.
PLAT411_ALERT_2_C Short Inter H...H Contact  H12D     ..H22H     .       2.13 Ang.
                                                  -1+x,y,z  =      1_455 Check
PLAT601_ALERT_2_C Unit Cell Contains Solvent Accessible VOIDS of .         66 Ang**3
```

Alert level G

```
FORMU01_ALERT_1_G  There is a discrepancy between the atom counts in the
            _chemical_formula_sum and _chemical_formula_moiety. This is
            usually due to the moiety formula being in the wrong format.
            Atom count from _chemical_formula_sum:   C246 H366 Ag24 N18 O54 S24
            Atom count from _chemical_formula_moiety:C231 H351 Ag24 N15 O54 S24
PLAT002_ALERT_2_G Number of Distance or Angle Restraints on AtSite         33 Note
PLAT003_ALERT_2_G Number of Uiso or Uij Restrained non-H Atoms ...         77 Report
PLAT007_ALERT_5_G Number of Unrefined Donor-H Atoms .............         12 Report
PLAT033_ALERT_4_G Flack x Value Deviates > 3.0 * sigma from Zero .      0.020 Note
PLAT072_ALERT_2_G SHELXL First  Parameter in WGHT  Unusually Large       0.11 Report
PLAT152_ALERT_1_G The Supplied and Calc. Volume s.u. Differ by ...          2 Units
PLAT154_ALERT_1_G The s.u.'s on the Cell Angles are Equal ..(Note)      0.001 Degree
PLAT172_ALERT_4_G The CIF-Embedded .res File Contains DFIX Records         11 Report
PLAT173_ALERT_4_G The CIF-Embedded .res File Contains DANG Records          5 Report
PLAT176_ALERT_4_G The CIF-Embedded .res File Contains SADI Records          1 Report
PLAT177_ALERT_4_G The CIF-Embedded .res File Contains DELU Records          7 Report
PLAT178_ALERT_4_G The CIF-Embedded .res File Contains SIMU Records          2 Report
PLAT186_ALERT_4_G The CIF-Embedded .res File Contains ISOR Records         13 Report
PLAT300_ALERT_4_G Atom Site Occupancy of C0AA       Constrained at        0.5 Check
PLAT300_ALERT_4_G Atom Site Occupancy of C1AA       Constrained at        0.5 Check
PLAT300_ALERT_4_G Atom Site Occupancy of C190       Constrained at        0.5 Check
PLAT300_ALERT_4_G Atom Site Occupancy of C196       Constrained at        0.5 Check
PLAT300_ALERT_4_G Atom Site Occupancy of H0AA       Constrained at        0.5 Check
PLAT300_ALERT_4_G Atom Site Occupancy of H0AB       Constrained at        0.5 Check
```

```
PLAT300_ALERT_4_G Atom Site Occupancy of H0AC       Constrained at        0.5 Check
PLAT300_ALERT_4_G Atom Site Occupancy of H1AA       Constrained at        0.5 Check
PLAT300_ALERT_4_G Atom Site Occupancy of H1AB       Constrained at        0.5 Check
PLAT300_ALERT_4_G Atom Site Occupancy of H1AC       Constrained at        0.5 Check
PLAT300_ALERT_4_G Atom Site Occupancy of H19A       Constrained at        0.5 Check
PLAT300_ALERT_4_G Atom Site Occupancy of H19B       Constrained at        0.5 Check
PLAT300_ALERT_4_G Atom Site Occupancy of H19C       Constrained at        0.5 Check
PLAT300_ALERT_4_G Atom Site Occupancy of H19G       Constrained at        0.5 Check
PLAT300_ALERT_4_G Atom Site Occupancy of H19H       Constrained at        0.5 Check
PLAT300_ALERT_4_G Atom Site Occupancy of H19I       Constrained at        0.5 Check
PLAT300_ALERT_4_G Atom Site Occupancy of H57        Constrained at        0.5 Check
PLAT300_ALERT_4_G Atom Site Occupancy of H57A       Constrained at        0.5 Check
PLAT300_ALERT_4_G Atom Site Occupancy of S24        Constrained at       0.75 Check
PLAT300_ALERT_4_G Atom Site Occupancy of S25        Constrained at       0.25 Check
PLAT300_ALERT_4_G Atom Site Occupancy of O51        Constrained at       0.75 Check
PLAT300_ALERT_4_G Atom Site Occupancy of O55        Constrained at       0.25 Check
PLAT301_ALERT_3_G Main Residue  Disorder .............(Resd  1  )          1% Note
PLAT302_ALERT_4_G Anion/Solvent/Minor-Residue Disorder (Resd  2  )         2% Note
PLAT343_ALERT_2_G Unusual sp?      Angle Range in Main Residue for       C181 Check
PLAT343_ALERT_2_G Unusual sp3      Angle Range in Main Residue for       C184 Check
PLAT343_ALERT_2_G Unusual sp3      Angle Range in Main Residue for        C85 Check
PLAT343_ALERT_2_G Unusual sp3      Angle Range in Main Residue for       C182 Check
PLAT367_ALERT_2_G Long?  C(sp?)-C(sp?) Bond  C181     - C220     .       1.55 Ang.
PLAT410_ALERT_2_G Short Intra H...H Contact  H23N     ..H1AD     .       2.08 Ang.
                                                      x,y,z  =      1_555 Check
PLAT412_ALERT_2_G Short Intra XH3 .. XHn     H0AC     ..H13J     .       2.04 Ang.
                                                      x,y,z  =      1_555 Check
PLAT412_ALERT_2_G Short Intra XH3 .. XHn     H24K     ..H1AD     .       1.52 Ang.
                                                      x,y,z  =      1_555 Check
PLAT720_ALERT_4_G Number of Unusual/Non-Standard Labels ..........         10 Note
PLAT767_ALERT_4_G INS Embedded LIST 6 Instruction Should be LIST 4     Please Check
PLAT773_ALERT_2_G Check long C-C Bond in CIF: C39      --C181           1.94 Ang.
PLAT773_ALERT_2_G Check long C-C Bond in CIF: C194     --C220           1.71 Ang.
PLAT790_ALERT_4_G Centre of Gravity not Within Unit Cell: Resd.  #          3 Note
              C5 H5 N
PLAT790_ALERT_4_G Centre of Gravity not Within Unit Cell: Resd.  #          5 Note
              C5 H5 N
PLAT790_ALERT_4_G Centre of Gravity not Within Unit Cell: Resd.  #          6 Note
              H2 O
PLAT791_ALERT_4_G Model has Chirality at S2          (Sohnke SpGr)          S Verify
PLAT791_ALERT_4_G Model has Chirality at S5          (Sohnke SpGr)          S Verify
PLAT791_ALERT_4_G Model has Chirality at S13         (Sohnke SpGr)          S Verify
PLAT791_ALERT_4_G Model has Chirality at S18         (Sohnke SpGr)          R Verify
PLAT791_ALERT_4_G Model has Chirality at S20         (Sohnke SpGr)          R Verify
PLAT791_ALERT_4_G Model has Chirality at S21         (Sohnke SpGr)          R Verify
PLAT791_ALERT_4_G Model has Chirality at S24         (Sohnke SpGr)          R Verify
PLAT791_ALERT_4_G Model has Chirality at C15         (Sohnke SpGr)          R Verify
PLAT791_ALERT_4_G Model has Chirality at C26         (Sohnke SpGr)          S Verify
PLAT791_ALERT_4_G Model has Chirality at C46         (Sohnke SpGr)          S Verify
PLAT791_ALERT_4_G Model has Chirality at C54         (Sohnke SpGr)          S Verify
PLAT791_ALERT_4_G Model has Chirality at C76         (Sohnke SpGr)          S Verify
PLAT791_ALERT_4_G Model has Chirality at C83         (Sohnke SpGr)          S Verify
PLAT791_ALERT_4_G Model has Chirality at C85         (Sohnke SpGr)          S Verify
PLAT791_ALERT_4_G Model has Chirality at C93         (Sohnke SpGr)          R Verify
PLAT791_ALERT_4_G Model has Chirality at C105        (Sohnke SpGr)          S Verify
PLAT791_ALERT_4_G Model has Chirality at C112        (Sohnke SpGr)          R Verify
PLAT791_ALERT_4_G Model has Chirality at C126        (Sohnke SpGr)          R Verify
```

```
PLAT791_ALERT_4_G Model has Chirality at C164        (Sohnke SpGr)          S Verify
PLAT791_ALERT_4_G Model has Chirality at C169        (Sohnke SpGr)          R Verify
PLAT791_ALERT_4_G Model has Chirality at C173        (Sohnke SpGr)          R Verify
PLAT791_ALERT_4_G Model has Chirality at C182        (Sohnke SpGr)          S Verify
PLAT791_ALERT_4_G Model has Chirality at C185        (Sohnke SpGr)          R Verify
PLAT791_ALERT_4_G Model has Chirality at C186        (Sohnke SpGr)          R Verify
PLAT791_ALERT_4_G Model has Chirality at C194        (Sohnke SpGr)          R Verify
PLAT791_ALERT_4_G Model has Chirality at C197        (Sohnke SpGr)          R Verify
PLAT791_ALERT_4_G Model has Chirality at C210        (Sohnke SpGr)          R Verify
PLAT791_ALERT_4_G Model has Chirality at C221        (Sohnke SpGr)          S Verify
PLAT791_ALERT_4_G Model has Chirality at C237        (Sohnke SpGr)          S Verify
PLAT791_ALERT_4_G Model has Chirality at C244        (Sohnke SpGr)          R Verify
PLAT794_ALERT_5_G Tentative Bond Valency for Ag7       (I)       .       1.07 Info
PLAT794_ALERT_5_G Tentative Bond Valency for Ag14      (I)       .       1.05 Info
PLAT860_ALERT_3_G Number of Least-Squares Restraints ............         543 Note
PLAT883_ALERT_1_G No Info/Value for _atom_sites_solution_primary .     Please Do !
PLAT933_ALERT_2_G Number of HKL-OMIT Records in Embedded .res File          6 Note
```

```
  0 ALERT level A = Most likely a serious problem - resolve or explain
  2 ALERT level B = A potentially serious problem, consider carefully
 69 ALERT level C = Check. Ensure it is not caused by an omission or oversight
 88 ALERT level G = General information/check it is not something unexpected

  5 ALERT type 1 CIF construction/syntax error, inconsistent or missing data
 76 ALERT type 2 Indicator that the structure model may be wrong or deficient
  4 ALERT type 3 Indicator that the structure quality may be low
 71 ALERT type 4 Improvement, methodology, query or suggestion
  3 ALERT type 5 Informative message, check
```

It is advisable to attempt to resolve as many as possible of the alerts in all categories. Often the minor alerts point to easily fixed oversights, errors and omissions in your CIF or refinement strategy, so attention to these fine details can be worthwhile. In order to resolve some of the more serious problems it may be necessary to carry out additional measurements or structure refinements. However, the purpose of your study may justify the reported deviations and the more serious of these should normally be commented upon in the discussion or experimental section of a paper or in the "special_details" fields of the CIF. checkCIF was carefully designed to identify outliers and unusual parameters, but every test has its limitations and alerts that are not important in a particular case may appear. Conversely, the absence of alerts does not guarantee there are no aspects of the results needing attention. It is up to the individual to critically assess their own results and, if necessary, seek expert advice.

Publication of your CIF in IUCr journals

A basic structural check has been run on your CIF. These basic checks will be run on all CIFs submitted for publication in IUCr journals (*Acta Crystallographica*, *Journal of Applied Crystallography*, *Journal of Synchrotron Radiation*); however, if you intend to submit to *Acta Crystallographica Section C* or *E* or *IUCrData*, you should make sure that full publication checks are run on the final version of your CIF prior to submission.

Publication of your CIF in other journals

Please refer to the *Notes for Authors* of the relevant journal for any special instructions relating to CIF submission.

PLATON version of 12/09/2022; check.def file version of 09/08/2022

Datablock 2a - ellipsoid plot

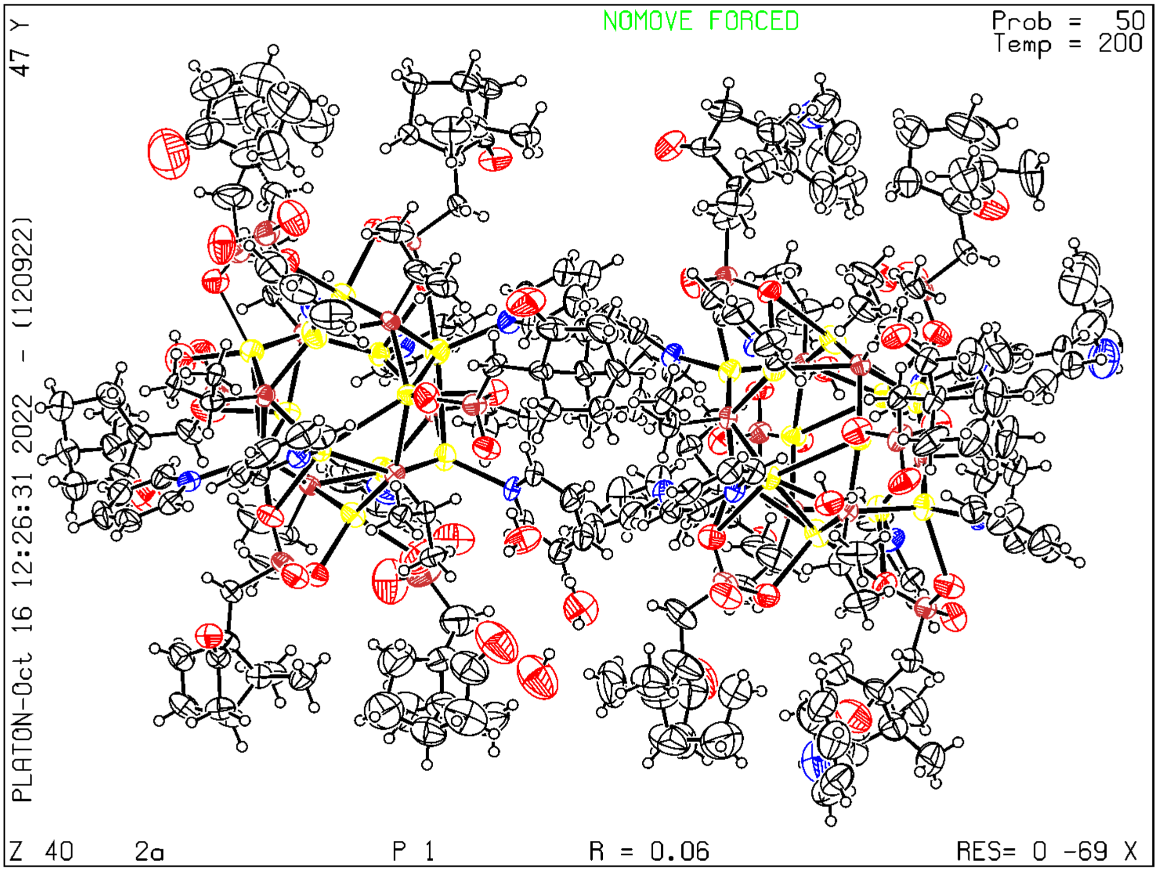

Supplement: Supplementary file 2 — Supporting Information [file ADVS-10-2207660-s002.zip › 2a-checkcif.pdf]
