# Supplementary material for: Stepwise Amplification of Circularly Polarized Luminescence in Chiral Metal Cluster Ensembles
Source: Adv Sci (Weinh). 2023 Feb 25;10(13):2207660. doi: 10.1002/advs.202207660 (PMC10161016; doi:10.1002/advs.202207660)

checkCIF/PLATON report

You have not supplied any structure factors. As a result the full set of tests cannot be run.

THIS REPORT IS FOR GUIDANCE ONLY. IF USED AS PART OF A REVIEW PROCEDURE FOR PUBLICATION, IT SHOULD NOT REPLACE THE EXPERTISE OF AN EXPERIENCED CRYSTALLOGRAPHIC REFEREE.

No syntax errors found.   CIF dictionary   Interpreting this report

Datablock: 2b

```
Bond precision:   C-C = 0.0242 A          Wavelength=1.54184

Cell:          a=17.30140(11)     b=20.20659(12)    c=24.36782(13)
               alpha=100.5975(5)  beta=107.8152(5)  gamma=109.6734(6)
Temperature:   150 K

                Calculated                  Reported
Volume          7235.42(9)                  7235.41(8)
Space group     P 1                         P 1
Hall group      P 1                         P 1
                C118 H172 Ag12 N8 O24 S12,  C118 H172 Ag12 N8 O24 S12,
Moiety formula  C113 H169 Ag12 N7 O25 S12,  C113 H169 Ag12 N7 O25 S12,
                3(C5 H5 N                   6(H2 O),
Sum formula     C246 H368 Ag24 N18 O55 S24  C246 H368 Ag24 N18 O55 S24
Mr              7815.93                     7815.88
Dx,g cm-3       1.794                       1.794
Z               1                           1
Mu (mm-1)       14.903                      14.903
F000            3922.0                      3922.0
F000'           3942.32
h,k,lmax        21,25,30                    21,25,30
Nref            58720[ 29360]               52339
Tmin,Tmax       0.209,0.225                 0.564,1.000
Tmin'           0.134

Correction method= # Reported T Limits: Tmin=0.564 Tmax=1.000
AbsCorr = MULTI-SCAN

Data completeness= 1.78/0.89       Theta(max)= 73.976
```

R(reflections)= 0.0525(48530)

wR2(reflections)= 0.1363(52339)

S = 1.019 Npar= 3388

The following ALERTS were generated. Each ALERT has the format
test-name_ALERT_alert-type_alert-level.
Click on the hyperlinks for more details of the test.

Alert level B

PLAT221_ALERT_2_B Solv./Anion Resd 2 C Ueq(max)/Ueq(min) Range 8.1 Ratio
PLAT342_ALERT_3_B Low Bond Precision on C-C Bonds .... 0.02423 Ang.
PLAT987_ALERT_1_B The Flack x is >> 0 - Do a BASF/TWIN Refinement Please Check

---

Alert level C

PLAT042_ALERT_1_C Calc. and Reported MoietyFormula Strings Differ Please Check
PLAT213_ALERT_2_C Atom C69 has ADP max/min Ratio ..... 3.9 prolat
PLAT213_ALERT_2_C Atom C131 has ADP max/min Ratio ..... 3.7 prolat
PLAT213_ALERT_2_C Atom C216 has ADP max/min Ratio ..... 3.3 prolat
PLAT214_ALERT_2_C Atom C83 (Anion/Solvent) ADP max/min Ratio 4.4 prolat
PLAT220_ALERT_2_C NonSolvent Resd 1 C Ueq(max)/Ueq(min) Range 5.8 Ratio
PLAT221_ALERT_2_C Solv./Anion Resd 2 O Ueq(max)/Ueq(min) Range 6.6 Ratio
PLAT222_ALERT_3_C NonSolvent Resd 1 H Uiso(max)/Uiso(min) Range 7.2 Ratio
PLAT223_ALERT_4_C Solv./Anion Resd 2 H Ueq(max)/Ueq(min) Range 10.0 Ratio
PLAT241_ALERT_2_C High 'MainMol' Ueq as Compared to Neighbors of C180 Check
PLAT241_ALERT_2_C High 'MainMol' Ueq as Compared to Neighbors of C193 Check
PLAT241_ALERT_2_C High 'MainMol' Ueq as Compared to Neighbors of Ag24 Check
PLAT241_ALERT_2_C High 'MainMol' Ueq as Compared to Neighbors of O31 Check
PLAT241_ALERT_2_C High 'MainMol' Ueq as Compared to Neighbors of O43 Check
PLAT241_ALERT_2_C High 'MainMol' Ueq as Compared to Neighbors of O54 Check
PLAT241_ALERT_2_C High 'MainMol' Ueq as Compared to Neighbors of C83 Check
PLAT241_ALERT_2_C High 'MainMol' Ueq as Compared to Neighbors of C165 Check
PLAT241_ALERT_2_C High 'MainMol' Ueq as Compared to Neighbors of C186 Check
PLAT241_ALERT_2_C High 'MainMol' Ueq as Compared to Neighbors of C230 Check
PLAT241_ALERT_2_C High 'MainMol' Ueq as Compared to Neighbors of C231 Check
PLAT241_ALERT_2_C High 'MainMol' Ueq as Compared to Neighbors of C234 Check
PLAT241_ALERT_2_C High 'MainMol' Ueq as Compared to Neighbors of C239 Check
PLAT241_ALERT_2_C High 'MainMol' Ueq as Compared to Neighbors of C246 Check
PLAT242_ALERT_2_C Low 'MainMol' Ueq as Compared to Neighbors of S7 Check
PLAT242_ALERT_2_C Low 'MainMol' Ueq as Compared to Neighbors of S9 Check
PLAT242_ALERT_2_C Low 'MainMol' Ueq as Compared to Neighbors of S11 Check
PLAT242_ALERT_2_C Low 'MainMol' Ueq as Compared to Neighbors of C64 Check
PLAT242_ALERT_2_C Low 'MainMol' Ueq as Compared to Neighbors of C112 Check
PLAT242_ALERT_2_C Low 'MainMol' Ueq as Compared to Neighbors of S1 Check
PLAT242_ALERT_2_C Low 'MainMol' Ueq as Compared to Neighbors of S22 Check
PLAT242_ALERT_2_C Low 'MainMol' Ueq as Compared to Neighbors of S23 Check
PLAT242_ALERT_2_C Low 'MainMol' Ueq as Compared to Neighbors of C132 Check
PLAT242_ALERT_2_C Low 'MainMol' Ueq as Compared to Neighbors of C137 Check
PLAT242_ALERT_2_C Low 'MainMol' Ueq as Compared to Neighbors of C147 Check
PLAT242_ALERT_2_C Low 'MainMol' Ueq as Compared to Neighbors of C226 Check
PLAT242_ALERT_2_C Low 'MainMol' Ueq as Compared to Neighbors of C243 Check
PLAT243_ALERT_4_C High 'Solvent' Ueq as Compared to Neighbors of N15 Check
PLAT243_ALERT_4_C High 'Solvent' Ueq as Compared to Neighbors of N17 Check
PLAT244_ALERT_4_C Low 'Solvent' Ueq as Compared to Neighbors of C41 Check

```
PLAT244_ALERT_4_C Low    'Solvent' Ueq as Compared to Neighbors of        C82 Check
PLAT260_ALERT_2_C Large Average Ueq of Residue Including       O4W      0.129 Check
PLAT329_ALERT_4_C Carbon Atom Hybridisation Unclear for .........       C211 Check
PLAT360_ALERT_2_C Short  C(sp3)-C(sp3) Bond  C69      - C227     .       1.35 Ang.
PLAT360_ALERT_2_C Short  C(sp3)-C(sp3) Bond  C139     - C227     .       1.43 Ang.
PLAT361_ALERT_2_C Long   C(sp3)-C(sp3) Bond  C199     - C207     .       1.65 Ang.
PLAT361_ALERT_2_C Long   C(sp3)-C(sp3) Bond  C210     - C240     .       1.67 Ang.
PLAT411_ALERT_2_C Short Inter H...H Contact  H17D     ..H45A     .       2.08 Ang.
                                                  -1+x,y,z  =      1_455 Check
PLAT411_ALERT_2_C Short Inter H...H Contact  H206     ..H226     .       2.06 Ang.
                                                 1+x,y,1+z  =      1_656 Check
PLAT601_ALERT_2_C Unit Cell Contains Solvent Accessible VOIDS of .         64 Ang**3
```

Alert level G

```
PLAT002_ALERT_2_G Number of Distance or Angle Restraints on AtSite         10 Note
PLAT003_ALERT_2_G Number of Uiso or Uij Restrained non-H Atoms ...         35 Report
PLAT007_ALERT_5_G Number of Unrefined Donor-H Atoms .............          14 Report
PLAT033_ALERT_4_G Flack x Value Deviates > 3.0 * sigma from Zero .      0.023 Note
PLAT172_ALERT_4_G The CIF-Embedded .res File Contains DFIX Records          3 Report
PLAT176_ALERT_4_G The CIF-Embedded .res File Contains SADI Records          1 Report
PLAT177_ALERT_4_G The CIF-Embedded .res File Contains DELU Records          1 Report
PLAT178_ALERT_4_G The CIF-Embedded .res File Contains SIMU Records          2 Report
PLAT186_ALERT_4_G The CIF-Embedded .res File Contains ISOR Records         11 Report
PLAT300_ALERT_4_G Atom Site Occupancy of S24        Constrained at       0.75 Check
PLAT300_ALERT_4_G Atom Site Occupancy of S0AA       Constrained at       0.25 Check
PLAT300_ALERT_4_G Atom Site Occupancy of O30        Constrained at       0.75 Check
PLAT300_ALERT_4_G Atom Site Occupancy of O45        Constrained at       0.75 Check
PLAT300_ALERT_4_G Atom Site Occupancy of O178       Constrained at       0.25 Check
PLAT300_ALERT_4_G Atom Site Occupancy of O217       Constrained at       0.25 Check
PLAT300_ALERT_4_G Atom Site Occupancy of C244       Constrained at       0.75 Check
PLAT300_ALERT_4_G Atom Site Occupancy of C0AA       Constrained at       0.25 Check
PLAT300_ALERT_4_G Atom Site Occupancy of H24C       Constrained at       0.75 Check
PLAT300_ALERT_4_G Atom Site Occupancy of H24D       Constrained at       0.75 Check
PLAT300_ALERT_4_G Atom Site Occupancy of H0AA       Constrained at       0.25 Check
PLAT300_ALERT_4_G Atom Site Occupancy of H0AB       Constrained at       0.25 Check
PLAT302_ALERT_4_G Anion/Solvent/Minor-Residue Disorder (Resd  2  )         2% Note
PLAT343_ALERT_2_G Unusual sp?      Angle Range in Main Residue for       C103 Check
PLAT343_ALERT_2_G Unusual sp?      Angle Range in Main Residue for       C211 Check
PLAT343_ALERT_2_G Unusual sp3      Angle Range in Main Residue for       C130 Check
PLAT367_ALERT_2_G Long?  C(sp?)-C(sp?) Bond  C103     - C148     .       1.54 Ang.
PLAT367_ALERT_2_G Long?  C(sp?)-C(sp?) Bond  C211     - C240     .       1.53 Ang.
PLAT412_ALERT_2_G Short Intra XH3 .. XHn     H22G     ..H0AB     .       1.60 Ang.
                                                    x,y,z  =      1_555 Check
PLAT720_ALERT_4_G Number of Unusual/Non-Standard Labels .........          18 Note
PLAT722_ALERT_1_G Angle   Calc      116.00, Rep      117.10 Dev...       1.10 Degree
              C245    -C186    -H186       1_555  1_555  1_555     # 1486 Check
PLAT773_ALERT_2_G Check long C-C Bond in CIF: C103     --C211            1.79 Ang.
PLAT790_ALERT_4_G Centre of Gravity not Within Unit Cell: Resd.  #          2 Note
              C113 H169 Ag12 N7 O25 S12
PLAT790_ALERT_4_G Centre of Gravity not Within Unit Cell: Resd.  #          3 Note
              C5 H5 N
PLAT790_ALERT_4_G Centre of Gravity not Within Unit Cell: Resd.  #          4 Note
              C5 H5 N
PLAT790_ALERT_4_G Centre of Gravity not Within Unit Cell: Resd.  #          6 Note
              H2 O
PLAT790_ALERT_4_G Centre of Gravity not Within Unit Cell: Resd.  #          8 Note
```

```
              H2 O
PLAT790_ALERT_4_G Centre of Gravity not Within Unit Cell: Resd.  #          9 Note
              H2 O
PLAT790_ALERT_4_G Centre of Gravity not Within Unit Cell: Resd.  #         10 Note
              H2 O
PLAT790_ALERT_4_G Centre of Gravity not Within Unit Cell: Resd.  #         11 Note
              H2 O
PLAT791_ALERT_4_G Model has Chirality at S3          (Sohnke SpGr)          R Verify
PLAT791_ALERT_4_G Model has Chirality at S6          (Sohnke SpGr)          R Verify
PLAT791_ALERT_4_G Model has Chirality at S11         (Sohnke SpGr)          S Verify
PLAT791_ALERT_4_G Model has Chirality at S13         (Sohnke SpGr)          S Verify
PLAT791_ALERT_4_G Model has Chirality at S16         (Sohnke SpGr)          R Verify
PLAT791_ALERT_4_G Model has Chirality at S18         (Sohnke SpGr)          S Verify
PLAT791_ALERT_4_G Model has Chirality at S24         (Sohnke SpGr)          S Verify
PLAT791_ALERT_4_G Model has Chirality at C1          (Sohnke SpGr)          S Verify
PLAT791_ALERT_4_G Model has Chirality at C19         (Sohnke SpGr)          R Verify
PLAT791_ALERT_4_G Model has Chirality at C23         (Sohnke SpGr)          R Verify
PLAT791_ALERT_4_G Model has Chirality at C73         (Sohnke SpGr)          R Verify
PLAT791_ALERT_4_G Model has Chirality at C78         (Sohnke SpGr)          R Verify
PLAT791_ALERT_4_G Model has Chirality at C100        (Sohnke SpGr)          S Verify
PLAT791_ALERT_4_G Model has Chirality at C109        (Sohnke SpGr)          S Verify
PLAT791_ALERT_4_G Model has Chirality at C112        (Sohnke SpGr)          R Verify
PLAT791_ALERT_4_G Model has Chirality at C113        (Sohnke SpGr)          R Verify
PLAT791_ALERT_4_G Model has Chirality at C126        (Sohnke SpGr)          S Verify
PLAT791_ALERT_4_G Model has Chirality at C130        (Sohnke SpGr)          R Verify
PLAT791_ALERT_4_G Model has Chirality at C131        (Sohnke SpGr)          S Verify
PLAT791_ALERT_4_G Model has Chirality at C132        (Sohnke SpGr)          R Verify
PLAT791_ALERT_4_G Model has Chirality at C141        (Sohnke SpGr)          R Verify
PLAT791_ALERT_4_G Model has Chirality at C188        (Sohnke SpGr)          S Verify
PLAT791_ALERT_4_G Model has Chirality at C192        (Sohnke SpGr)          S Verify
PLAT791_ALERT_4_G Model has Chirality at C195        (Sohnke SpGr)          R Verify
PLAT791_ALERT_4_G Model has Chirality at C210        (Sohnke SpGr)          S Verify
PLAT791_ALERT_4_G Model has Chirality at C226        (Sohnke SpGr)          S Verify
PLAT791_ALERT_4_G Model has Chirality at C232        (Sohnke SpGr)          S Verify
PLAT791_ALERT_4_G Model has Chirality at C241        (Sohnke SpGr)          S Verify
PLAT794_ALERT_5_G Tentative Bond Valency for Ag11      (I)       .       1.05 Info
PLAT860_ALERT_3_G Number of Least-Squares Restraints ............        254 Note
PLAT883_ALERT_1_G No Info/Value for _atom_sites_solution_primary .     Please Do !
PLAT933_ALERT_2_G Number of HKL-OMIT Records in Embedded .res File          2 Note
```

```
   0 ALERT level A = Most likely a serious problem - resolve or explain
   3 ALERT level B = A potentially serious problem, consider carefully
  49 ALERT level C = Check. Ensure it is not caused by an omission or oversight
  71 ALERT level G = General information/check it is not something unexpected

   4 ALERT type 1 CIF construction/syntax error, inconsistent or missing data
  52 ALERT type 2 Indicator that the structure model may be wrong or deficient
   3 ALERT type 3 Indicator that the structure quality may be low
  62 ALERT type 4 Improvement, methodology, query or suggestion
   2 ALERT type 5 Informative message, check
```

It is advisable to attempt to resolve as many as possible of the alerts in all categories. Often the minor alerts point to easily fixed oversights, errors and omissions in your CIF or refinement strategy, so attention to these fine details can be worthwhile. In order to resolve some of the more serious problems it may be necessary to carry out additional measurements or structure refinements. However, the purpose of your study may justify the reported deviations and the more serious of these should normally be commented upon in the discussion or experimental section of a paper or in the "special_details" fields of the CIF. checkCIF was carefully designed to identify outliers and unusual parameters, but every test has its limitations and alerts that are not important in a particular case may appear. Conversely, the absence of alerts does not guarantee there are no aspects of the results needing attention. It is up to the individual to critically assess their own results and, if necessary, seek expert advice.

Publication of your CIF in IUCr journals

A basic structural check has been run on your CIF. These basic checks will be run on all CIFs submitted for publication in IUCr journals (*Acta Crystallographica*, *Journal of Applied Crystallography*, *Journal of Synchrotron Radiation*); however, if you intend to submit to *Acta Crystallographica Section C* or *E* or *IUCrData*, you should make sure that full publication checks are run on the final version of your CIF prior to submission.

Publication of your CIF in other journals

Please refer to the *Notes for Authors* of the relevant journal for any special instructions relating to CIF submission.

PLATON version of 12/09/2022; check.def file version of 09/08/2022

Datablock 2b - ellipsoid plot

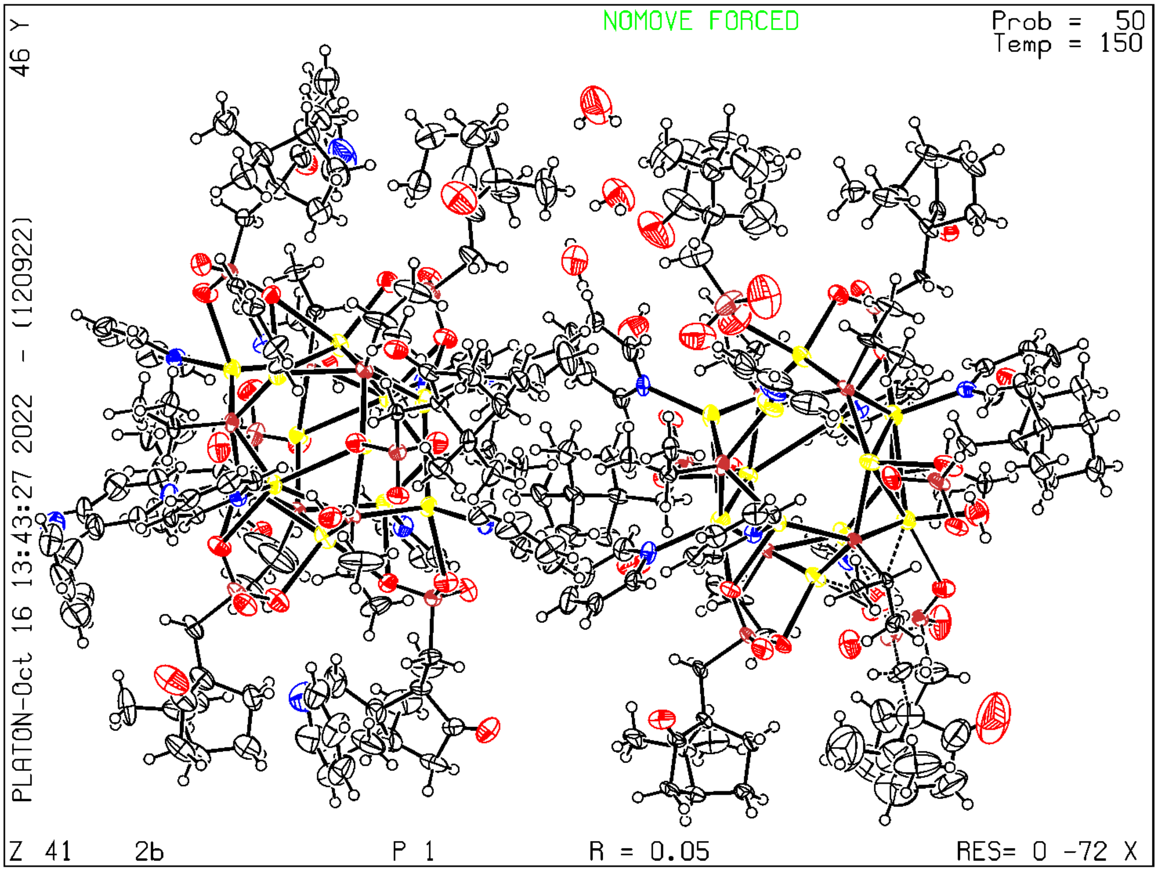

Supplement: Supplementary file 2 — Supporting Information [file ADVS-10-2207660-s002.zip › 2b-checkcif.pdf]
