# Supplementary material for: Stepwise Amplification of Circularly Polarized Luminescence in Chiral Metal Cluster Ensembles
Source: Adv Sci (Weinh). 2023 Feb 25;10(13):2207660. doi: 10.1002/advs.202207660 (PMC10161016; doi:10.1002/advs.202207660)

checkCIF/PLATON report

You have not supplied any structure factors. As a result the full set of tests cannot be run.

THIS REPORT IS FOR GUIDANCE ONLY. IF USED AS PART OF A REVIEW PROCEDURE FOR PUBLICATION, IT SHOULD NOT REPLACE THE EXPERTISE OF AN EXPERIENCED CRYSTALLOGRAPHIC REFEREE.

No syntax errors found.   CIF dictionary   Interpreting this report

Datablock: 3a

Bond precision:	C-C = 0.0262 A		Wavelength=1.54184
Cell:	a=15.4455(1)	b=15.6831(1)	c=19.3575(2)
	alpha=80.103(1)	beta=81.439(1)	gamma=65.512(1)
Temperature:	200 K		

	Calculated	Reported
Volume	4187.96(7)	4187.96(7)
Space group	P 1	P 1
Hall group	P 1	P 1
Moiety formula	C162 H192 Ag12 N6 O24 S12, 2(H2 O)	C162 H192 Ag12 N6 O24 S12, 2(H2 O)
Sum formula	C162 H196 Ag12 N6 O26 S12	C162 H196 Ag12 N6 O26 S12
Mr	4322.38	4322.40
Dx,g cm-3	1.714	1.714
Z	1	1
Mu (mm-1)	12.934	12.934
F000	2174.0	2174.0
F000'	2184.60	
h,k,lmax	19,19,24	19,19,24
Nref	33872[16936]	20620
Tmin,Tmax	0.146,0.524	0.313,1.000
Tmin'	0.018	

Correction method= # Reported T Limits: Tmin=0.313 Tmax=1.000
AbsCorr = MULTI-SCAN

Data completeness= 1.22/0.61   Theta(max)= 73.754

R(reflections)= 0.0496(19118)   wR2(reflections)= 0.1282(20620)

S = 1.047   Npar= 2065

The following ALERTS were generated. Each ALERT has the format
test-name_ALERT_alert-type_alert-level.
Click on the hyperlinks for more details of the test.

Alert level B

PLAT342_ALERT_3_B Low Bond Precision on C-C Bonds .... 0.02616 Ang.
PLAT987_ALERT_1_B The Flack x is >> 0 - Do a BASF/TWIN Refinement Please Check

Alert level C

SHFSU01_ALERT_2_C The absolute value of parameter shift to su ratio > 0.05
Absolute value of the parameter shift to su ratio given 0.056
Additional refinement cycles may be required.
PLAT080_ALERT_2_C Maximum Shift/Error .... 0.06 Why ?
PLAT213_ALERT_2_C Atom C65 has ADP max/min Ratio ..... 3.6 prolat
PLAT220_ALERT_2_C NonSolvent Resd 1 C Ueq(max)/Ueq(min) Range 5.7 Ratio
PLAT220_ALERT_2_C NonSolvent Resd 1 S Ueq(max)/Ueq(min) Range 3.1 Ratio
PLAT222_ALERT_3_C NonSolvent Resd 1 H Uiso(max)/Uiso(min) Range 5.9 Ratio
PLAT241_ALERT_2_C High 'MainMol' Ueq as Compared to Neighbors of O5 Check
PLAT241_ALERT_2_C High 'MainMol' Ueq as Compared to Neighbors of O11 Check
PLAT241_ALERT_2_C High 'MainMol' Ueq as Compared to Neighbors of O13 Check
PLAT241_ALERT_2_C High 'MainMol' Ueq as Compared to Neighbors of O14 Check
PLAT241_ALERT_2_C High 'MainMol' Ueq as Compared to Neighbors of O15 Check
PLAT241_ALERT_2_C High 'MainMol' Ueq as Compared to Neighbors of O18 Check
PLAT241_ALERT_2_C High 'MainMol' Ueq as Compared to Neighbors of O23 Check
PLAT241_ALERT_2_C High 'MainMol' Ueq as Compared to Neighbors of C54 Check
PLAT241_ALERT_2_C High 'MainMol' Ueq as Compared to Neighbors of C56 Check
PLAT241_ALERT_2_C High 'MainMol' Ueq as Compared to Neighbors of C111 Check
PLAT241_ALERT_2_C High 'MainMol' Ueq as Compared to Neighbors of C143 Check
PLAT241_ALERT_2_C High 'MainMol' Ueq as Compared to Neighbors of C154 Check
PLAT242_ALERT_2_C Low 'MainMol' Ueq as Compared to Neighbors of S2 Check
PLAT242_ALERT_2_C Low 'MainMol' Ueq as Compared to Neighbors of S9 Check
PLAT242_ALERT_2_C Low 'MainMol' Ueq as Compared to Neighbors of S10 Check
PLAT242_ALERT_2_C Low 'MainMol' Ueq as Compared to Neighbors of S11 Check
PLAT242_ALERT_2_C Low 'MainMol' Ueq as Compared to Neighbors of C9 Check
PLAT242_ALERT_2_C Low 'MainMol' Ueq as Compared to Neighbors of C15 Check
PLAT242_ALERT_2_C Low 'MainMol' Ueq as Compared to Neighbors of C28 Check
PLAT242_ALERT_2_C Low 'MainMol' Ueq as Compared to Neighbors of C32 Check
PLAT242_ALERT_2_C Low 'MainMol' Ueq as Compared to Neighbors of C42 Check
PLAT242_ALERT_2_C Low 'MainMol' Ueq as Compared to Neighbors of C47 Check
PLAT242_ALERT_2_C Low 'MainMol' Ueq as Compared to Neighbors of C52 Check
PLAT242_ALERT_2_C Low 'MainMol' Ueq as Compared to Neighbors of C57 Check
PLAT242_ALERT_2_C Low 'MainMol' Ueq as Compared to Neighbors of C78 Check
PLAT260_ALERT_2_C Large Average Ueq of Residue Including O0AA 0.105 Check
PLAT260_ALERT_2_C Large Average Ueq of Residue Including O1AA 0.165 Check
PLAT360_ALERT_2_C Short C(sp3)-C(sp3) Bond C51 - C52 . 1.40 Ang.
PLAT360_ALERT_2_C Short C(sp3)-C(sp3) Bond C62 - C63 . 1.42 Ang.
PLAT360_ALERT_2_C Short C(sp3)-C(sp3) Bond C64 - C65 . 1.43 Ang.
PLAT361_ALERT_2_C Long C(sp3)-C(sp3) Bond C62 - C68 . 1.74 Ang.
PLAT362_ALERT_2_C Short C(sp3)-C(sp2) Bond C26 - C27 . 1.38 Ang.
PLAT767_ALERT_4_C INS Embedded LIST 6 Instruction Should be LIST 4 Please Check

Alert level G

```
PLAT002_ALERT_2_G Number of Distance or Angle Restraints on AtSite         19 Note
PLAT003_ALERT_2_G Number of Uiso or Uij Restrained non-H Atoms ...         40 Report
PLAT004_ALERT_5_G Polymeric Structure Found with Maximum Dimension          2 Info
PLAT007_ALERT_5_G Number of Unrefined Donor-H Atoms .............           4 Report
PLAT033_ALERT_4_G Flack x Value Deviates > 3.0 * sigma from Zero .      0.088 Note
PLAT083_ALERT_2_G SHELXL Second Parameter in WGHT  Unusually Large      20.08 Why ?
PLAT154_ALERT_1_G The s.u.'s on the Cell Angles are Equal ..(Note)      0.001 Degree
PLAT172_ALERT_4_G The CIF-Embedded .res File Contains DFIX Records          3 Report
PLAT173_ALERT_4_G The CIF-Embedded .res File Contains DANG Records          1 Report
PLAT176_ALERT_4_G The CIF-Embedded .res File Contains SADI Records          1 Report
PLAT177_ALERT_4_G The CIF-Embedded .res File Contains DELU Records          2 Report
PLAT186_ALERT_4_G The CIF-Embedded .res File Contains ISOR Records         10 Report
PLAT301_ALERT_3_G Main Residue  Disorder .............(Resd  1  )          5% Note
PLAT343_ALERT_2_G Unusual sp3      Angle Range in Main Residue for        C51 Check
PLAT410_ALERT_2_G Short Intra H...H Contact  H1AA     ..H15      .       1.75 Ang.
                                                x,-1+y,-1+z  =      1_544 Check
PLAT412_ALERT_2_G Short Intra XH3 .. XHn     H13C     ..H104     .       2.00 Ang.
                                                  x,1+y,1+z  =      1_566 Check
PLAT720_ALERT_4_G Number of Unusual/Non-Standard Labels ..........         18 Note
PLAT773_ALERT_2_G Check long C-C Bond in CIF: C62      --C68             1.74 Ang.
PLAT860_ALERT_3_G Number of Least-Squares Restraints ............         262 Note
PLAT870_ALERT_4_G ALERTS Related to Twinning Effects Suppressed ..          ! Info
PLAT933_ALERT_2_G Number of HKL-OMIT Records in Embedded .res File         15 Note
PLAT941_ALERT_3_G Average HKL Measurement Multiplicity ..........         2.7 Low
```

```
   0 ALERT level A = Most likely a serious problem - resolve or explain
   2 ALERT level B = A potentially serious problem, consider carefully
  39 ALERT level C = Check. Ensure it is not caused by an omission or oversight
  22 ALERT level G = General information/check it is not something unexpected

   2 ALERT type 1 CIF construction/syntax error, inconsistent or missing data
  45 ALERT type 2 Indicator that the structure model may be wrong or deficient
   5 ALERT type 3 Indicator that the structure quality may be low
   9 ALERT type 4 Improvement, methodology, query or suggestion
   2 ALERT type 5 Informative message, check
```

Validation response form

Please find below a validation response form (VRF) that can be filled in and pasted into your CIF.

```
# start Validation Reply Form
_vrf_SHFSU01_3a
;
PROBLEM: The absolute value of parameter shift to su ratio > 0.05
RESPONSE: ...
;
_vrf_PLAT342_3a
;
PROBLEM: Low Bond Precision on  C-C Bonds ..............    0.02616 Ang.
RESPONSE: ...
;
_vrf_PLAT987_3a
;
PROBLEM: The Flack x is >> 0 -  Do a BASF/TWIN Refinement     Please Check
RESPONSE: ...
```

```
;
_vrf_PLAT080_3a
;
PROBLEM: Maximum Shift/Error ............................       0.06 Why ?
RESPONSE: ...
;
_vrf_PLAT213_3a
;
PROBLEM: Atom C65             has ADP max/min Ratio .....        3.6 prolat
RESPONSE: ...
;
_vrf_PLAT220_3a
;
PROBLEM: NonSolvent   Resd 1  C   Ueq(max)/Ueq(min) Range        5.7 Ratio
RESPONSE: ...
;
_vrf_PLAT222_3a
;
PROBLEM: NonSolvent Resd 1  H   Uiso(max)/Uiso(min) Range        5.9 Ratio
RESPONSE: ...
;
_vrf_PLAT241_3a
;
PROBLEM: High   'MainMol' Ueq as Compared to Neighbors of         O5 Check
RESPONSE: ...
;
_vrf_PLAT242_3a
;
PROBLEM: Low    'MainMol' Ueq as Compared to Neighbors of         S2 Check
RESPONSE: ...
;
_vrf_PLAT260_3a
;
PROBLEM: Large Average Ueq of Residue Including      O0AA      0.105 Check
RESPONSE: ...
;
_vrf_PLAT360_3a
;
PROBLEM: Short  C(sp3)-C(sp3) Bond  C51      - C52      .       1.40 Ang.
RESPONSE: ...
;
_vrf_PLAT361_3a
;
PROBLEM: Long   C(sp3)-C(sp3) Bond  C62      - C68      .       1.74 Ang.
RESPONSE: ...
;
_vrf_PLAT362_3a
;
PROBLEM: Short  C(sp3)-C(sp2) Bond  C26      - C27      .       1.38 Ang.
RESPONSE: ...
;
_vrf_PLAT767_3a
;
PROBLEM: INS Embedded LIST 6 Instruction Should be LIST 4     Please Check
RESPONSE: ...
;
# end Validation Reply Form
```

It is advisable to attempt to resolve as many as possible of the alerts in all categories. Often the minor alerts point to easily fixed oversights, errors and omissions in your CIF or refinement strategy, so attention to these fine details can be worthwhile. In order to resolve some of the more serious problems it may be necessary to carry out additional measurements or structure refinements. However, the purpose of your study may justify the reported deviations and the more serious of these should normally be commented upon in the discussion or experimental section of a paper or in the "special_details" fields of the CIF. checkCIF was carefully designed to identify outliers and unusual parameters, but every test has its limitations and alerts that are not important in a particular case may appear. Conversely, the absence of alerts does not guarantee there are no aspects of the results needing attention. It is up to the individual to critically assess their own results and, if necessary, seek expert advice.

Publication of your CIF in IUCr journals

A basic structural check has been run on your CIF. These basic checks will be run on all CIFs submitted for publication in IUCr journals (*Acta Crystallographica*, *Journal of Applied Crystallography*, *Journal of Synchrotron Radiation*); however, if you intend to submit to *Acta Crystallographica Section C* or *E* or *IUCrData*, you should make sure that full publication checks are run on the final version of your CIF prior to submission.

Publication of your CIF in other journals

Please refer to the *Notes for Authors* of the relevant journal for any special instructions relating to CIF submission.

PLATON version of 28/11/2022; check.def file version of 28/11/2022

Datablock 3a - ellipsoid plot

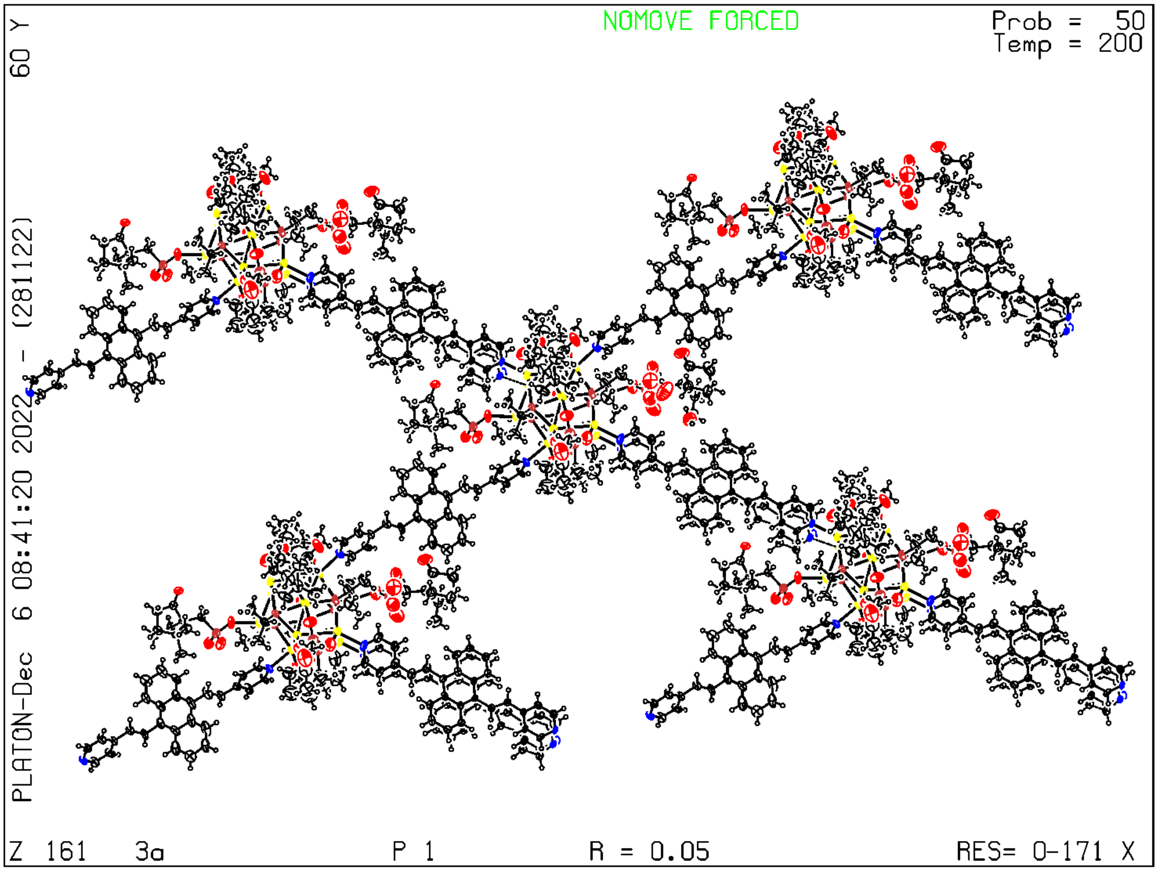

Supplement: Supplementary file 2 — Supporting Information [file ADVS-10-2207660-s002.zip › 3a-checkcif.pdf]
