# Supplementary material for: Stepwise Amplification of Circularly Polarized Luminescence in Chiral Metal Cluster Ensembles
Source: Adv Sci (Weinh). 2023 Feb 25;10(13):2207660. doi: 10.1002/advs.202207660 (PMC10161016; doi:10.1002/advs.202207660)

# checkCIF/PLATON report

You have not supplied any structure factors. As a result the full set of tests cannot be run.

THIS REPORT IS FOR GUIDANCE ONLY. IF USED AS PART OF A REVIEW PROCEDURE FOR PUBLICATION, IT SHOULD NOT REPLACE THE EXPERTISE OF AN EXPERIENCED CRYSTALLOGRAPHIC REFEREE.

No syntax errors found. CIF dictionary Interpreting this report

## Datablock: 3b

---

Bond precision: C-C = 0.0343 A Wavelength=1.54184

| | | | |
|---|---|---|---|
| Cell: | a=15.3536(3) | b=15.6214(3) | c=19.4032(3) |
| | alpha=80.377(1) | beta=81.929(1) | gamma=66.006(2) |
| Temperature: | 200 K | | |

| | Calculated | Reported |
|---|---|---|
| Volume | 4178.22(15) | 4178.22(15) |
| Space group | P 1 | P 1 |
| Hall group | P 1 | P 1 |
| Moiety formula | C161.53 H190.60 Ag12 N6 O24 S12, 2(H2 O), 0.468(C H3) | C162 H192 Ag12 N6 O24 S12, 2(H2 O) |
| Sum formula | C162 H196 Ag12 N6 O26 S12 | C162 H196 Ag12 N6 O26 S12 |
| Mr | 4322.37 | 4322.40 |
| Dx,g cm-3 | 1.718 | 1.718 |
| Z | 1 | 1 |
| Mu (mm-1) | 12.964 | 12.964 |
| F000 | 2174.0 | 2174.0 |
| F000' | 2184.60 | |
| h,k,lmax | 19,19,24 | 19,19,24 |
| Nref | 34592[ 17296] | 30451 |
| Tmin,Tmax | 0.145,0.523 | 0.007,1.000 |
| Tmin' | 0.014 | |

Correction method= # Reported T Limits: Tmin=0.007 Tmax=1.000
AbsCorr = MULTI-SCAN

Data completeness= 1.76/0.88 Theta(max)= 75.255

R(reflections)= 0.0830( 24486) wR2(reflections)= 0.2416( 30451)

S = 1.086 Npar= 2385

The following ALERTS were generated. Each ALERT has the format
**test-name_ALERT_alert-type_alert-level.**
Click on the hyperlinks for more details of the test.

## Alert level B

```
PLAT342_ALERT_3_B Low Bond Precision on  C-C Bonds ...............    0.03426 Ang.
PLAT417_ALERT_2_B Short Inter D-H..H-D       H1AB      ..H0AB     .       2.00 Ang.
                                                  x,y,z  =      1_555 Check
PLAT420_ALERT_2_B D-H Bond Without Acceptor  O0AA      --H0AB     .     Please Check
PLAT987_ALERT_1_B The Flack x is >> 0 -  Do a BASF/TWIN Refinement     Please Check
```

## Alert level C

```
PLAT042_ALERT_1_C Calc. and Reported MoietyFormula Strings  Differ     Please Check
PLAT090_ALERT_3_C Poor Data / Parameter Ratio (Zmax > 18) ........       7.25 Note
PLAT202_ALERT_3_C Isotropic non-H Atoms in Anion/Solvent .........          1 Check
              O0AA
PLAT213_ALERT_2_C Atom N3              has ADP max/min Ratio .....        3.1 prolat
PLAT213_ALERT_2_C Atom N5              has ADP max/min Ratio .....        3.4 oblate
PLAT213_ALERT_2_C Atom C58             has ADP max/min Ratio .....        3.3 oblate
PLAT220_ALERT_2_C NonSolvent   Resd 1  C   Ueq(max)/Ueq(min) Range        4.2 Ratio
PLAT220_ALERT_2_C NonSolvent   Resd 1  O   Ueq(max)/Ueq(min) Range        3.1 Ratio
PLAT222_ALERT_3_C NonSolvent Resd 1  H   Uiso(max)/Uiso(min) Range        5.2 Ratio
PLAT241_ALERT_2_C High   'MainMol' Ueq as Compared to Neighbors of        Ag9 Check
PLAT241_ALERT_2_C High   'MainMol' Ueq as Compared to Neighbors of         O1 Check
PLAT241_ALERT_2_C High   'MainMol' Ueq as Compared to Neighbors of        O10 Check
PLAT241_ALERT_2_C High   'MainMol' Ueq as Compared to Neighbors of        C10 Check
PLAT241_ALERT_2_C High   'MainMol' Ueq as Compared to Neighbors of        C17 Check
PLAT241_ALERT_2_C High   'MainMol' Ueq as Compared to Neighbors of        C40 Check
PLAT241_ALERT_2_C High   'MainMol' Ueq as Compared to Neighbors of        C71 Check
PLAT241_ALERT_2_C High   'MainMol' Ueq as Compared to Neighbors of        C77 Check
PLAT241_ALERT_2_C High   'MainMol' Ueq as Compared to Neighbors of        C87 Check
PLAT241_ALERT_2_C High   'MainMol' Ueq as Compared to Neighbors of        C90 Check
PLAT241_ALERT_2_C High   'MainMol' Ueq as Compared to Neighbors of        C96 Check
PLAT241_ALERT_2_C High   'MainMol' Ueq as Compared to Neighbors of        C97 Check
PLAT241_ALERT_2_C High   'MainMol' Ueq as Compared to Neighbors of       C124 Check
PLAT241_ALERT_2_C High   'MainMol' Ueq as Compared to Neighbors of       C158 Check
PLAT242_ALERT_2_C Low    'MainMol' Ueq as Compared to Neighbors of         S3 Check
PLAT242_ALERT_2_C Low    'MainMol' Ueq as Compared to Neighbors of         S4 Check
PLAT242_ALERT_2_C Low    'MainMol' Ueq as Compared to Neighbors of         S9 Check
PLAT242_ALERT_2_C Low    'MainMol' Ueq as Compared to Neighbors of         N2 Check
PLAT242_ALERT_2_C Low    'MainMol' Ueq as Compared to Neighbors of         N5 Check
PLAT242_ALERT_2_C Low    'MainMol' Ueq as Compared to Neighbors of        C24 Check
PLAT242_ALERT_2_C Low    'MainMol' Ueq as Compared to Neighbors of        C76 Check
PLAT242_ALERT_2_C Low    'MainMol' Ueq as Compared to Neighbors of       C118 Check
PLAT260_ALERT_2_C Large Average Ueq of Residue Including      O1AA      0.141 Check
PLAT260_ALERT_2_C Large Average Ueq of Residue Including      C189      0.104 Check
PLAT360_ALERT_2_C Short  C(sp3)-C(sp3) Bond  C92       - C103      .       1.41 Ang.
PLAT360_ALERT_2_C Short  C(sp3)-C(sp3) Bond  C103      - C121      .       1.43 Ang.
PLAT360_ALERT_2_C Short  C(sp3)-C(sp3) Bond  C109      - C123      .       1.40 Ang.
PLAT369_ALERT_2_C Long   C(sp2)-C(sp2) Bond  C71       - C76       .       1.53 Ang.
PLAT369_ALERT_2_C Long   C(sp2)-C(sp2) Bond  C113      - C114      .       1.53 Ang.
PLAT410_ALERT_2_C Short Intra H...H Contact  H12       ..H14       .       1.99 Ang.
                                                  x,y,z  =      1_555 Check
PLAT767_ALERT_4_C INS Embedded LIST 6 Instruction Should be LIST 4     Please Check
```

## Alert level G

```
PLAT002_ALERT_2_G Number of Distance or Angle Restraints on AtSite        141 Note
PLAT003_ALERT_2_G Number of Uiso or Uij Restrained non-H Atoms ...        160 Report
PLAT004_ALERT_5_G Polymeric Structure Found with Maximum Dimension          2 Info
PLAT007_ALERT_5_G Number of Unrefined Donor-H Atoms .............           4 Report
PLAT033_ALERT_4_G Flack x Value Deviates > 3.0 * sigma from Zero .      0.190 Note
PLAT072_ALERT_2_G SHELXL First  Parameter in WGHT  Unusually Large       0.14 Report
PLAT083_ALERT_2_G SHELXL Second Parameter in WGHT  Unusually Large      14.38 Why ?
PLAT172_ALERT_4_G The CIF-Embedded .res File Contains DFIX Records         51 Report
PLAT173_ALERT_4_G The CIF-Embedded .res File Contains DANG Records         26 Report
PLAT174_ALERT_4_G The CIF-Embedded .res File Contains FLAT Records          1 Report
PLAT176_ALERT_4_G The CIF-Embedded .res File Contains SADI Records          6 Report
PLAT177_ALERT_4_G The CIF-Embedded .res File Contains DELU Records          6 Report
PLAT178_ALERT_4_G The CIF-Embedded .res File Contains SIMU Records          7 Report
PLAT186_ALERT_4_G The CIF-Embedded .res File Contains ISOR Records         29 Report
PLAT188_ALERT_3_G A Non-default SIMU Restraint Value has been used     0.0100 Report
PLAT188_ALERT_3_G A Non-default SIMU Restraint Value has been used     0.0100 Report
PLAT188_ALERT_3_G A Non-default SIMU Restraint Value has been used     0.0100 Report
PLAT188_ALERT_3_G A Non-default SIMU Restraint Value has been used     0.0100 Report
PLAT188_ALERT_3_G A Non-default SIMU Restraint Value has been used     0.0100 Report
PLAT188_ALERT_3_G A Non-default SIMU Restraint Value has been used     0.0100 Report
PLAT188_ALERT_3_G A Non-default SIMU Restraint Value has been used     0.0100 Report
PLAT300_ALERT_4_G Atom Site Occupancy of S13       Constrained at         0.5 Check
PLAT300_ALERT_4_G Atom Site Occupancy of S14       Constrained at         0.5 Check
PLAT300_ALERT_4_G Atom Site Occupancy of O24       Constrained at         0.5 Check
PLAT300_ALERT_4_G Atom Site Occupancy of O25       Constrained at         0.5 Check
PLAT300_ALERT_4_G Atom Site Occupancy of O26       Constrained at         0.5 Check
PLAT300_ALERT_4_G Atom Site Occupancy of O27       Constrained at         0.5 Check
PLAT300_ALERT_4_G Atom Site Occupancy of O29       Constrained at         0.5 Check
PLAT300_ALERT_4_G Atom Site Occupancy of O30       Constrained at         0.5 Check
PLAT300_ALERT_4_G Atom Site Occupancy of O31       Constrained at         0.5 Check
PLAT300_ALERT_4_G Atom Site Occupancy of O32       Constrained at         0.5 Check
PLAT300_ALERT_4_G Atom Site Occupancy of O33       Constrained at         0.5 Check
PLAT300_ALERT_4_G Atom Site Occupancy of O34       Constrained at         0.5 Check
PLAT300_ALERT_4_G Atom Site Occupancy of O35       Constrained at         0.5 Check
PLAT300_ALERT_4_G Atom Site Occupancy of O36       Constrained at         0.5 Check
PLAT300_ALERT_4_G Atom Site Occupancy of C73       Constrained at         0.5 Check
PLAT300_ALERT_4_G Atom Site Occupancy of C74       Constrained at         0.5 Check
PLAT300_ALERT_4_G Atom Site Occupancy of C75       Constrained at         0.5 Check
PLAT300_ALERT_4_G Atom Site Occupancy of C129      Constrained at         0.5 Check
PLAT300_ALERT_4_G Atom Site Occupancy of C130      Constrained at         0.5 Check
PLAT300_ALERT_4_G Atom Site Occupancy of C131      Constrained at         0.5 Check
PLAT300_ALERT_4_G Atom Site Occupancy of C132      Constrained at         0.5 Check
PLAT300_ALERT_4_G Atom Site Occupancy of C133      Constrained at         0.5 Check
PLAT300_ALERT_4_G Atom Site Occupancy of C134      Constrained at         0.5 Check
PLAT300_ALERT_4_G Atom Site Occupancy of C135      Constrained at         0.5 Check
PLAT300_ALERT_4_G Atom Site Occupancy of C136      Constrained at         0.5 Check
PLAT300_ALERT_4_G Atom Site Occupancy of C137      Constrained at         0.5 Check
PLAT300_ALERT_4_G Atom Site Occupancy of C166      Constrained at         0.5 Check
PLAT300_ALERT_4_G Atom Site Occupancy of C167      Constrained at         0.5 Check
PLAT300_ALERT_4_G Atom Site Occupancy of C168      Constrained at         0.5 Check
PLAT300_ALERT_4_G Atom Site Occupancy of C169      Constrained at         0.5 Check
PLAT300_ALERT_4_G Atom Site Occupancy of C170      Constrained at         0.5 Check
PLAT300_ALERT_4_G Atom Site Occupancy of C171      Constrained at         0.5 Check
PLAT300_ALERT_4_G Atom Site Occupancy of C172      Constrained at         0.5 Check
PLAT300_ALERT_4_G Atom Site Occupancy of C173      Constrained at         0.5 Check
PLAT300_ALERT_4_G Atom Site Occupancy of C174      Constrained at         0.5 Check
```

```
PLAT300_ALERT_4_G Atom Site Occupancy of C175       Constrained at        0.5 Check
PLAT300_ALERT_4_G Atom Site Occupancy of C176       Constrained at        0.5 Check
PLAT300_ALERT_4_G Atom Site Occupancy of C177       Constrained at        0.5 Check
PLAT300_ALERT_4_G Atom Site Occupancy of C178       Constrained at        0.5 Check
PLAT300_ALERT_4_G Atom Site Occupancy of C179       Constrained at        0.5 Check
PLAT300_ALERT_4_G Atom Site Occupancy of C180       Constrained at        0.5 Check
PLAT300_ALERT_4_G Atom Site Occupancy of C181       Constrained at        0.5 Check
PLAT300_ALERT_4_G Atom Site Occupancy of C182       Constrained at        0.5 Check
PLAT300_ALERT_4_G Atom Site Occupancy of C183       Constrained at        0.5 Check
PLAT300_ALERT_4_G Atom Site Occupancy of C184       Constrained at        0.5 Check
PLAT300_ALERT_4_G Atom Site Occupancy of C185       Constrained at        0.5 Check
PLAT300_ALERT_4_G Atom Site Occupancy of H16I       Constrained at        0.5 Check
PLAT300_ALERT_4_G Atom Site Occupancy of H16J       Constrained at        0.5 Check
PLAT300_ALERT_4_G Atom Site Occupancy of H16K       Constrained at        0.5 Check
PLAT300_ALERT_4_G Atom Site Occupancy of H16L       Constrained at        0.5 Check
PLAT300_ALERT_4_G Atom Site Occupancy of H17A       Constrained at        0.5 Check
PLAT300_ALERT_4_G Atom Site Occupancy of H17B       Constrained at        0.5 Check
PLAT300_ALERT_4_G Atom Site Occupancy of H17C       Constrained at        0.5 Check
PLAT300_ALERT_4_G Atom Site Occupancy of H17D       Constrained at        0.5 Check
PLAT300_ALERT_4_G Atom Site Occupancy of H17E       Constrained at        0.5 Check
PLAT300_ALERT_4_G Atom Site Occupancy of H17F       Constrained at        0.5 Check
PLAT300_ALERT_4_G Atom Site Occupancy of H17G       Constrained at        0.5 Check
PLAT300_ALERT_4_G Atom Site Occupancy of H17H       Constrained at        0.5 Check
PLAT300_ALERT_4_G Atom Site Occupancy of H17I       Constrained at        0.5 Check
PLAT300_ALERT_4_G Atom Site Occupancy of H17J       Constrained at        0.5 Check
PLAT300_ALERT_4_G Atom Site Occupancy of H17K       Constrained at        0.5 Check
PLAT300_ALERT_4_G Atom Site Occupancy of H17L       Constrained at        0.5 Check
PLAT300_ALERT_4_G Atom Site Occupancy of H17M       Constrained at        0.5 Check
PLAT300_ALERT_4_G Atom Site Occupancy of H17N       Constrained at        0.5 Check
PLAT300_ALERT_4_G Atom Site Occupancy of H18A       Constrained at        0.5 Check
PLAT300_ALERT_4_G Atom Site Occupancy of H18B       Constrained at        0.5 Check
PLAT300_ALERT_4_G Atom Site Occupancy of H18C       Constrained at        0.5 Check
PLAT300_ALERT_4_G Atom Site Occupancy of H18D       Constrained at        0.5 Check
PLAT300_ALERT_4_G Atom Site Occupancy of H18E       Constrained at        0.5 Check
PLAT300_ALERT_4_G Atom Site Occupancy of H18F       Constrained at        0.5 Check
PLAT300_ALERT_4_G Atom Site Occupancy of H18G       Constrained at        0.5 Check
PLAT300_ALERT_4_G Atom Site Occupancy of H18H       Constrained at        0.5 Check
PLAT300_ALERT_4_G Atom Site Occupancy of H18I       Constrained at        0.5 Check
PLAT300_ALERT_4_G Atom Site Occupancy of H18J       Constrained at        0.5 Check
PLAT300_ALERT_4_G Atom Site Occupancy of H31        Constrained at        0.5 Check
PLAT300_ALERT_4_G Atom Site Occupancy of H31A       Constrained at        0.5 Check
PLAT300_ALERT_4_G Atom Site Occupancy of H74        Constrained at        0.5 Check
PLAT300_ALERT_4_G Atom Site Occupancy of H75        Constrained at        0.5 Check
PLAT300_ALERT_4_G Atom Site Occupancy of H129       Constrained at        0.5 Check
PLAT300_ALERT_4_G Atom Site Occupancy of H130       Constrained at        0.5 Check
PLAT300_ALERT_4_G Atom Site Occupancy of H131       Constrained at        0.5 Check
PLAT300_ALERT_4_G Atom Site Occupancy of H132       Constrained at        0.5 Check
PLAT300_ALERT_4_G Atom Site Occupancy of H133       Constrained at        0.5 Check
PLAT300_ALERT_4_G Atom Site Occupancy of H134       Constrained at        0.5 Check
PLAT300_ALERT_4_G Atom Site Occupancy of H136       Constrained at        0.5 Check
PLAT300_ALERT_4_G Atom Site Occupancy of H137       Constrained at        0.5 Check
PLAT300_ALERT_4_G Atom Site Occupancy of H172       Constrained at        0.5 Check
PLAT300_ALERT_4_G Atom Site Occupancy of H182       Constrained at        0.5 Check
PLAT301_ALERT_3_G Main Residue  Disorder .............(Resd  1  )        23% Note
PLAT302_ALERT_4_G Anion/Solvent/Minor-Residue Disorder (Resd  4  )       100% Note
PLAT343_ALERT_2_G Unusual sp3      Angle Range in Main Residue for        C35 Check
PLAT343_ALERT_2_G Unusual sp3      Angle Range in Main Residue for       C141 Check
```

```
PLAT410_ALERT_2_G Short Intra H...H Contact  H10      ..H129     .       2.10 Ang.
                                                        x,y,z  =      1_555 Check
PLAT410_ALERT_2_G Short Intra H...H Contact  H31      ..H68      .       2.09 Ang.
                                                        x,y,z  =      1_555 Check
PLAT410_ALERT_2_G Short Intra H...H Contact  H105     ..H133     .       2.11 Ang.
                                                        x,y,z  =      1_555 Check
PLAT413_ALERT_2_G Short Inter XH3 .. XHn     H12K     ..H51B     .       1.56 Ang.
                                                     x,-1+y,z  =      1_545 Check
PLAT413_ALERT_2_G Short Inter XH3 .. XHn     H12K     ..H51C     .       1.91 Ang.
                                                     x,-1+y,z  =      1_545 Check
PLAT413_ALERT_2_G Short Inter XH3 .. XHn     H69B     ..H15F     .       2.13 Ang.
                                                     -1+x,y,z  =      1_455 Check
PLAT720_ALERT_4_G Number of Unusual/Non-Standard Labels ..........          6 Note
PLAT721_ALERT_1_G Bond    Calc     0.97000, Rep     0.96000 Dev...       0.01 Ang.
              C100     -H10B           1_555   1_555 ........     #  343 Check
PLAT721_ALERT_1_G Bond    Calc     0.95000, Rep     0.96020 Dev...       0.01 Ang.
              C100     -H10C           1_555   1_555 ........     #  344 Check
PLAT721_ALERT_1_G Bond    Calc     0.95000, Rep     0.96010 Dev...       0.01 Ang.
              C145     -H14G           1_555   1_555 ........     #  438 Check
PLAT722_ALERT_1_G Angle   Calc      113.00, Rep      111.80 Dev...       1.20 Degree
              C181    -C180    -H18B       1_555  1_555  1_555     # 1151 Check
PLAT722_ALERT_1_G Angle   Calc      107.00, Rep      108.10 Dev...       1.10 Degree
              H18C    -C181    -H18D       1_555  1_555  1_555     # 1155 Check
PLAT722_ALERT_1_G Angle   Calc      111.00, Rep      112.30 Dev...       1.30 Degree
              C196    -C193    -H19B       1_555  1_555  1_555     # 1217 Check
PLAT722_ALERT_1_G Angle   Calc      111.00, Rep      112.20 Dev...       1.20 Degree
              C191    -C197    -H19L       1_555  1_555  1_555     # 1240 Check
PLAT773_ALERT_2_G Check long C-C Bond in CIF: C159     --C162            1.71 Ang.
PLAT773_ALERT_2_G Check long C-C Bond in CIF: C162     --C189            1.85 Ang.
PLAT860_ALERT_3_G Number of Least-Squares Restraints ............       1906 Note
```

---

   0 **ALERT level A** = Most likely a serious problem - resolve or explain
   4 **ALERT level B** = A potentially serious problem, consider carefully
  40 **ALERT level C** = Check. Ensure it is not caused by an omission or oversight
 130 **ALERT level G** = General information/check it is not something unexpected

```
   9 ALERT type 1 CIF construction/syntax error, inconsistent or missing data
  51 ALERT type 2 Indicator that the structure model may be wrong or deficient
  13 ALERT type 3 Indicator that the structure quality may be low
  99 ALERT type 4 Improvement, methodology, query or suggestion
   2 ALERT type 5 Informative message, check
```

---

### Validation response form

Please find below a validation response form (VRF) that can be filled in and pasted into your CIF.

```
# start Validation Reply Form
_vrf_PLAT342_3b
;
PROBLEM: Low Bond Precision on  C-C Bonds ..............    0.03426 Ang.
RESPONSE: ...
;
_vrf_PLAT417_3b
;
PROBLEM: Short Inter D-H..H-D       H1AB     ..H0AB     .       2.00 Ang.
```

```
RESPONSE: ...
;
_vrf_PLAT420_3b
;
PROBLEM: D-H Bond Without Acceptor  O0AA     --H0AB     .     Please Check
RESPONSE: ...
;
_vrf_PLAT987_3b
;
PROBLEM: The Flack x is >> 0 -  Do a BASF/TWIN Refinement     Please Check
RESPONSE: ...
;
_vrf_PLAT042_3b
;
PROBLEM: Calc. and Reported MoietyFormula Strings  Differ     Please Check
RESPONSE: ...
;
_vrf_PLAT090_3b
;
PROBLEM: Poor Data / Parameter Ratio (Zmax > 18) ........       7.25 Note
RESPONSE: ...
;
_vrf_PLAT202_3b
;
PROBLEM: Isotropic non-H Atoms in Anion/Solvent .........          1 Check
RESPONSE: ...
;
_vrf_PLAT213_3b
;
PROBLEM: Atom N3              has ADP max/min Ratio .....        3.1 prolat
RESPONSE: ...
;
_vrf_PLAT220_3b
;
PROBLEM: NonSolvent   Resd 1  C   Ueq(max)/Ueq(min) Range        4.2 Ratio
RESPONSE: ...
;
_vrf_PLAT222_3b
;
PROBLEM: NonSolvent Resd 1  H   Uiso(max)/Uiso(min) Range        5.2 Ratio
RESPONSE: ...
;
_vrf_PLAT241_3b
;
PROBLEM: High   'MainMol' Ueq as Compared to Neighbors of        Ag9 Check
RESPONSE: ...
;
_vrf_PLAT242_3b
;
PROBLEM: Low    'MainMol' Ueq as Compared to Neighbors of         S3 Check
RESPONSE: ...
;
_vrf_PLAT260_3b
;
PROBLEM: Large Average Ueq of Residue Including      O1AA      0.141 Check
RESPONSE: ...
;
```

```
_vrf_PLAT360_3b
;
PROBLEM: Short  C(sp3)-C(sp3) Bond  C92      - C103     .       1.41 Ang.
RESPONSE: ...
;
_vrf_PLAT369_3b
;
PROBLEM: Long   C(sp2)-C(sp2) Bond  C71      - C76      .       1.53 Ang.
RESPONSE: ...
;
_vrf_PLAT410_3b
;
PROBLEM: Short Intra H...H Contact  H12      ..H14      .       1.99 Ang.
RESPONSE: ...
;
_vrf_PLAT767_3b
;
PROBLEM: INS Embedded LIST 6 Instruction Should be LIST 4     Please Check
RESPONSE: ...
;
# end Validation Reply Form
```

---

It is advisable to attempt to resolve as many as possible of the alerts in all categories. Often the minor alerts point to easily fixed oversights, errors and omissions in your CIF or refinement strategy, so attention to these fine details can be worthwhile. In order to resolve some of the more serious problems it may be necessary to carry out additional measurements or structure refinements. However, the purpose of your study may justify the reported deviations and the more serious of these should normally be commented upon in the discussion or experimental section of a paper or in the "special_details" fields of the CIF. checkCIF was carefully designed to identify outliers and unusual parameters, but every test has its limitations and alerts that are not important in a particular case may appear. Conversely, the absence of alerts does not guarantee there are no aspects of the results needing attention. It is up to the individual to critically assess their own results and, if necessary, seek expert advice.

**Publication of your CIF in IUCr journals**

A basic structural check has been run on your CIF. These basic checks will be run on all CIFs submitted for publication in IUCr journals (*Acta Crystallographica*, *Journal of Applied Crystallography*, *Journal of Synchrotron Radiation*); however, if you intend to submit to *Acta Crystallographica Section C* or *E* or *IUCrData*, you should make sure that full publication checks are run on the final version of your CIF prior to submission.

**Publication of your CIF in other journals**

Please refer to the *Notes for Authors* of the relevant journal for any special instructions relating to CIF submission.

---

**PLATON version of 28/11/2022; check.def file version of 28/11/2022**

**Datablock 3b** - ellipsoid plot

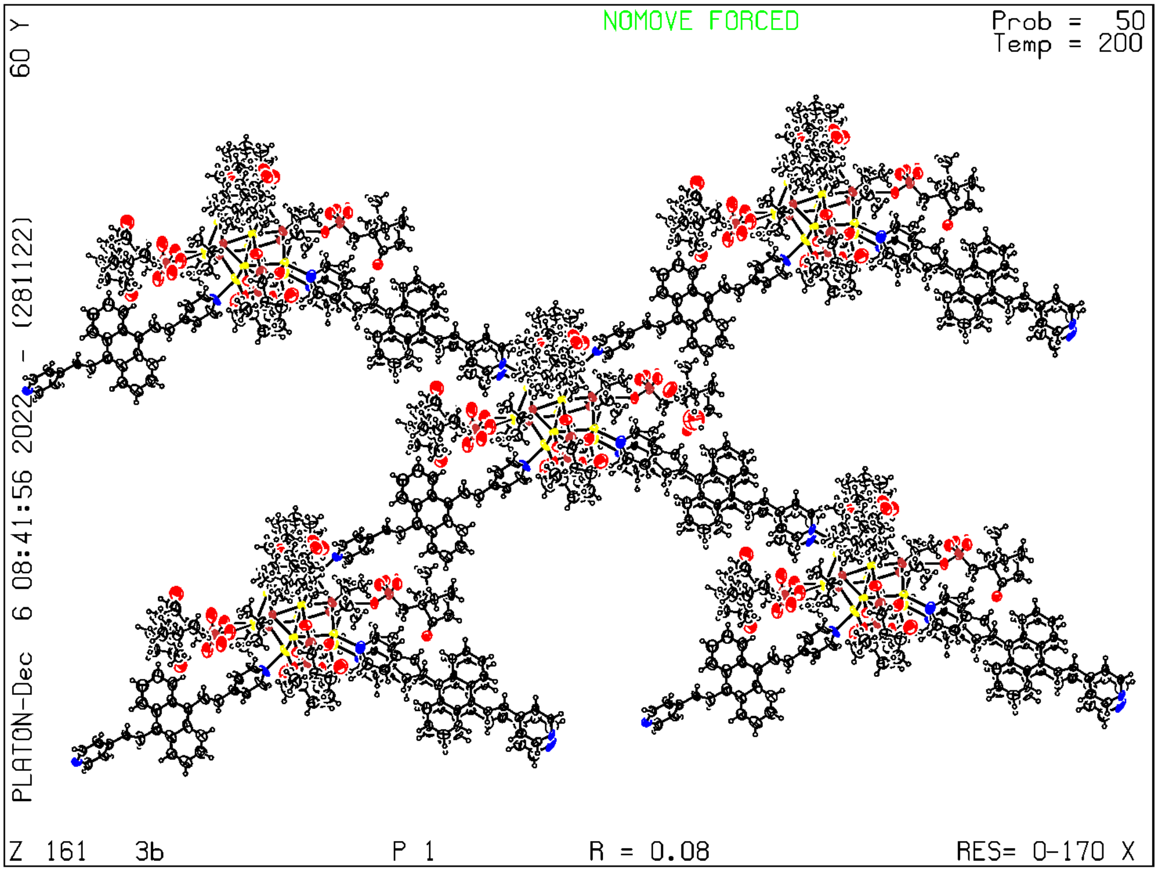

Supplement: Supplementary file 2 — Supporting Information [file ADVS-10-2207660-s002.zip › 3b-checkcif.pdf]
