# Supplementary material for: Stepwise Amplification of Circularly Polarized Luminescence in Chiral Metal Cluster Ensembles
Source: Adv Sci (Weinh). 2023 Feb 25;10(13):2207660. doi: 10.1002/advs.202207660 (PMC10161016; doi:10.1002/advs.202207660)

checkCIF/PLATON report

You have not supplied any structure factors. As a result the full set of tests cannot be run.

THIS REPORT IS FOR GUIDANCE ONLY. IF USED AS PART OF A REVIEW PROCEDURE FOR PUBLICATION, IT SHOULD NOT REPLACE THE EXPERTISE OF AN EXPERIENCED CRYSTALLOGRAPHIC REFEREE.

No syntax errors found. CIF dictionary Interpreting this report

Datablock: 4a

```
Bond precision:   C-C = 0.0435 A          Wavelength=1.54184

Cell:           a=15.8156(2)      b=17.8033(3)      c=17.9216(2)
                alpha=96.569(1)   beta=97.437(1)    gamma=116.084(1)
Temperature:    200 K
```

	Calculated	Reported
Volume	4410.18(11)	4410.17(11)
Space group	P 1	P 1
Hall group	P 1	P 1
Moiety formula	C98 H162 Ag12 O32 S14, C28 H22 N2, 3(C H Cl3), C3 H6 O [+ solve	C98 H162 Ag12 O32 S14, 3(C H Cl3), C28 H22 N2, C3 H6 O
Sum formula	C132 H193 Ag12 Cl9 N2 O33 S14 [+ solvent]	C132 H193 Ag12 Cl9 N2 O33 S14
Mr	4398.23	4398.20
Dx,g cm-3	1.656	1.656
Z	1	1
Mu (mm-1)	13.753	13.753
F000	2204.0	2204.0
F000'	2218.46	
h,k,lmax	19,22,22	19,21,22
Nref	35766[17883]	21234
Tmin,Tmax	0.092,0.503	0.057,1.000
Tmin'	0.006	

```
Correction method= # Reported T Limits: Tmin=0.057 Tmax=1.000
AbsCorr = MULTI-SCAN

Data completeness= 1.19/0.59       Theta(max)= 73.891
```

R(reflections)= 0.0842(17685) wR2(reflections)= 0.2389(21234)

S = 0.917 Npar= 1894

The following ALERTS were generated. Each ALERT has the format
test-name_ALERT_alert-type_alert-level.
Click on the hyperlinks for more details of the test.

Alert level B

```
PLAT342_ALERT_3_B Low Bond Precision on  C-C Bonds ...............    0.04348 Ang.
PLAT987_ALERT_1_B The Flack x is >> 0 -  Do a BASF/TWIN Refinement     Please Check
```

Alert level C

```
SHFSU01_ALERT_2_C  The absolute value of parameter shift to su ratio > 0.05
            Absolute value of the parameter shift to su ratio given   0.075
            Additional refinement cycles may be required.
PLAT080_ALERT_2_C Maximum Shift/Error ...........................      0.08 Why ?
PLAT213_ALERT_2_C Atom O4               has ADP max/min Ratio .....     3.3 oblate
PLAT220_ALERT_2_C NonSolvent   Resd 1  C   Ueq(max)/Ueq(min) Range      4.4 Ratio
PLAT222_ALERT_3_C NonSolvent Resd 1  H   Uiso(max)/Uiso(min) Range      4.6 Ratio
PLAT234_ALERT_4_C Large Hirshfeld Difference S2       --C14      .      0.19 Ang.
PLAT234_ALERT_4_C Large Hirshfeld Difference S13      --O21      .      0.21 Ang.
PLAT234_ALERT_4_C Large Hirshfeld Difference O15      --C62      .      0.21 Ang.
PLAT234_ALERT_4_C Large Hirshfeld Difference O25      --C22      .      0.23 Ang.
PLAT234_ALERT_4_C Large Hirshfeld Difference C45      --C124     .      0.22 Ang.
PLAT234_ALERT_4_C Large Hirshfeld Difference C90      --C93      .      0.24 Ang.
PLAT234_ALERT_4_C Large Hirshfeld Difference N1       --C81      .      0.17 Ang.
PLAT234_ALERT_4_C Large Hirshfeld Difference C29      --C42      .      0.21 Ang.
PLAT241_ALERT_2_C High   'MainMol' Ueq as Compared to Neighbors of      O14 Check
PLAT241_ALERT_2_C High   'MainMol' Ueq as Compared to Neighbors of      O18 Check
PLAT241_ALERT_2_C High   'MainMol' Ueq as Compared to Neighbors of      O19 Check
PLAT241_ALERT_2_C High   'MainMol' Ueq as Compared to Neighbors of      O21 Check
PLAT241_ALERT_2_C High   'MainMol' Ueq as Compared to Neighbors of      O22 Check
PLAT241_ALERT_2_C High   'MainMol' Ueq as Compared to Neighbors of      O23 Check
PLAT241_ALERT_2_C High   'MainMol' Ueq as Compared to Neighbors of      O26 Check
PLAT241_ALERT_2_C High   'MainMol' Ueq as Compared to Neighbors of      O29 Check
PLAT241_ALERT_2_C High   'MainMol' Ueq as Compared to Neighbors of     C0AA Check
PLAT241_ALERT_2_C High   'MainMol' Ueq as Compared to Neighbors of      C33 Check
PLAT241_ALERT_2_C High   'MainMol' Ueq as Compared to Neighbors of      C90 Check
PLAT241_ALERT_2_C High   'MainMol' Ueq as Compared to Neighbors of      C97 Check
PLAT241_ALERT_2_C High   'MainMol' Ueq as Compared to Neighbors of     C100 Check
PLAT241_ALERT_2_C High   'MainMol' Ueq as Compared to Neighbors of     C121 Check
PLAT241_ALERT_2_C High   'MainMol' Ueq as Compared to Neighbors of     C124 Check
PLAT241_ALERT_2_C High   'MainMol' Ueq as Compared to Neighbors of      C47 Check
PLAT241_ALERT_2_C High   'MainMol' Ueq as Compared to Neighbors of      C68 Check
PLAT242_ALERT_2_C Low    'MainMol' Ueq as Compared to Neighbors of      C10 Check
PLAT242_ALERT_2_C Low    'MainMol' Ueq as Compared to Neighbors of      C22 Check
PLAT242_ALERT_2_C Low    'MainMol' Ueq as Compared to Neighbors of      C27 Check
PLAT242_ALERT_2_C Low    'MainMol' Ueq as Compared to Neighbors of      C38 Check
PLAT242_ALERT_2_C Low    'MainMol' Ueq as Compared to Neighbors of      C45 Check
PLAT242_ALERT_2_C Low    'MainMol' Ueq as Compared to Neighbors of      C93 Check
PLAT242_ALERT_2_C Low    'MainMol' Ueq as Compared to Neighbors of      C95 Check
PLAT242_ALERT_2_C Low    'MainMol' Ueq as Compared to Neighbors of     C105 Check
```

```
PLAT242_ALERT_2_C Low    'MainMol' Ueq as Compared to Neighbors of        C21 Check
PLAT242_ALERT_2_C Low    'MainMol' Ueq as Compared to Neighbors of        C46 Check
PLAT243_ALERT_4_C High   'Solvent' Ueq as Compared to Neighbors of       C130 Check
PLAT244_ALERT_4_C Low    'Solvent' Ueq as Compared to Neighbors of        C19 Check
PLAT244_ALERT_4_C Low    'Solvent' Ueq as Compared to Neighbors of       C125 Check
PLAT260_ALERT_2_C Large Average Ueq of Residue Including       Cl1      0.104 Check
PLAT260_ALERT_2_C Large Average Ueq of Residue Including       Cl4      0.165 Check
PLAT260_ALERT_2_C Large Average Ueq of Residue Including       Cl7      0.154 Check
PLAT260_ALERT_2_C Large Average Ueq of Residue Including      Cl10      0.115 Check
PLAT260_ALERT_2_C Large Average Ueq of Residue Including       O33      0.176 Check
PLAT360_ALERT_2_C Short  C(sp3)-C(sp3) Bond  C48      - C78      .       1.42 Ang.
PLAT360_ALERT_2_C Short  C(sp3)-C(sp3) Bond  C71      - C96      .       1.42 Ang.
PLAT360_ALERT_2_C Short  C(sp3)-C(sp3) Bond  C95      - C115     .       1.42 Ang.
PLAT360_ALERT_2_C Short  C(sp3)-C(sp3) Bond  C117     - C119     .       1.43 Ang.
PLAT361_ALERT_2_C Long   C(sp3)-C(sp3) Bond  C90      - C124     .       1.69 Ang.
PLAT363_ALERT_2_C Long   C(sp3)-C(sp2) Bond  C58      - C112     .       1.63 Ang.
PLAT410_ALERT_2_C Short Intra H...H Contact  H12F     ..H97B     .       1.99 Ang.
                                                      x,y,z  =      1_555 Check
PLAT767_ALERT_4_C INS Embedded LIST 6 Instruction Should be LIST 4     Please Check
```

Alert level G

```
PLAT002_ALERT_2_G Number of Distance or Angle Restraints on AtSite         34 Note
PLAT003_ALERT_2_G Number of Uiso or Uij Restrained non-H Atoms ...         82 Report
PLAT007_ALERT_5_G Number of Unrefined Donor-H Atoms .............          2 Report
PLAT033_ALERT_4_G Flack x Value Deviates > 3.0 * sigma from Zero .      0.089 Note
PLAT042_ALERT_1_G Calc. and Reported MoietyFormula Strings  Differ     Please Check
PLAT072_ALERT_2_G SHELXL First  Parameter in WGHT  Unusually Large       0.18 Report
PLAT083_ALERT_2_G SHELXL Second Parameter in WGHT  Unusually Large      25.34 Why ?
PLAT154_ALERT_1_G The s.u.'s on the Cell Angles are Equal ..(Note)      0.001 Degree
PLAT172_ALERT_4_G The CIF-Embedded .res File Contains DFIX Records         13 Report
PLAT173_ALERT_4_G The CIF-Embedded .res File Contains DANG Records          3 Report
PLAT176_ALERT_4_G The CIF-Embedded .res File Contains SADI Records          1 Report
PLAT177_ALERT_4_G The CIF-Embedded .res File Contains DELU Records          5 Report
PLAT178_ALERT_4_G The CIF-Embedded .res File Contains SIMU Records          7 Report
PLAT186_ALERT_4_G The CIF-Embedded .res File Contains ISOR Records         20 Report
PLAT188_ALERT_3_G A Non-default SIMU Restraint Value has been used     0.0100 Report
PLAT188_ALERT_3_G A Non-default SIMU Restraint Value has been used     0.0100 Report
PLAT188_ALERT_3_G A Non-default SIMU Restraint Value has been used     0.0100 Report
PLAT188_ALERT_3_G A Non-default SIMU Restraint Value has been used     0.0100 Report
PLAT188_ALERT_3_G A Non-default SIMU Restraint Value has been used     0.0100 Report
PLAT188_ALERT_3_G A Non-default SIMU Restraint Value has been used     0.0100 Report
PLAT188_ALERT_3_G A Non-default SIMU Restraint Value has been used     0.0100 Report
PLAT232_ALERT_2_G Hirshfeld Test Diff (M-X)  Ag2      --S10      .        6.0 s.u.
PLAT232_ALERT_2_G Hirshfeld Test Diff (M-X)  Ag4      --S5       .        8.7 s.u.
PLAT232_ALERT_2_G Hirshfeld Test Diff (M-X)  Ag11     --S6       .        6.0 s.u.
PLAT300_ALERT_4_G Atom Site Occupancy of Cl7        Constrained at        0.5 Check
PLAT300_ALERT_4_G Atom Site Occupancy of Cl8        Constrained at        0.5 Check
PLAT300_ALERT_4_G Atom Site Occupancy of Cl9        Constrained at        0.5 Check
PLAT300_ALERT_4_G Atom Site Occupancy of C131       Constrained at        0.5 Check
PLAT300_ALERT_4_G Atom Site Occupancy of H131       Constrained at        0.5 Check
PLAT300_ALERT_4_G Atom Site Occupancy of Cl10       Constrained at        0.5 Check
PLAT300_ALERT_4_G Atom Site Occupancy of Cl11       Constrained at        0.5 Check
PLAT300_ALERT_4_G Atom Site Occupancy of Cl12       Constrained at        0.5 Check
PLAT300_ALERT_4_G Atom Site Occupancy of C132       Constrained at        0.5 Check
PLAT300_ALERT_4_G Atom Site Occupancy of H132       Constrained at        0.5 Check
PLAT301_ALERT_3_G Main Residue  Disorder .............(Resd  1  )         1% Note
```

```
PLAT302_ALERT_4_G Anion/Solvent/Minor-Residue Disorder (Resd  5  )       100% Note
PLAT302_ALERT_4_G Anion/Solvent/Minor-Residue Disorder (Resd  6  )       100% Note
PLAT303_ALERT_2_G Full Occupancy Atom H10C      with # Connections       1.27 Check
PLAT304_ALERT_4_G Non-Integer Number of Atoms in ..... (Resd  5  )       2.50 Check
PLAT304_ALERT_4_G Non-Integer Number of Atoms in ..... (Resd  6  )       2.50 Check
PLAT335_ALERT_2_G Check Large C6 Ring C-C Range C4       -C29            0.17 Ang.
PLAT343_ALERT_2_G Unusual sp3      Angle Range in Main Residue for        C20 Check
PLAT343_ALERT_2_G Unusual sp3      Angle Range in Main Residue for        C57 Check
PLAT431_ALERT_2_G Short Inter HL..A Contact  Cl2      ..O11      .       3.00 Ang.
                                                -1+x,y,z  =      1_455 Check
PLAT431_ALERT_2_G Short Inter HL..A Contact  Cl4      ..O27      .       2.98 Ang.
                                                 1+x,y,z  =      1_655 Check
PLAT606_ALERT_4_G Solvent Accessible VOID(S) in Structure ........          ! Info
PLAT720_ALERT_4_G Number of Unusual/Non-Standard Labels ..........          3 Note
PLAT721_ALERT_1_G Bond    Calc     0.95000, Rep     0.96010 Dev...       0.01 Ang.
              C127     -H12J           1_555   1_555 ........     #  436 Check
PLAT722_ALERT_1_G Angle   Calc      108.00, Rep      109.50 Dev...       1.50 Degree
              H3B     -C3      -H3C        1_555  1_555  1_555     #  346 Check
PLAT722_ALERT_1_G Angle   Calc      112.00, Rep      113.30 Dev...       1.30 Degree
              C72     -C7      -H7         1_555  1_555  1_555     #  366 Check
PLAT722_ALERT_1_G Angle   Calc      113.00, Rep      111.80 Dev...       1.20 Degree
              C7      -C88     -H88B       1_555  1_555  1_555     #  780 Check
PLAT722_ALERT_1_G Angle   Calc      111.00, Rep      112.20 Dev...       1.20 Degree
              C122    -C107    -H10I       1_555  1_555  1_555     #  890 Check
PLAT722_ALERT_1_G Angle   Calc      120.00, Rep      118.60 Dev...       1.40 Degree
              C70     -C108    -H108       1_555  1_555  1_555     #  891 Check
PLAT722_ALERT_1_G Angle   Calc       81.00, Rep       79.80 Dev...       1.20 Degree
              C130    -C127    -H12J       1_555  1_555  1_555     # 1006 Check
PLAT790_ALERT_4_G Centre of Gravity not Within Unit Cell: Resd.  #          2 Note
              C28 H22 N2
PLAT790_ALERT_4_G Centre of Gravity not Within Unit Cell: Resd.  #          6 Note
              C H Cl3
PLAT791_ALERT_4_G Model has Chirality at C7         (Sohnke SpGr)          R Verify
PLAT791_ALERT_4_G Model has Chirality at C9         (Sohnke SpGr)          R Verify
PLAT791_ALERT_4_G Model has Chirality at C10        (Sohnke SpGr)          S Verify
PLAT791_ALERT_4_G Model has Chirality at C16        (Sohnke SpGr)          S Verify
PLAT791_ALERT_4_G Model has Chirality at C20        (Sohnke SpGr)          S Verify
PLAT791_ALERT_4_G Model has Chirality at C30        (Sohnke SpGr)          S Verify
PLAT791_ALERT_4_G Model has Chirality at C38        (Sohnke SpGr)          R Verify
PLAT791_ALERT_4_G Model has Chirality at C44        (Sohnke SpGr)          S Verify
PLAT791_ALERT_4_G Model has Chirality at C57        (Sohnke SpGr)          S Verify
PLAT791_ALERT_4_G Model has Chirality at C67        (Sohnke SpGr)          R Verify
PLAT791_ALERT_4_G Model has Chirality at C90        (Sohnke SpGr)          R Verify
PLAT791_ALERT_4_G Model has Chirality at C94        (Sohnke SpGr)          R Verify
PLAT791_ALERT_4_G Model has Chirality at C105       (Sohnke SpGr)          S Verify
PLAT791_ALERT_4_G Model has Chirality at C108       (Sohnke SpGr)          R Verify
PLAT791_ALERT_4_G Model has Chirality at C115       (Sohnke SpGr)          S Verify
PLAT791_ALERT_4_G Model has Chirality at C117       (Sohnke SpGr)          R Verify
PLAT791_ALERT_4_G Model has Chirality at C122       (Sohnke SpGr)          R Verify
PLAT794_ALERT_5_G Tentative Bond Valency for Ag4       (I)       .       1.08 Info
PLAT794_ALERT_5_G Tentative Bond Valency for Ag11      (I)       .       1.18 Info
PLAT860_ALERT_3_G Number of Least-Squares Restraints ............         587 Note
PLAT883_ALERT_1_G No Info/Value for _atom_sites_solution_primary .     Please Do !
PLAT941_ALERT_3_G Average HKL Measurement Multiplicity ..........         2.1 Low
```

---

0 **ALERT level A** = Most likely a serious problem - resolve or explain

```
 2 ALERT level B = A potentially serious problem, consider carefully
56 ALERT level C = Check. Ensure it is not caused by an omission or oversight
78 ALERT level G = General information/check it is not something unexpected

11 ALERT type 1 CIF construction/syntax error, inconsistent or missing data
56 ALERT type 2 Indicator that the structure model may be wrong or deficient
12 ALERT type 3 Indicator that the structure quality may be low
54 ALERT type 4 Improvement, methodology, query or suggestion
 3 ALERT type 5 Informative message, check
```

Validation response form

Please find below a validation response form (VRF) that can be filled in and pasted into your CIF.

```
# start Validation Reply Form
_vrf_SHFSU01_4a
;
PROBLEM: The absolute value of parameter shift to su ratio > 0.05
RESPONSE: ...
;
_vrf_PLAT342_4a
;
PROBLEM: Low Bond Precision on  C-C Bonds ...............    0.04348 Ang.
RESPONSE: ...
;
_vrf_PLAT987_4a
;
PROBLEM: The Flack x is >> 0 -  Do a BASF/TWIN Refinement     Please Check
RESPONSE: ...
;
_vrf_PLAT080_4a
;
PROBLEM: Maximum Shift/Error ............................       0.08 Why ?
RESPONSE: ...
;
_vrf_PLAT213_4a
;
PROBLEM: Atom O4              has ADP max/min Ratio .....        3.3 oblate
RESPONSE: ...
;
_vrf_PLAT220_4a
;
PROBLEM: NonSolvent   Resd 1  C   Ueq(max)/Ueq(min) Range        4.4 Ratio
RESPONSE: ...
;
_vrf_PLAT222_4a
;
PROBLEM: NonSolvent Resd 1  H   Uiso(max)/Uiso(min) Range        4.6 Ratio
RESPONSE: ...
;
_vrf_PLAT234_4a
;
PROBLEM: Large Hirshfeld Difference S2       --C14      .       0.19 Ang.
RESPONSE: ...
;
```

```
_vrf_PLAT241_4a
;
PROBLEM: High   'MainMol' Ueq as Compared to Neighbors of        O14 Check
RESPONSE: ...
;
_vrf_PLAT242_4a
;
PROBLEM: Low    'MainMol' Ueq as Compared to Neighbors of        C10 Check
RESPONSE: ...
;
_vrf_PLAT243_4a
;
PROBLEM: High   'Solvent' Ueq as Compared to Neighbors of       C130 Check
RESPONSE: ...
;
_vrf_PLAT244_4a
;
PROBLEM: Low    'Solvent' Ueq as Compared to Neighbors of        C19 Check
RESPONSE: ...
;
_vrf_PLAT260_4a
;
PROBLEM: Large Average Ueq of Residue Including       Cl1      0.104 Check
RESPONSE: ...
;
_vrf_PLAT360_4a
;
PROBLEM: Short  C(sp3)-C(sp3) Bond  C48      - C78      .       1.42 Ang.
RESPONSE: ...
;
_vrf_PLAT361_4a
;
PROBLEM: Long   C(sp3)-C(sp3) Bond  C90      - C124     .       1.69 Ang.
RESPONSE: ...
;
_vrf_PLAT363_4a
;
PROBLEM: Long   C(sp3)-C(sp2) Bond  C58      - C112     .       1.63 Ang.
RESPONSE: ...
;
_vrf_PLAT410_4a
;
PROBLEM: Short Intra H...H Contact  H12F     ..H97B     .       1.99 Ang.
RESPONSE: ...
;
_vrf_PLAT767_4a
;
PROBLEM: INS Embedded LIST 6 Instruction Should be LIST 4     Please Check
RESPONSE: ...
;
# end Validation Reply Form
```

It is advisable to attempt to resolve as many as possible of the alerts in all categories. Often the minor alerts point to easily fixed oversights, errors and omissions in your CIF or refinement strategy, so attention to these fine details can be worthwhile. In order to resolve some of the more serious problems it may be necessary to carry out additional measurements or structure refinements. However, the purpose of your study may justify the reported deviations and the more serious of these should normally be commented upon in the discussion or experimental section of a paper or in the "special_details" fields of the CIF. checkCIF was carefully designed to identify outliers and unusual parameters, but every test has its limitations and alerts that are not important in a particular case may appear. Conversely, the absence of alerts does not guarantee there are no aspects of the results needing attention. It is up to the individual to critically assess their own results and, if necessary, seek expert advice.

Publication of your CIF in IUCr journals

A basic structural check has been run on your CIF. These basic checks will be run on all CIFs submitted for publication in IUCr journals (*Acta Crystallographica*, *Journal of Applied Crystallography*, *Journal of Synchrotron Radiation*); however, if you intend to submit to *Acta Crystallographica Section C* or *E* or *IUCrData*, you should make sure that full publication checks are run on the final version of your CIF prior to submission.

Publication of your CIF in other journals

Please refer to the *Notes for Authors* of the relevant journal for any special instructions relating to CIF submission.

PLATON version of 28/11/2022; check.def file version of 28/11/2022

Datablock 4a - ellipsoid plot

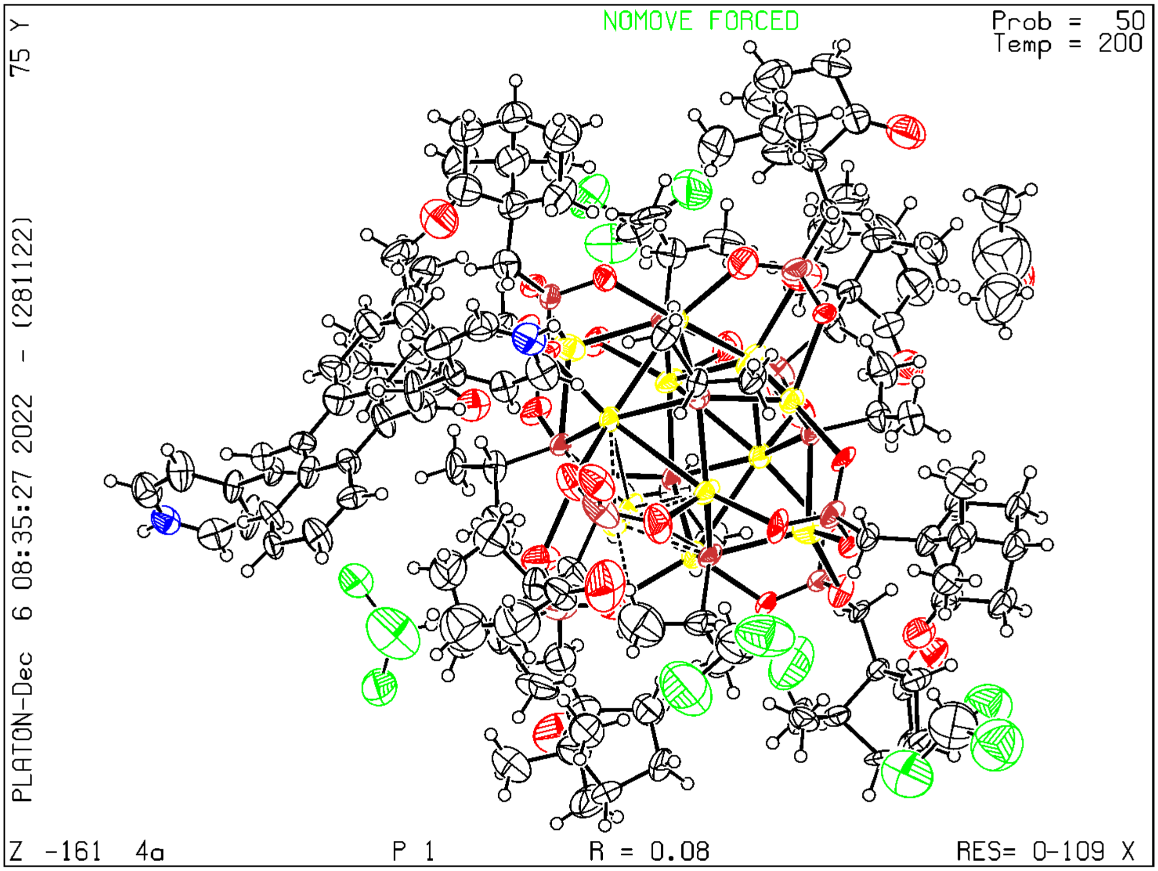

Supplement: Supplementary file 2 — Supporting Information [file ADVS-10-2207660-s002.zip › 4a-checkcif.pdf]
