# Supplementary material for: Stepwise Amplification of Circularly Polarized Luminescence in Chiral Metal Cluster Ensembles
Source: Adv Sci (Weinh). 2023 Feb 25;10(13):2207660. doi: 10.1002/advs.202207660 (PMC10161016; doi:10.1002/advs.202207660)

checkCIF/PLATON report

You have not supplied any structure factors. As a result the full set of tests cannot be run.

THIS REPORT IS FOR GUIDANCE ONLY. IF USED AS PART OF A REVIEW PROCEDURE FOR PUBLICATION, IT SHOULD NOT REPLACE THE EXPERTISE OF AN EXPERIENCED CRYSTALLOGRAPHIC REFEREE.

No syntax errors found. CIF dictionary Interpreting this report

Datablock: 4b

```
Bond precision:   C-C = 0.0352 A           Wavelength=1.54184

Cell:           a=15.6766(2)      b=17.5528(2)      c=17.8448(2)
                alpha=99.658(1)   beta=96.759(1)    gamma=116.466(1)
Temperature:    200 K
```

	Calculated	Reported
Volume	4229.11(10)	4229.10(9)
Space group	P 1	P 1
Hall group	P 1	P 1
Moiety formula	C98 H164 Ag12 O33 S14, C28 H22 N2, C H Cl3 [+ solvent]	C98 H164 Ag12 O33 S14, C H Cl3, C28 H22 N2
Sum formula	C127 H187 Ag12 Cl3 N2 O33 S14 [+ solvent]	C127 H187 Ag12 Cl3 N2 O33 S14
Mr	4119.42	4119.40
Dx,g cm-3	1.617	1.617
Z	1	1
Mu (mm-1)	13.445	13.445
F000	2066.0	2066.0
F000’	2078.13	
h,k,lmax	19,21,22	19,21,22
Nref	34326[17163]	31025
Tmin,Tmax	0.072,0.446	0.019,1.000
Tmin’	0.007	

```
Correction method= # Reported T Limits: Tmin=0.019 Tmax=1.000
AbsCorr = MULTI-SCAN

Data completeness= 1.81/0.90        Theta(max)= 73.942
```

R(reflections)= 0.0748(26469)   wR2(reflections)= 0.2188(31025)

S = 1.047   Npar= 1787

The following ALERTS were generated. Each ALERT has the format
test-name_ALERT_alert-type_alert-level.
Click on the hyperlinks for more details of the test.

Alert level B

PLAT342_ALERT_3_B Low Bond Precision on C-C Bonds .... 0.03523 Ang.
PLAT987_ALERT_1_B The Flack x is >> 0 - Do a BASF/TWIN Refinement Please Check

---

Alert level C

SHFSU01_ALERT_2_C The absolute value of parameter shift to su ratio > 0.05
Absolute value of the parameter shift to su ratio given 0.097
Additional refinement cycles may be required.
PLAT080_ALERT_2_C Maximum Shift/Error .... 0.10 Why ?
PLAT220_ALERT_2_C NonSolvent Resd 1 C Ueq(max)/Ueq(min) Range 3.8 Ratio
PLAT220_ALERT_2_C NonSolvent Resd 1 O Ueq(max)/Ueq(min) Range 3.4 Ratio
PLAT222_ALERT_3_C NonSolvent Resd 1 H Uiso(max)/Uiso(min) Range 4.6 Ratio
PLAT234_ALERT_4_C Large Hirshfeld Difference S11 --C2 . 0.19 Ang.
PLAT234_ALERT_4_C Large Hirshfeld Difference S13 --O27 . 0.20 Ang.
PLAT234_ALERT_4_C Large Hirshfeld Difference O24 --C29 . 0.22 Ang.
PLAT234_ALERT_4_C Large Hirshfeld Difference C39 --C86 . 0.19 Ang.
PLAT234_ALERT_4_C Large Hirshfeld Difference C111 --C116 . 0.21 Ang.
PLAT234_ALERT_4_C Large Hirshfeld Difference N1 --C42 . 0.25 Ang.
PLAT234_ALERT_4_C Large Hirshfeld Difference N2 --C75 . 0.24 Ang.
PLAT241_ALERT_2_C High 'MainMol' Ueq as Compared to Neighbors of O7 Check
PLAT241_ALERT_2_C High 'MainMol' Ueq as Compared to Neighbors of O9 Check
PLAT241_ALERT_2_C High 'MainMol' Ueq as Compared to Neighbors of O16 Check
PLAT241_ALERT_2_C High 'MainMol' Ueq as Compared to Neighbors of O17 Check
PLAT241_ALERT_2_C High 'MainMol' Ueq as Compared to Neighbors of O18 Check
PLAT241_ALERT_2_C High 'MainMol' Ueq as Compared to Neighbors of O21 Check
PLAT241_ALERT_2_C High 'MainMol' Ueq as Compared to Neighbors of C4 Check
PLAT241_ALERT_2_C High 'MainMol' Ueq as Compared to Neighbors of C34 Check
PLAT241_ALERT_2_C High 'MainMol' Ueq as Compared to Neighbors of C80 Check
PLAT241_ALERT_2_C High 'MainMol' Ueq as Compared to Neighbors of C85 Check
PLAT241_ALERT_2_C High 'MainMol' Ueq as Compared to Neighbors of C93 Check
PLAT241_ALERT_2_C High 'MainMol' Ueq as Compared to Neighbors of C110 Check
PLAT241_ALERT_2_C High 'MainMol' Ueq as Compared to Neighbors of C117 Check
PLAT241_ALERT_2_C High 'MainMol' Ueq as Compared to Neighbors of C126 Check
PLAT241_ALERT_2_C High 'MainMol' Ueq as Compared to Neighbors of C28 Check
PLAT241_ALERT_2_C High 'MainMol' Ueq as Compared to Neighbors of C42 Check
PLAT242_ALERT_2_C Low 'MainMol' Ueq as Compared to Neighbors of S11 Check
PLAT242_ALERT_2_C Low 'MainMol' Ueq as Compared to Neighbors of C3 Check
PLAT242_ALERT_2_C Low 'MainMol' Ueq as Compared to Neighbors of C29 Check
PLAT242_ALERT_2_C Low 'MainMol' Ueq as Compared to Neighbors of C38 Check
PLAT242_ALERT_2_C Low 'MainMol' Ueq as Compared to Neighbors of C39 Check
PLAT242_ALERT_2_C Low 'MainMol' Ueq as Compared to Neighbors of C47 Check
PLAT242_ALERT_2_C Low 'MainMol' Ueq as Compared to Neighbors of C62 Check
PLAT242_ALERT_2_C Low 'MainMol' Ueq as Compared to Neighbors of C66 Check
PLAT242_ALERT_2_C Low 'MainMol' Ueq as Compared to Neighbors of C67 Check
PLAT242_ALERT_2_C Low 'MainMol' Ueq as Compared to Neighbors of C91 Check

```
PLAT242_ALERT_2_C Low    'MainMol' Ueq as Compared to Neighbors of        C95 Check
PLAT242_ALERT_2_C Low    'MainMol' Ueq as Compared to Neighbors of       C106 Check
PLAT242_ALERT_2_C Low    'MainMol' Ueq as Compared to Neighbors of       C111 Check
PLAT242_ALERT_2_C Low    'MainMol' Ueq as Compared to Neighbors of       C112 Check
PLAT242_ALERT_2_C Low    'MainMol' Ueq as Compared to Neighbors of         N1 Check
PLAT260_ALERT_2_C Large Average Ueq of Residue Including       Cl1      0.134 Check
PLAT260_ALERT_2_C Large Average Ueq of Residue Including       Cl4      0.126 Check
PLAT360_ALERT_2_C Short  C(sp3)-C(sp3) Bond  C62      - C84      .       1.42 Ang.
PLAT360_ALERT_2_C Short  C(sp3)-C(sp3) Bond  C64      - C110     .       1.43 Ang.
PLAT410_ALERT_2_C Short Intra H...H Contact  H12B     ..H35B     .       1.97 Ang.
                                                      x,y,z  =      1_555 Check
PLAT412_ALERT_2_C Short Intra XH3 .. XHn     H12A     ..H12E     .       1.85 Ang.
                                                      x,y,z  =      1_555 Check
PLAT413_ALERT_2_C Short Inter XH3 .. XHn     H66B     ..H88B     .       2.12 Ang.
                                                    1+x,y,z  =      1_655 Check
PLAT767_ALERT_4_C INS Embedded LIST 6 Instruction Should be LIST 4     Please Check
```

Alert level G

```
PLAT002_ALERT_2_G Number of Distance or Angle Restraints on AtSite         31 Note
PLAT003_ALERT_2_G Number of Uiso or Uij Restrained non-H Atoms ...         55 Report
PLAT007_ALERT_5_G Number of Unrefined Donor-H Atoms .............           4 Report
PLAT033_ALERT_4_G Flack x Value Deviates > 3.0 * sigma from Zero .      0.080 Note
PLAT042_ALERT_1_G Calc. and Reported MoietyFormula Strings  Differ     Please Check
PLAT072_ALERT_2_G SHELXL First  Parameter in WGHT  Unusually Large       0.16 Report
PLAT154_ALERT_1_G The s.u.'s on the Cell Angles are Equal ..(Note)      0.001 Degree
PLAT172_ALERT_4_G The CIF-Embedded .res File Contains DFIX Records          9 Report
PLAT173_ALERT_4_G The CIF-Embedded .res File Contains DANG Records          5 Report
PLAT176_ALERT_4_G The CIF-Embedded .res File Contains SADI Records          1 Report
PLAT177_ALERT_4_G The CIF-Embedded .res File Contains DELU Records          1 Report
PLAT178_ALERT_4_G The CIF-Embedded .res File Contains SIMU Records          3 Report
PLAT186_ALERT_4_G The CIF-Embedded .res File Contains ISOR Records         15 Report
PLAT188_ALERT_3_G A Non-default SIMU Restraint Value has been used     0.0100 Report
PLAT188_ALERT_3_G A Non-default SIMU Restraint Value has been used     0.0100 Report
PLAT188_ALERT_3_G A Non-default SIMU Restraint Value has been used     0.0100 Report
PLAT191_ALERT_3_G A Non-default SADI Restraint Value has been used     0.0100 Report
PLAT232_ALERT_2_G Hirshfeld Test Diff (M-X)  Ag5      --S6       .        5.7 s.u.
PLAT232_ALERT_2_G Hirshfeld Test Diff (M-X)  Ag10     --S10      .        6.1 s.u.
PLAT300_ALERT_4_G Atom Site Occupancy of C63        Constrained at        0.5 Check
PLAT300_ALERT_4_G Atom Site Occupancy of C68        Constrained at        0.5 Check
PLAT300_ALERT_4_G Atom Site Occupancy of C128       Constrained at        0.5 Check
PLAT300_ALERT_4_G Atom Site Occupancy of C129       Constrained at        0.5 Check
PLAT300_ALERT_4_G Atom Site Occupancy of H12M       Constrained at        0.5 Check
PLAT300_ALERT_4_G Atom Site Occupancy of H12N       Constrained at        0.5 Check
PLAT300_ALERT_4_G Atom Site Occupancy of H12O       Constrained at        0.5 Check
PLAT300_ALERT_4_G Atom Site Occupancy of H12P       Constrained at        0.5 Check
PLAT300_ALERT_4_G Atom Site Occupancy of H12Q       Constrained at        0.5 Check
PLAT300_ALERT_4_G Atom Site Occupancy of H12R       Constrained at        0.5 Check
PLAT300_ALERT_4_G Atom Site Occupancy of H18        Constrained at        0.5 Check
PLAT300_ALERT_4_G Atom Site Occupancy of H18A       Constrained at        0.5 Check
PLAT300_ALERT_4_G Atom Site Occupancy of H63A       Constrained at        0.5 Check
PLAT300_ALERT_4_G Atom Site Occupancy of H63B       Constrained at        0.5 Check
PLAT300_ALERT_4_G Atom Site Occupancy of H63C       Constrained at        0.5 Check
PLAT300_ALERT_4_G Atom Site Occupancy of H68A       Constrained at        0.5 Check
PLAT300_ALERT_4_G Atom Site Occupancy of H68B       Constrained at        0.5 Check
PLAT300_ALERT_4_G Atom Site Occupancy of H68C       Constrained at        0.5 Check
PLAT300_ALERT_4_G Atom Site Occupancy of Cl1        Constrained at       0.75 Check
```

```
PLAT300_ALERT_4_G Atom Site Occupancy of Cl2        Constrained at       0.75 Check
PLAT300_ALERT_4_G Atom Site Occupancy of Cl3        Constrained at       0.75 Check
PLAT300_ALERT_4_G Atom Site Occupancy of C100       Constrained at       0.75 Check
PLAT300_ALERT_4_G Atom Site Occupancy of H100       Constrained at       0.75 Check
PLAT300_ALERT_4_G Atom Site Occupancy of Cl4        Constrained at       0.25 Check
PLAT300_ALERT_4_G Atom Site Occupancy of Cl5        Constrained at       0.25 Check
PLAT300_ALERT_4_G Atom Site Occupancy of Cl6        Constrained at       0.25 Check
PLAT300_ALERT_4_G Atom Site Occupancy of C130       Constrained at       0.25 Check
PLAT300_ALERT_4_G Atom Site Occupancy of H130       Constrained at       0.25 Check
PLAT301_ALERT_3_G Main Residue  Disorder .............(Resd  1  )         1% Note
PLAT302_ALERT_4_G Anion/Solvent/Minor-Residue Disorder (Resd  3  )       100% Note
PLAT302_ALERT_4_G Anion/Solvent/Minor-Residue Disorder (Resd  4  )       100% Note
PLAT304_ALERT_4_G Non-Integer Number of Atoms in ..... (Resd  3  )       3.75 Check
PLAT304_ALERT_4_G Non-Integer Number of Atoms in ..... (Resd  4  )       1.25 Check
PLAT343_ALERT_2_G Unusual sp3      Angle Range in Main Residue for       C127 Check
PLAT606_ALERT_4_G Solvent Accessible VOID(S) in Structure ........          ! Info
PLAT721_ALERT_1_G Bond    Calc     0.97000, Rep     0.98010 Dev...       0.01 Ang.
              C25      -H25A           1_555   1_555 ........     #  148 Check
PLAT721_ALERT_1_G Bond    Calc     0.99000, Rep     0.97980 Dev...       0.01 Ang.
              C25      -H25B           1_555   1_555 ........     #  149 Check
PLAT721_ALERT_1_G Bond    Calc     0.99000, Rep     0.97950 Dev...       0.01 Ang.
              C127     -H12L           1_555   1_555 ........     #  372 Check
PLAT722_ALERT_1_G Angle   Calc      113.00, Rep      111.80 Dev...       1.20 Degree
              C73     -C34     -H34A       1_555  1_555  1_555     #  444 Check
PLAT722_ALERT_1_G Angle   Calc      111.00, Rep      109.50 Dev...       1.50 Degree
              H68B    -C68     -H68C       1_555  1_555  1_555     #  601 Check
PLAT722_ALERT_1_G Angle   Calc      103.00, Rep      104.10 Dev...       1.10 Degree
              C18     -C128    -H12N       1_555  1_555  1_555     #  900 Check
PLAT790_ALERT_4_G Centre of Gravity not Within Unit Cell: Resd.  #          4 Note
              C H Cl3
PLAT791_ALERT_4_G Model has Chirality at C1          (Sohnke SpGr)          R Verify
PLAT791_ALERT_4_G Model has Chirality at C20         (Sohnke SpGr)          S Verify
PLAT791_ALERT_4_G Model has Chirality at C22         (Sohnke SpGr)          R Verify
PLAT791_ALERT_4_G Model has Chirality at C39         (Sohnke SpGr)          S Verify
PLAT791_ALERT_4_G Model has Chirality at C41         (Sohnke SpGr)          S Verify
PLAT791_ALERT_4_G Model has Chirality at C46         (Sohnke SpGr)          R Verify
PLAT791_ALERT_4_G Model has Chirality at C47         (Sohnke SpGr)          R Verify
PLAT791_ALERT_4_G Model has Chirality at C51         (Sohnke SpGr)          S Verify
PLAT791_ALERT_4_G Model has Chirality at C59         (Sohnke SpGr)          S Verify
PLAT791_ALERT_4_G Model has Chirality at C64         (Sohnke SpGr)          S Verify
PLAT791_ALERT_4_G Model has Chirality at C78         (Sohnke SpGr)          S Verify
PLAT791_ALERT_4_G Model has Chirality at C83         (Sohnke SpGr)          R Verify
PLAT791_ALERT_4_G Model has Chirality at C90         (Sohnke SpGr)          R Verify
PLAT791_ALERT_4_G Model has Chirality at C91         (Sohnke SpGr)          R Verify
PLAT791_ALERT_4_G Model has Chirality at C111        (Sohnke SpGr)          S Verify
PLAT791_ALERT_4_G Model has Chirality at C115        (Sohnke SpGr)          R Verify
PLAT794_ALERT_5_G Tentative Bond Valency for Ag8       (I)       .       1.20 Info
PLAT794_ALERT_5_G Tentative Bond Valency for Ag11      (I)       .       1.07 Info
PLAT860_ALERT_3_G Number of Least-Squares Restraints ............         481 Note
PLAT883_ALERT_1_G No Info/Value for _atom_sites_solution_primary .     Please Do !
PLAT933_ALERT_2_G Number of HKL-OMIT Records in Embedded .res File          2 Note
```

---

0 **ALERT level A** = Most likely a serious problem - resolve or explain
2 **ALERT level B** = A potentially serious problem, consider carefully
51 **ALERT level C** = Check. Ensure it is not caused by an omission or oversight
82 **ALERT level G** = General information/check it is not something unexpected

```
  10 ALERT type 1 CIF construction/syntax error, inconsistent or missing data
  49 ALERT type 2 Indicator that the structure model may be wrong or deficient
   8 ALERT type 3 Indicator that the structure quality may be low
  65 ALERT type 4 Improvement, methodology, query or suggestion
   3 ALERT type 5 Informative message, check
```

Validation response form

Please find below a validation response form (VRF) that can be filled in and pasted into your CIF.

```
# start Validation Reply Form
_vrf_SHFSU01_4b
;
PROBLEM: The absolute value of parameter shift to su ratio > 0.05
RESPONSE: ...
;
_vrf_PLAT342_4b
;
PROBLEM: Low Bond Precision on  C-C Bonds ...............    0.03523 Ang.
RESPONSE: ...
;
_vrf_PLAT987_4b
;
PROBLEM: The Flack x is >> 0 -  Do a BASF/TWIN Refinement     Please Check
RESPONSE: ...
;
_vrf_PLAT080_4b
;
PROBLEM: Maximum Shift/Error ...........................       0.10 Why ?
RESPONSE: ...
;
_vrf_PLAT220_4b
;
PROBLEM: NonSolvent   Resd 1  C   Ueq(max)/Ueq(min) Range        3.8 Ratio
RESPONSE: ...
;
_vrf_PLAT222_4b
;
PROBLEM: NonSolvent Resd 1  H   Uiso(max)/Uiso(min) Range        4.6 Ratio
RESPONSE: ...
;
_vrf_PLAT234_4b
;
PROBLEM: Large Hirshfeld Difference S11      --C2       .       0.19 Ang.
RESPONSE: ...
;
_vrf_PLAT241_4b
;
PROBLEM: High   'MainMol' Ueq as Compared to Neighbors of         O7 Check
RESPONSE: ...
;
_vrf_PLAT242_4b
;
PROBLEM: Low    'MainMol' Ueq as Compared to Neighbors of        S11 Check
```

```
RESPONSE: ...
;
_vrf_PLAT260_4b
;
PROBLEM: Large Average Ueq of Residue Including       Cl1      0.134 Check
RESPONSE: ...
;
_vrf_PLAT360_4b
;
PROBLEM: Short  C(sp3)-C(sp3) Bond  C62      - C84      .       1.42 Ang.
RESPONSE: ...
;
_vrf_PLAT410_4b
;
PROBLEM: Short Intra H...H Contact  H12B     ..H35B     .       1.97 Ang.
RESPONSE: ...
;
_vrf_PLAT412_4b
;
PROBLEM: Short Intra XH3 .. XHn     H12A     ..H12E     .       1.85 Ang.
RESPONSE: ...
;
_vrf_PLAT413_4b
;
PROBLEM: Short Inter XH3 .. XHn     H66B     ..H88B     .       2.12 Ang.
RESPONSE: ...
;
_vrf_PLAT767_4b
;
PROBLEM: INS Embedded LIST 6 Instruction Should be LIST 4     Please Check
RESPONSE: ...
;
# end Validation Reply Form
```

It is advisable to attempt to resolve as many as possible of the alerts in all categories. Often the minor alerts point to easily fixed oversights, errors and omissions in your CIF or refinement strategy, so attention to these fine details can be worthwhile. In order to resolve some of the more serious problems it may be necessary to carry out additional measurements or structure refinements. However, the purpose of your study may justify the reported deviations and the more serious of these should normally be commented upon in the discussion or experimental section of a paper or in the "special_details" fields of the CIF. checkCIF was carefully designed to identify outliers and unusual parameters, but every test has its limitations and alerts that are not important in a particular case may appear. Conversely, the absence of alerts does not guarantee there are no aspects of the results needing attention. It is up to the individual to critically assess their own results and, if necessary, seek expert advice.

Publication of your CIF in IUCr journals

A basic structural check has been run on your CIF. These basic checks will be run on all CIFs submitted for publication in IUCr journals (*Acta Crystallographica*, *Journal of Applied Crystallography*, *Journal of Synchrotron Radiation*); however, if you intend to submit to *Acta Crystallographica Section C* or *E* or *IUCrData*, you should make sure that full publication checks are run on the final version of your CIF prior to submission.

Publication of your CIF in other journals

Please refer to the *Notes for Authors* of the relevant journal for any special instructions relating to CIF submission.

PLATON version of 28/11/2022; check.def file version of 28/11/2022

Datablock 4b - ellipsoid plot

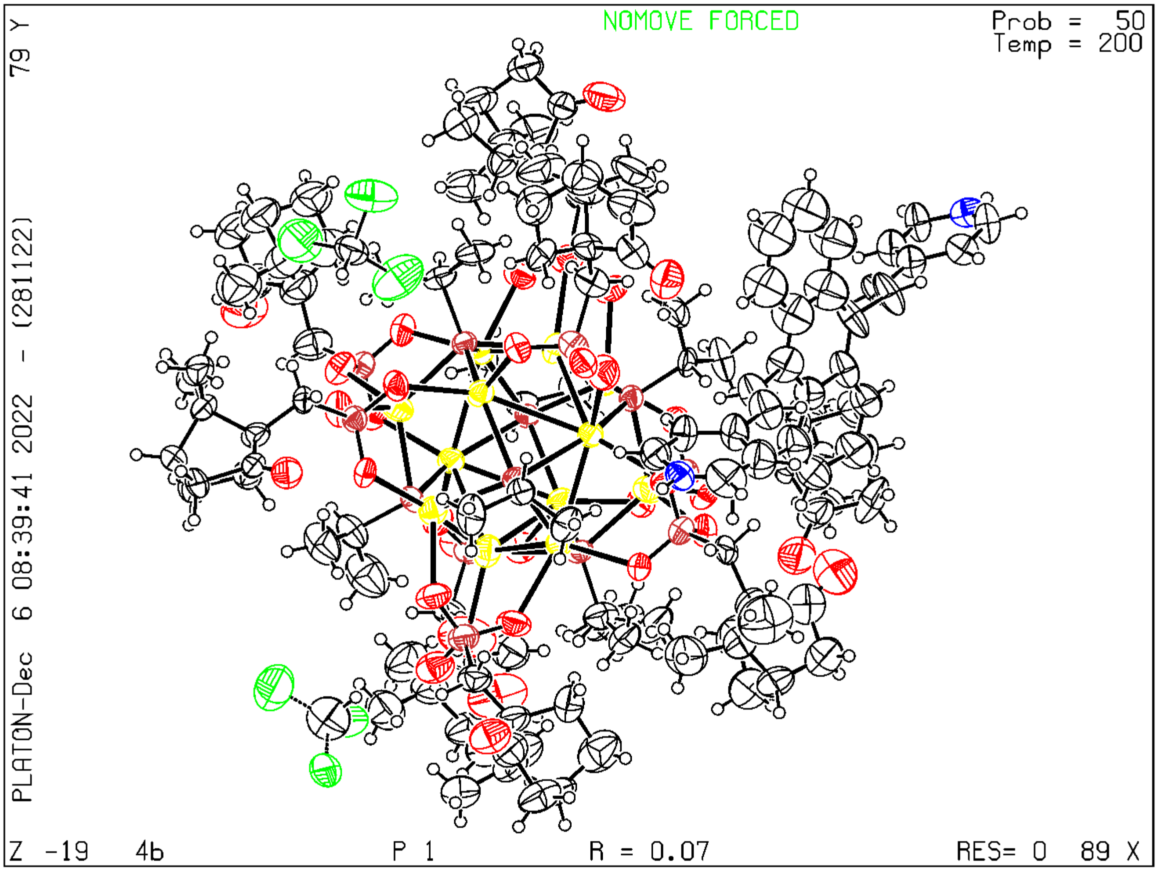

Supplement: Supplementary file 2 — Supporting Information [file ADVS-10-2207660-s002.zip › 4b-checkcif.pdf]
